# Supplementary material for: Cell neighborhood topology directs rare cell population identification
Source: Nat Commun. 2026 Mar 30;17:4618. doi: 10.1038/s41467-026-71180-x (PMC13199379; doi:10.1038/s41467-026-71180-x)
Supplement: Supplementary file 1 — Supplementary Information [file 41467_2026_71180_MOESM1_ESM.pdf]

Supplemental information for

Cell Neighborhood Topology Directs Rare Cell Population Identification

*Corresponding author: Zhengtao Xiao (zhengtao.xiao@xjtu.edu.cn)

This file includes:

Supplementary Figure 1-72
Supplementary Table 1-6
Supplementary Note 1-7

Supplementary Figures

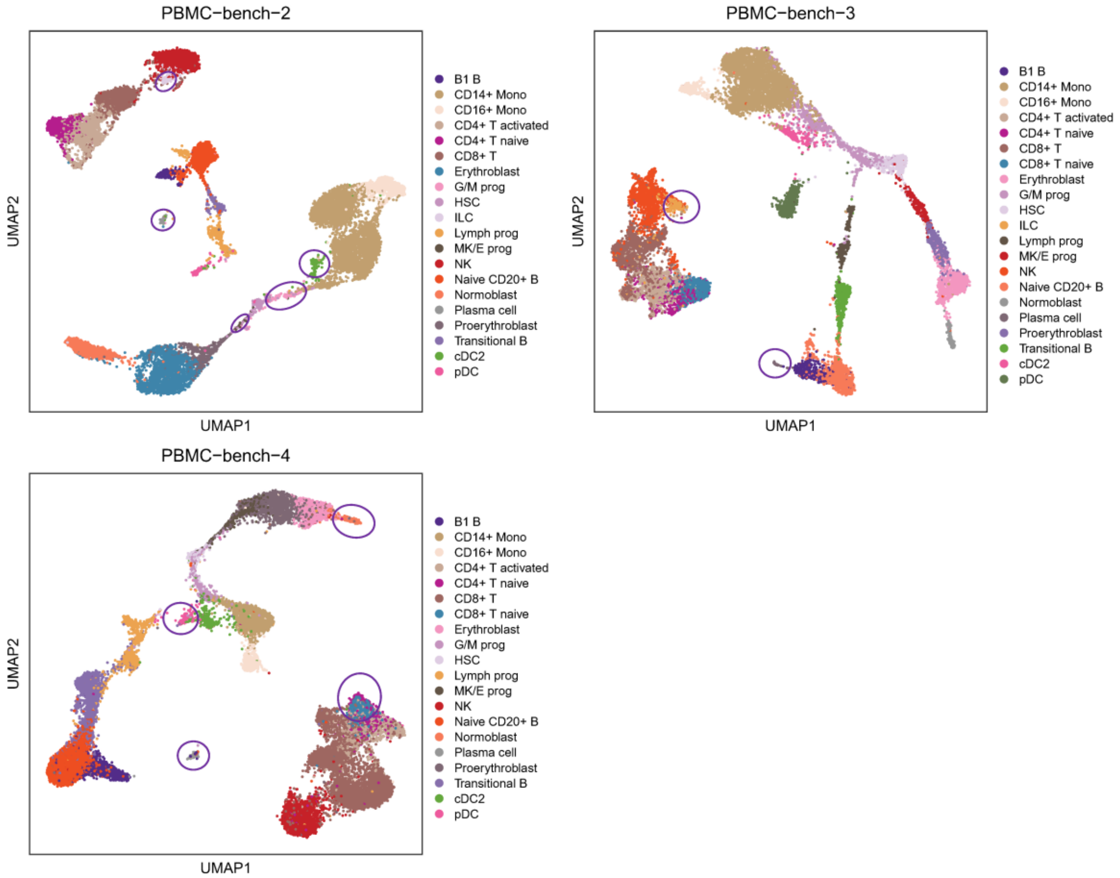

Supplementary Fig. 1 UMAPs visualizing the rare cell types (< 1% total cells, highlighted in purple circles) in the PBMC-bench-2, 3, and 4 datasets. Source data are provided as a Source Data file.

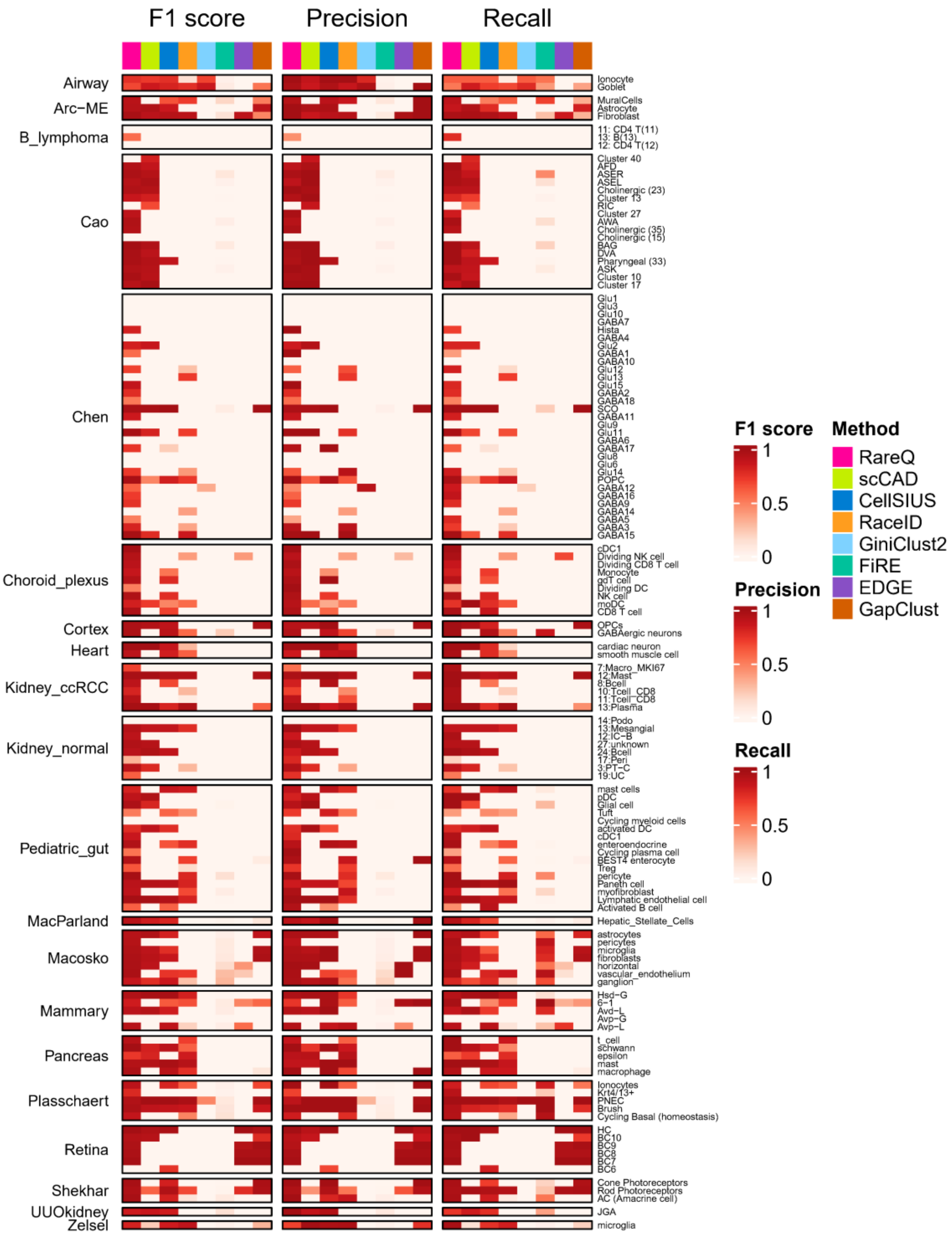

Supplementary Fig. 2 Rare cell population (< 1% population) identification on 20 real scRNA-seq datasets benchmarked with existing rare cell detection tools evaluated via F_1 scores, Precision and Recall. The rows denote rare cell types in specified datasets, while the columns signify the specified methods. Source data are provided as a Source Data file.

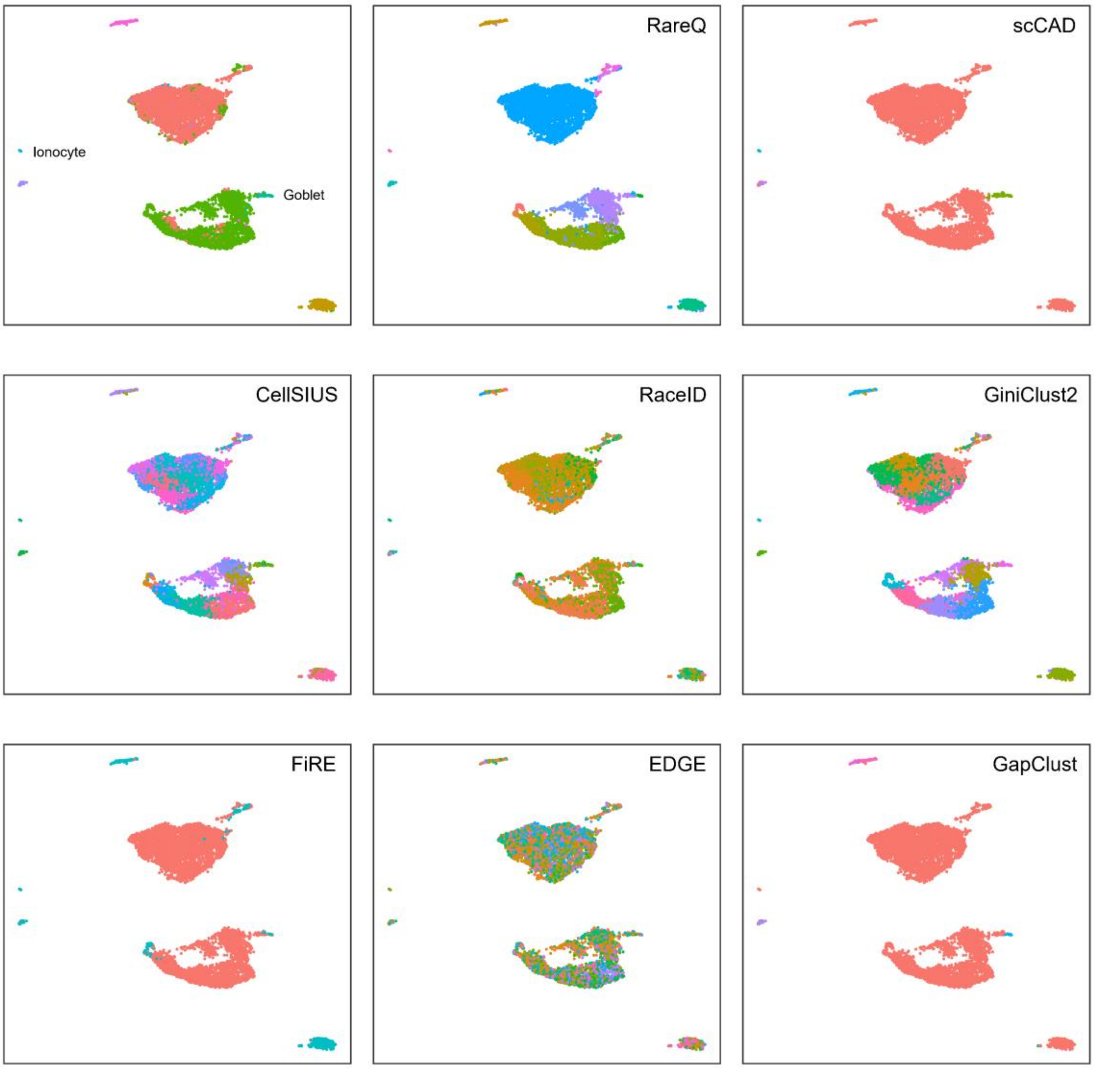

Supplementary Fig. 3 The UMAP 2D embedding of the cells in the Airway dataset with rare cell types (< 1% population) highlighted. Rare cell clusters predicted by the specified methods are labeled with different colors. Source data are provided as a Source Data file.

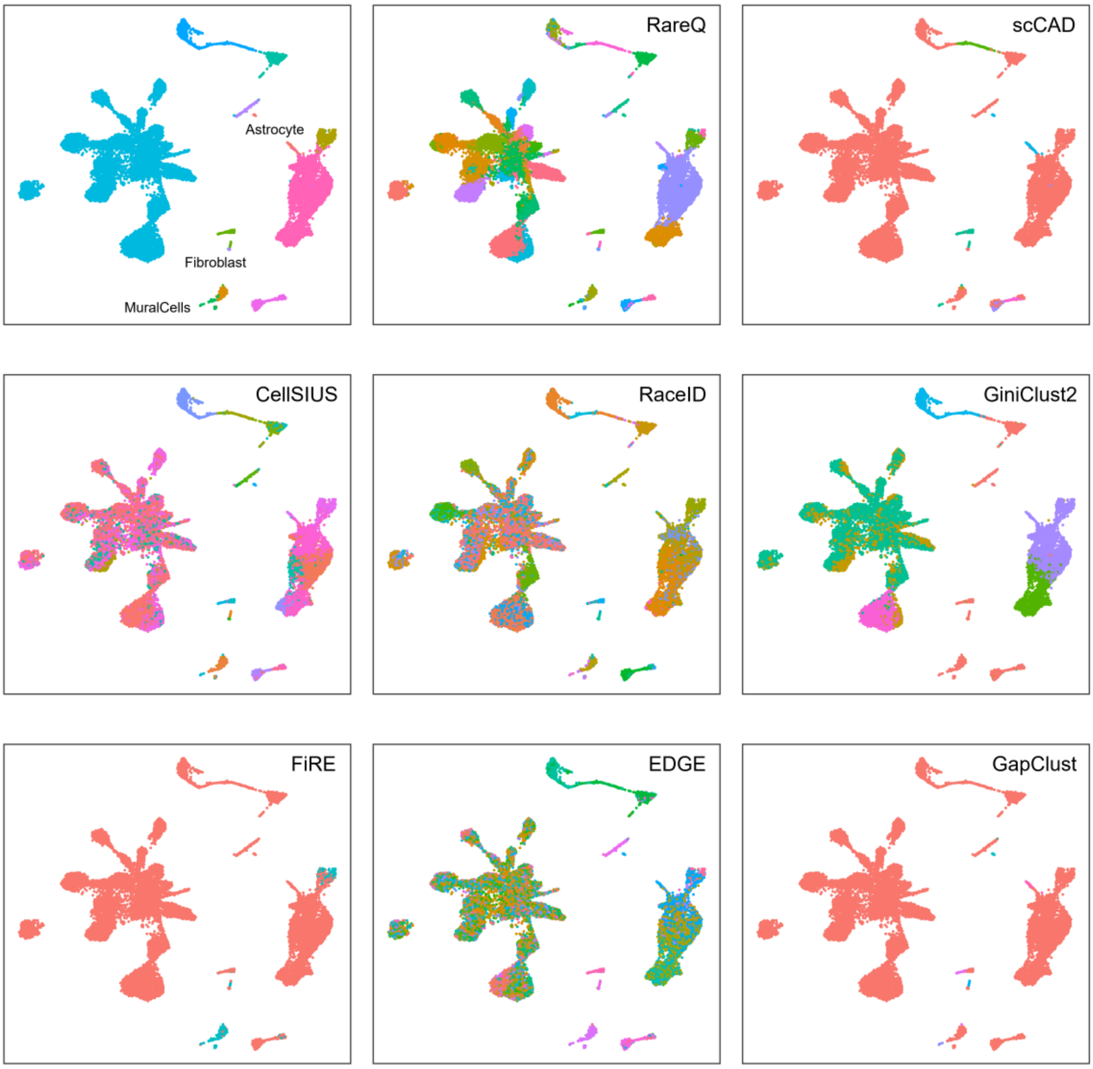

Supplementary Fig. 4 The UMAP 2D embedding of the cells in the Arc-ME dataset with rare cell types (< 1% population) highlighted. Rare cell clusters predicted by the specified methods are labeled with different colors. Source data are provided as a Source Data file.

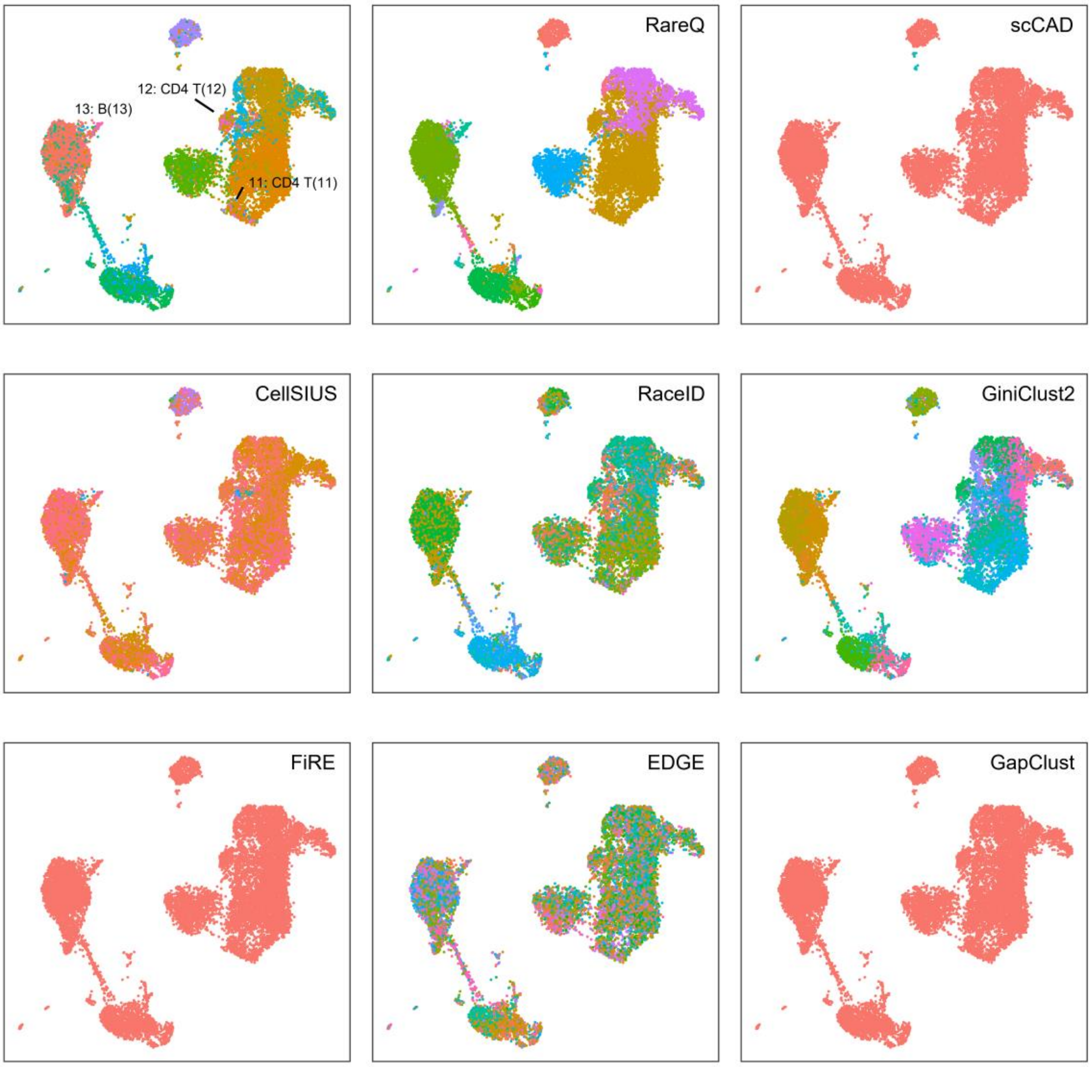

Supplementary Fig. 5 The UMAP 2D embedding of the cells in the B_lymphoma dataset with rare cell types (< 1% population) highlighted. Rare cell clusters predicted by the specified methods are labeled with different colors. Source data are provided as a Source Data file.

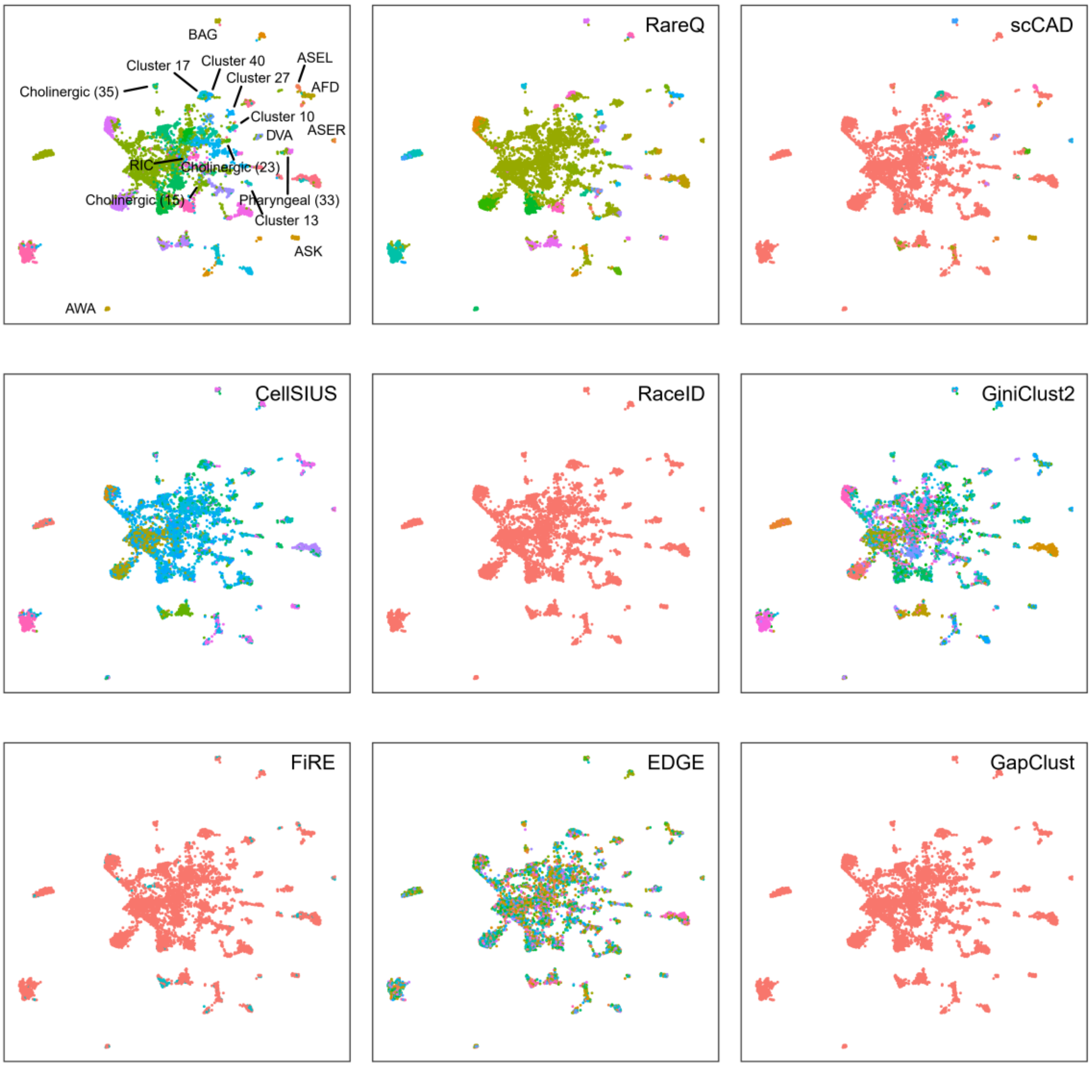

Supplementary Fig. 6 The UMAP 2D embedding of the cells in the Cao dataset with rare cell types (< 1% population) highlighted. Rare cell clusters predicted by the specified methods are labeled with different colors. Source data are provided as a Source Data file.

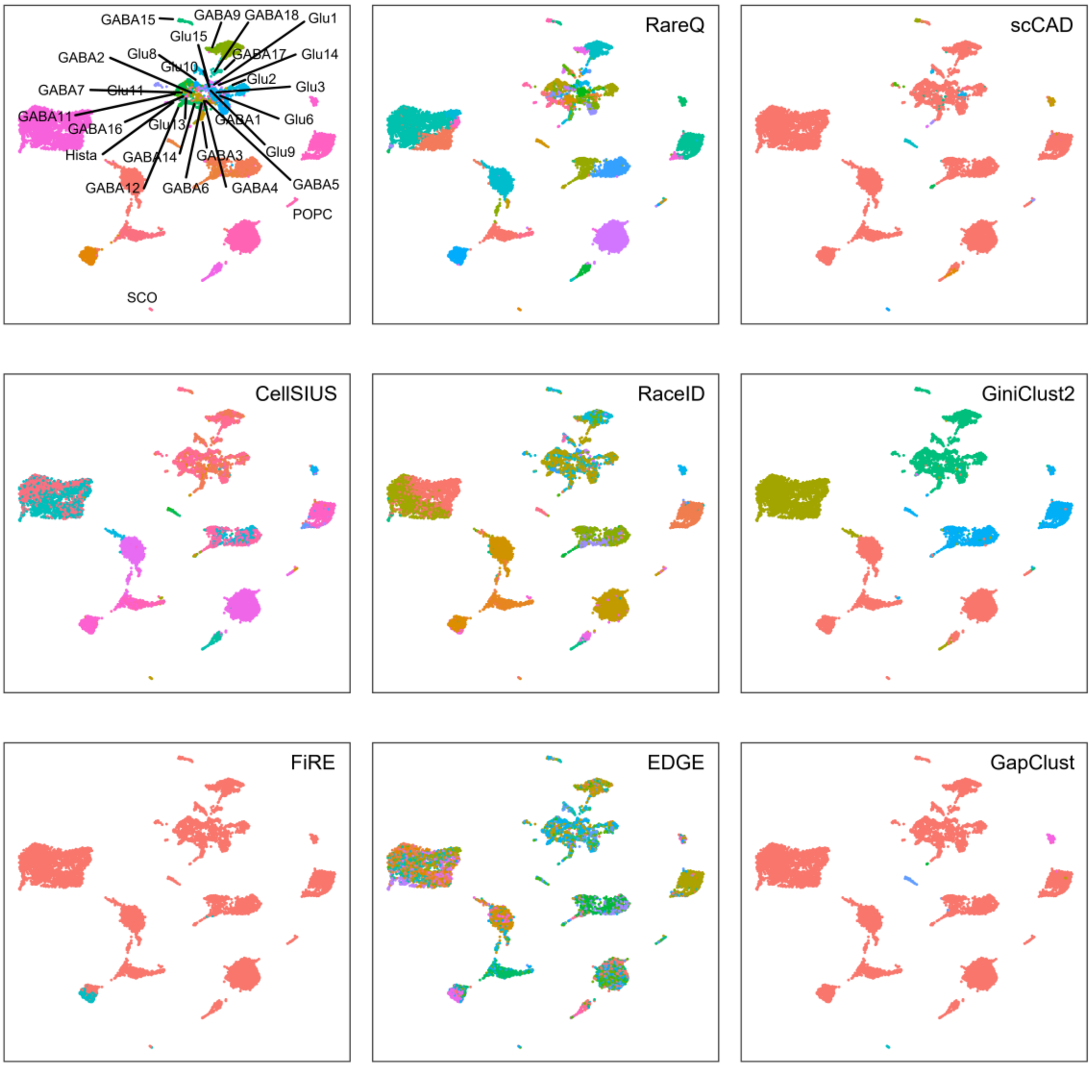

Supplementary Fig. 7 The UMAP 2D embedding of the cells in the Chen dataset with rare cell types (< 1% population) highlighted. Rare cell clusters predicted by the specified methods are labeled with different colors. Source data are provided as a Source Data file.

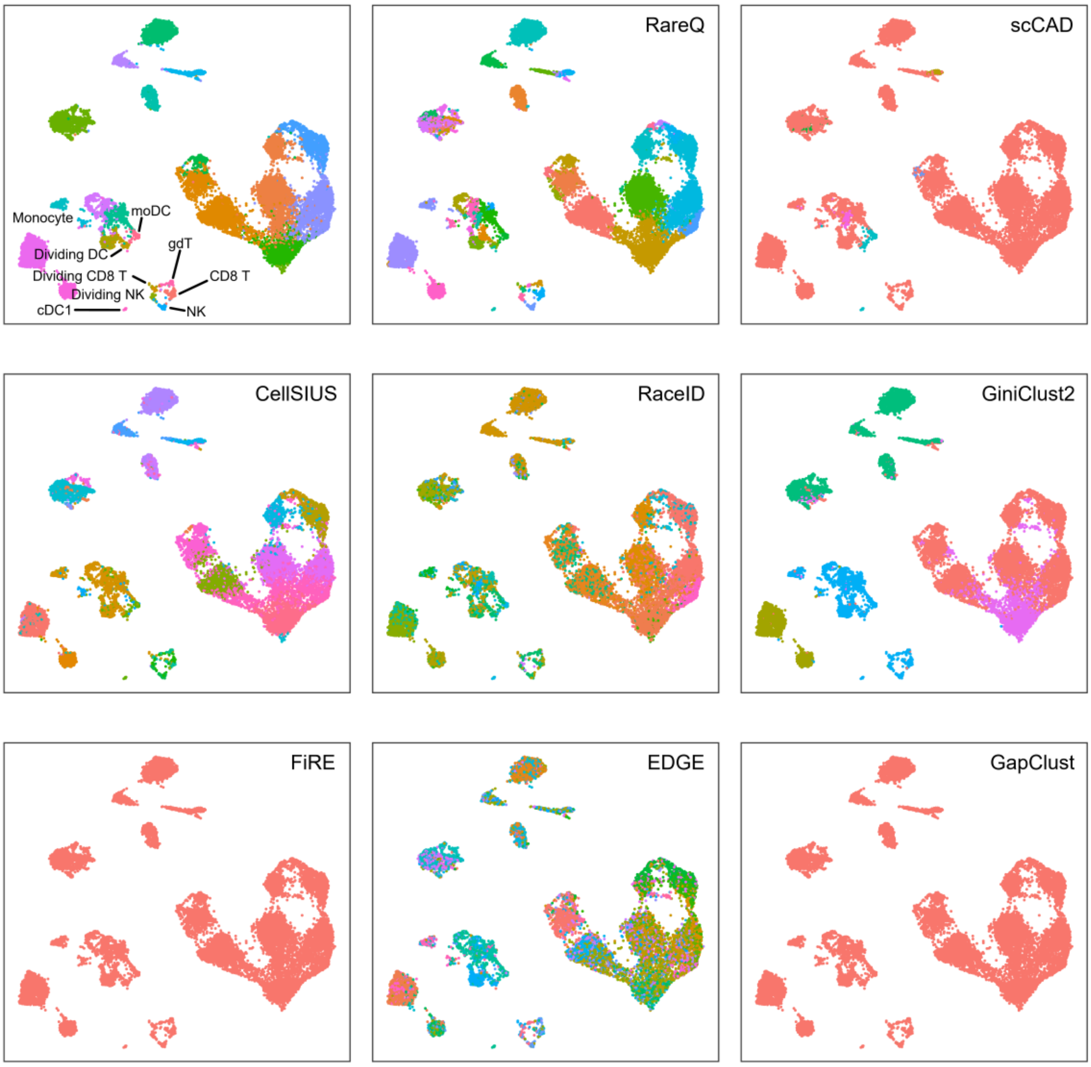

Supplementary Fig. 8 The UMAP 2D embedding of the cells in the Choroid_plexus dataset with rare cell types (< 1% population) highlighted. Rare cell clusters predicted by the specified methods are labeled with different colors. Source data are provided as a Source Data file.

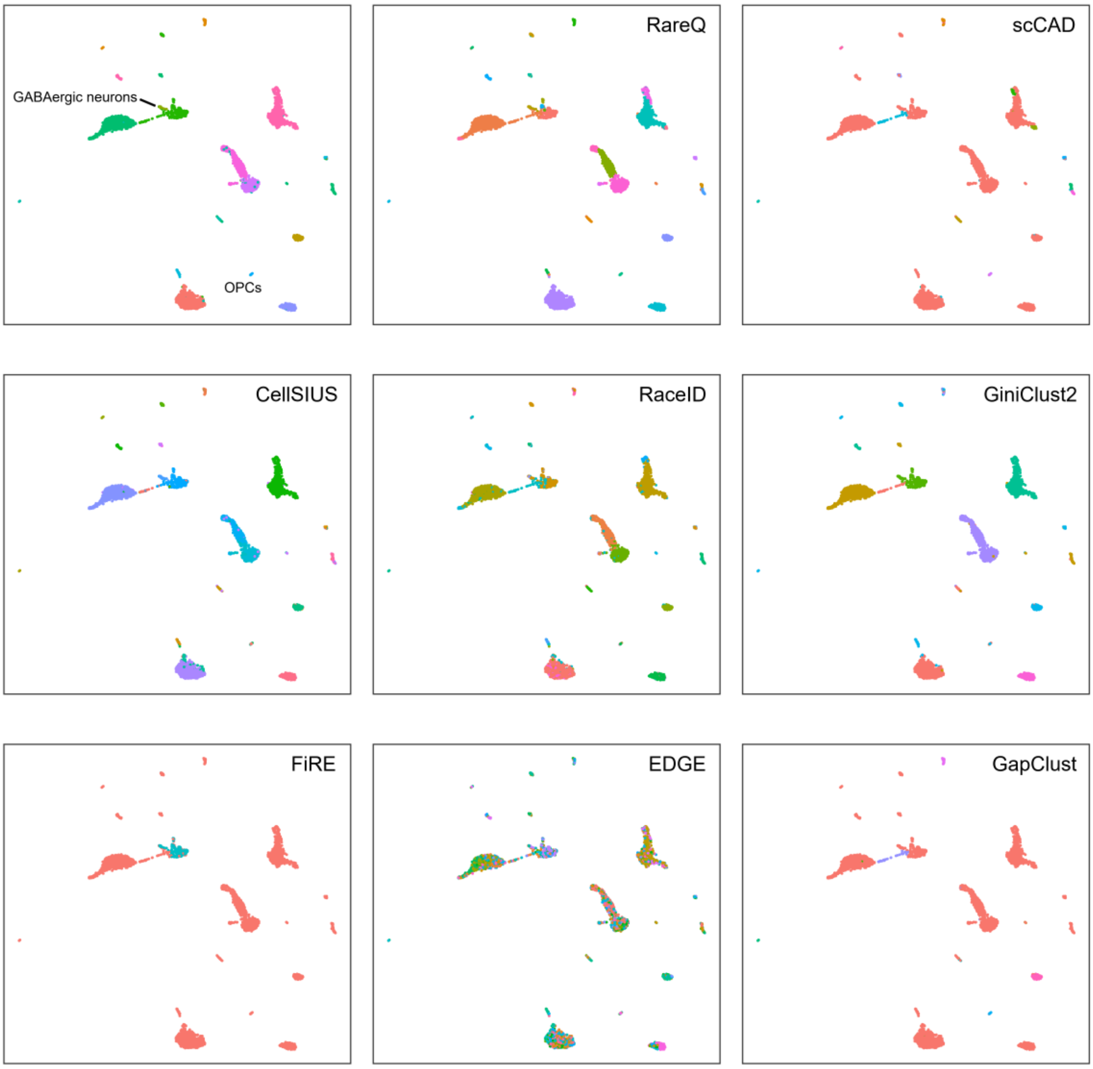

Supplementary Fig. 9 The UMAP 2D embedding of the cells in the Cortex dataset with rare cell types (< 1% population) highlighted. Rare cell clusters predicted by the specified methods are labeled with different colors. Source data are provided as a Source Data file.

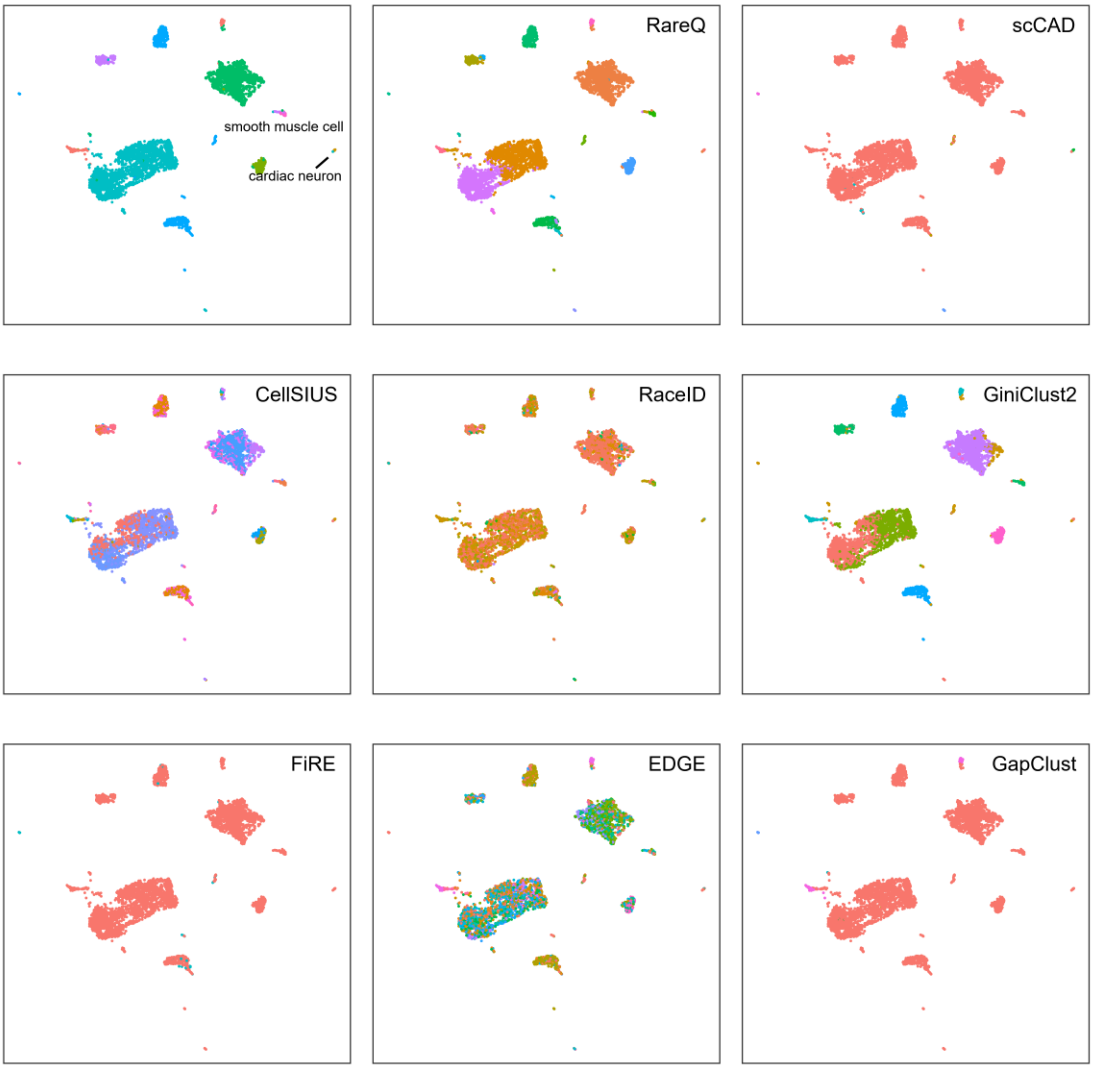

Supplementary Fig. 10 The UMAP 2D embedding of the cells in the Heart dataset with rare cell types (< 1% population) highlighted. Rare cell clusters predicted by the specified methods are labeled with different colors. Source data are provided as a Source Data file.

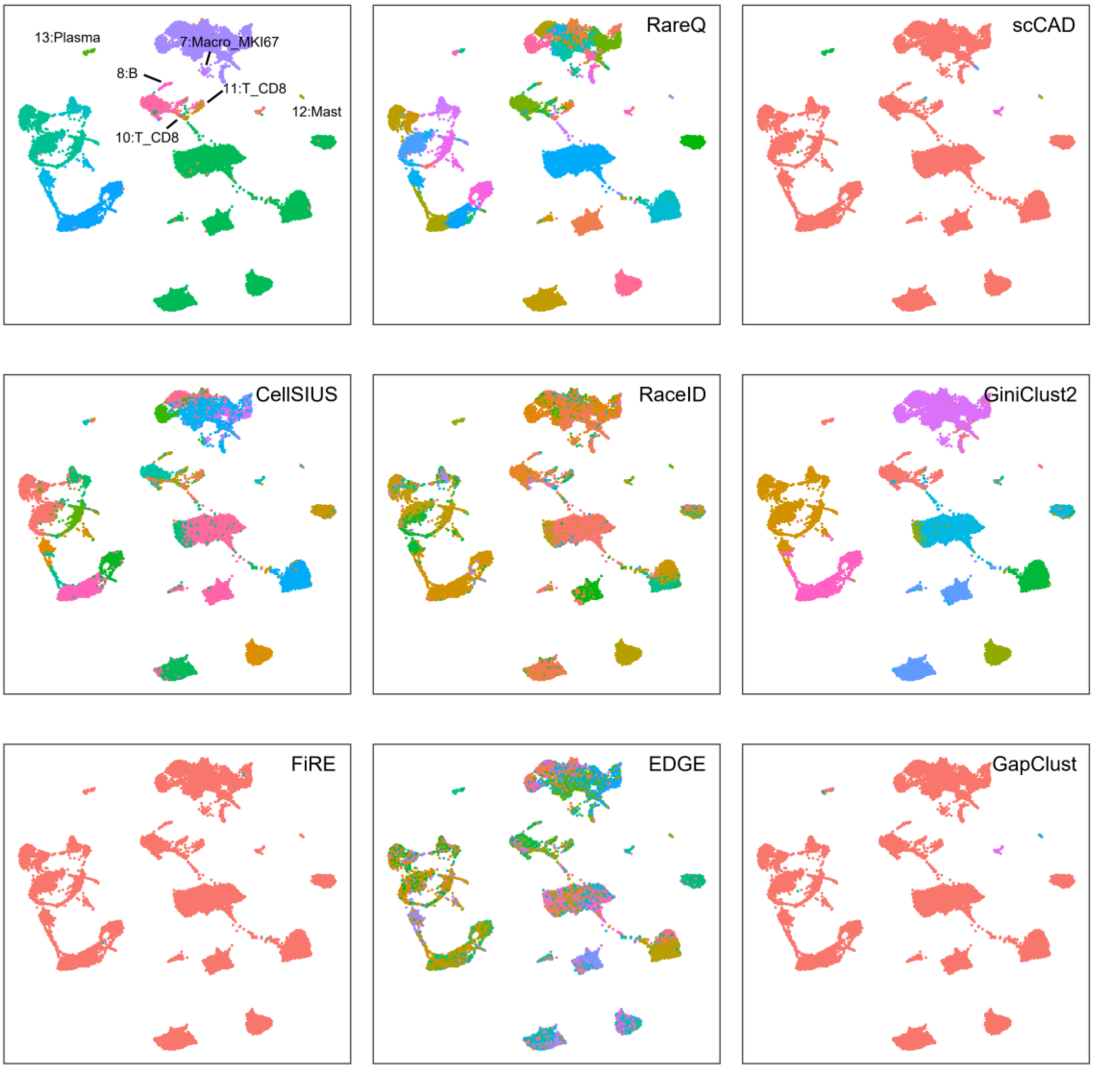

Supplementary Fig. 11 The UMAP 2D embedding of the cells in the Kidney_ccRCC dataset with rare cell types (< 1% population) highlighted. Rare cell clusters predicted by the specified methods are labeled with different colors. Source data are provided as a Source Data file.

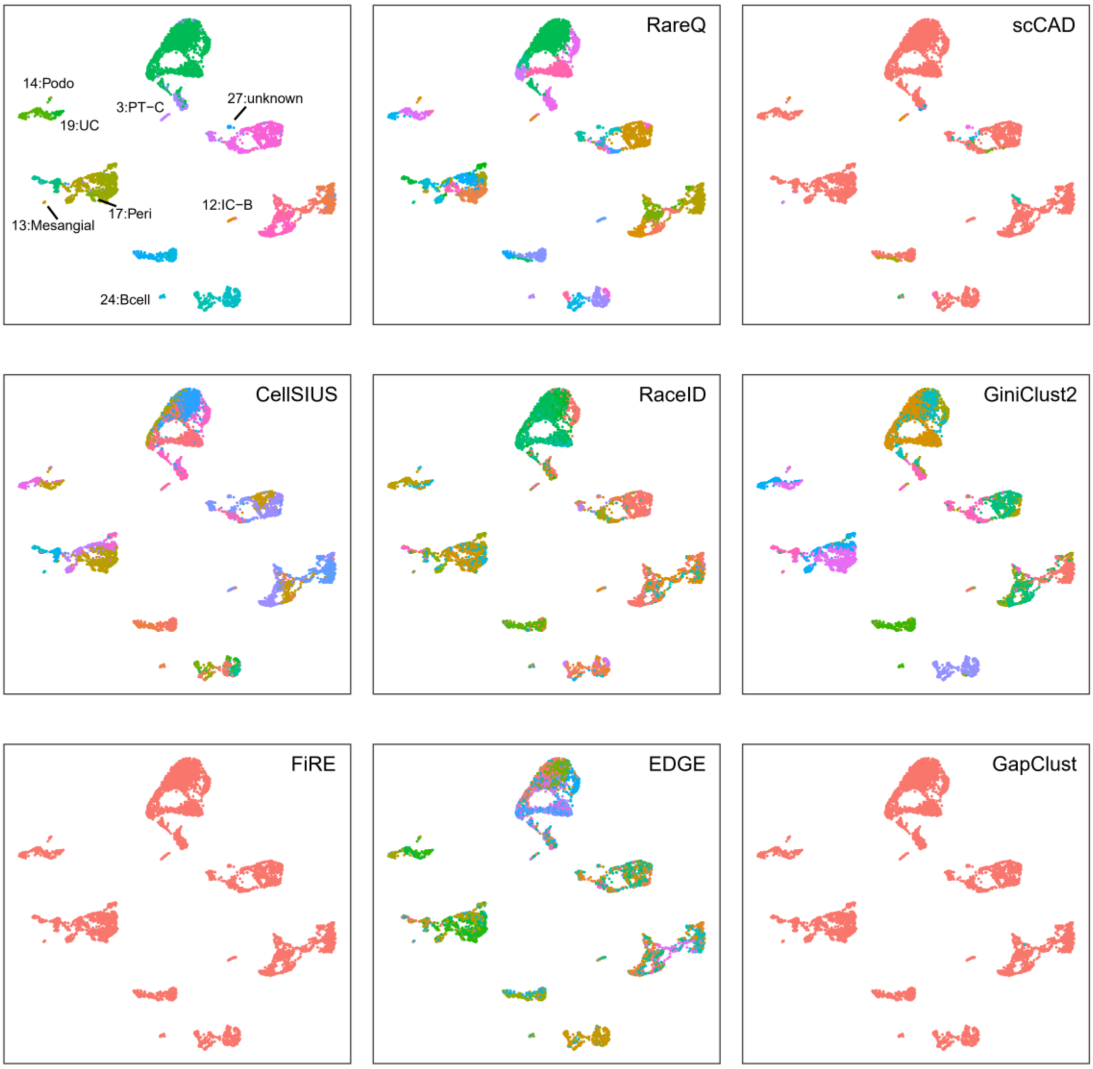

Supplementary Fig. 12 The UMAP 2D embedding of the cells in the Kidney_normal dataset with rare cell types (< 1% population) highlighted. Rare cell clusters predicted by the specified methods are labeled with different colors. Source data are provided as a Source Data file.

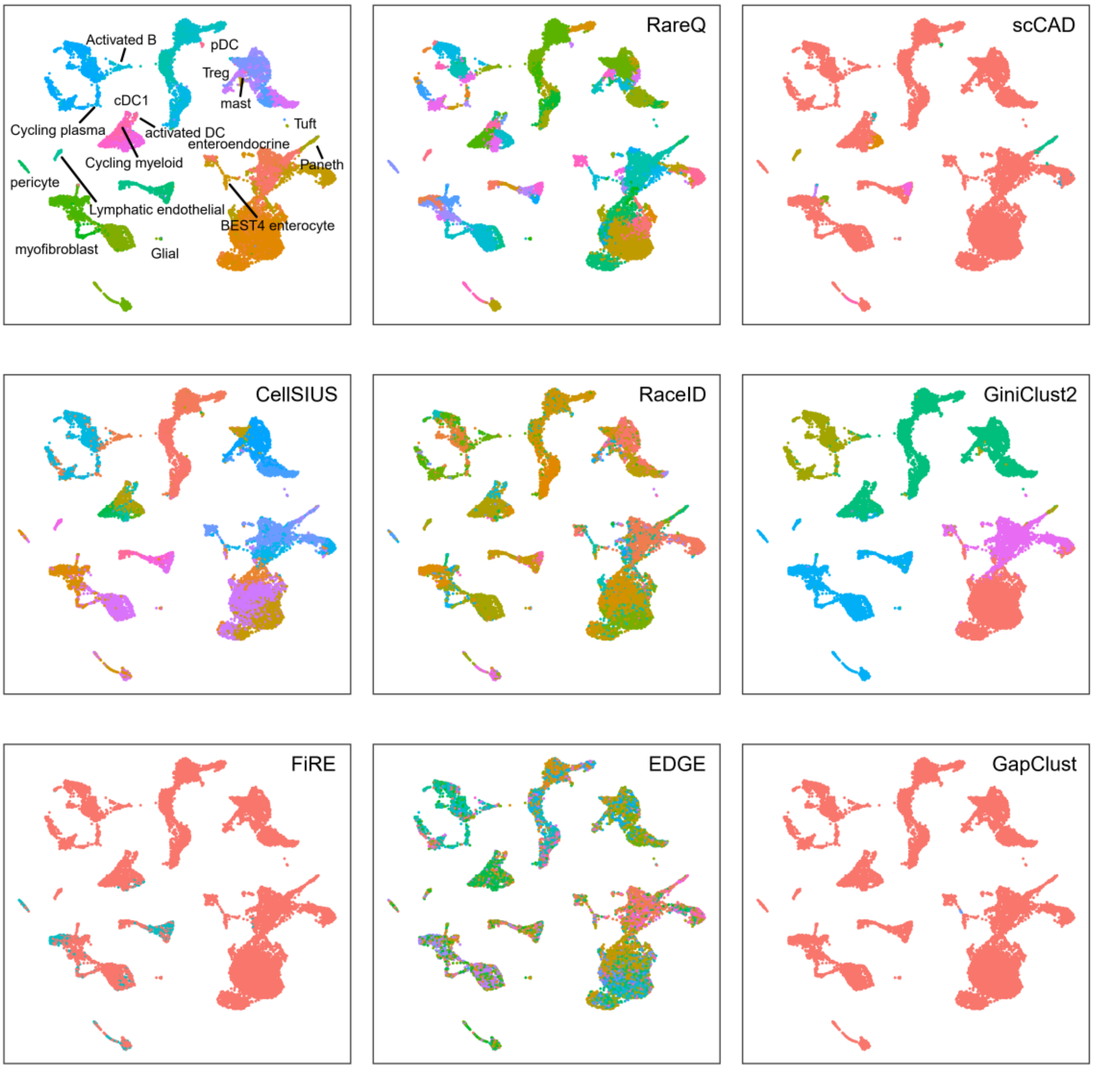

Supplementary Fig. 13 The UMAP 2D embedding of the cells in the Pediatric_gut dataset with rare cell types (< 1% population) highlighted. Rare cell clusters predicted by the specified methods are labeled with different colors. Source data are provided as a Source Data file.

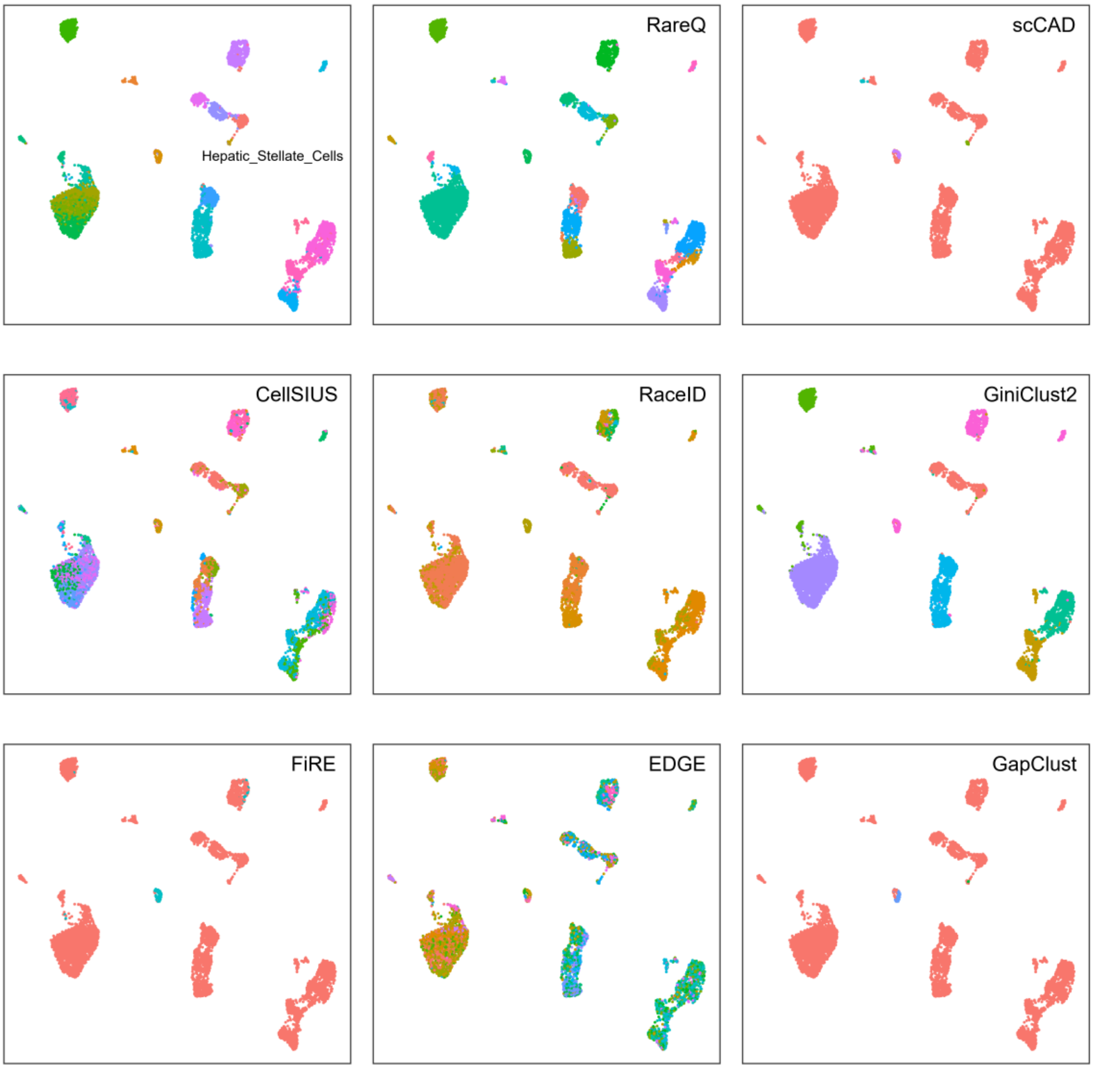

Supplementary Fig. 14 The UMAP 2D embedding of the cells in the MacParland dataset with rare cell types (< 1% population) highlighted. Rare cell clusters predicted by the specified methods are labeled with different colors. Source data are provided as a Source Data file.

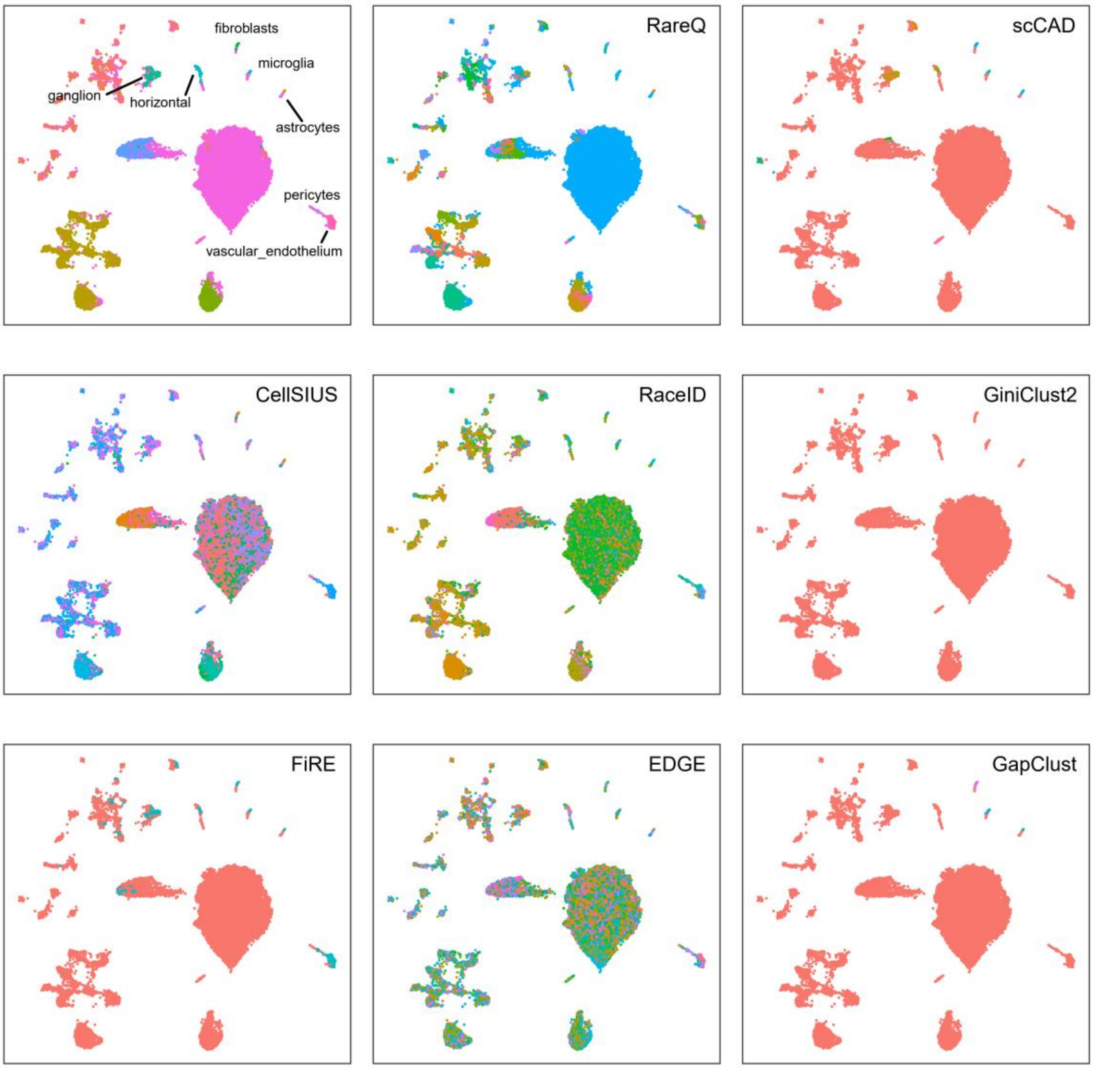

Supplementary Fig. 15 The UMAP 2D embedding of the cells in the Macosko dataset with rare cell types (< 1% population) highlighted. Rare cell clusters predicted by the specified methods are labeled with different colors. Source data are provided as a Source Data file.

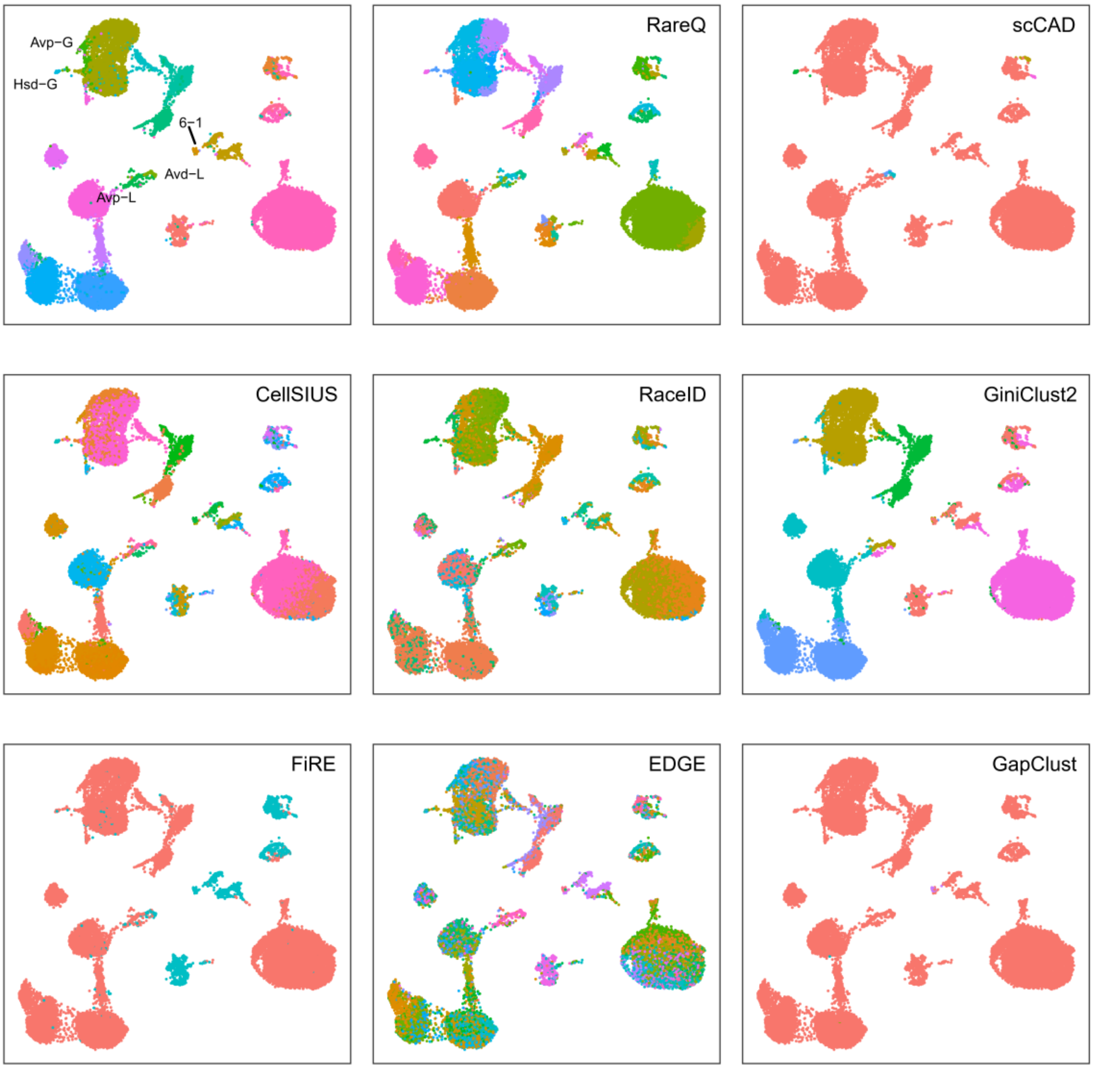

Supplementary Fig. 16 The UMAP 2D embedding of the cells in the Mammary dataset with rare cell types (< 1% population) highlighted. Rare cell clusters predicted by the specified methods are labeled with different colors. Source data are provided as a Source Data file.

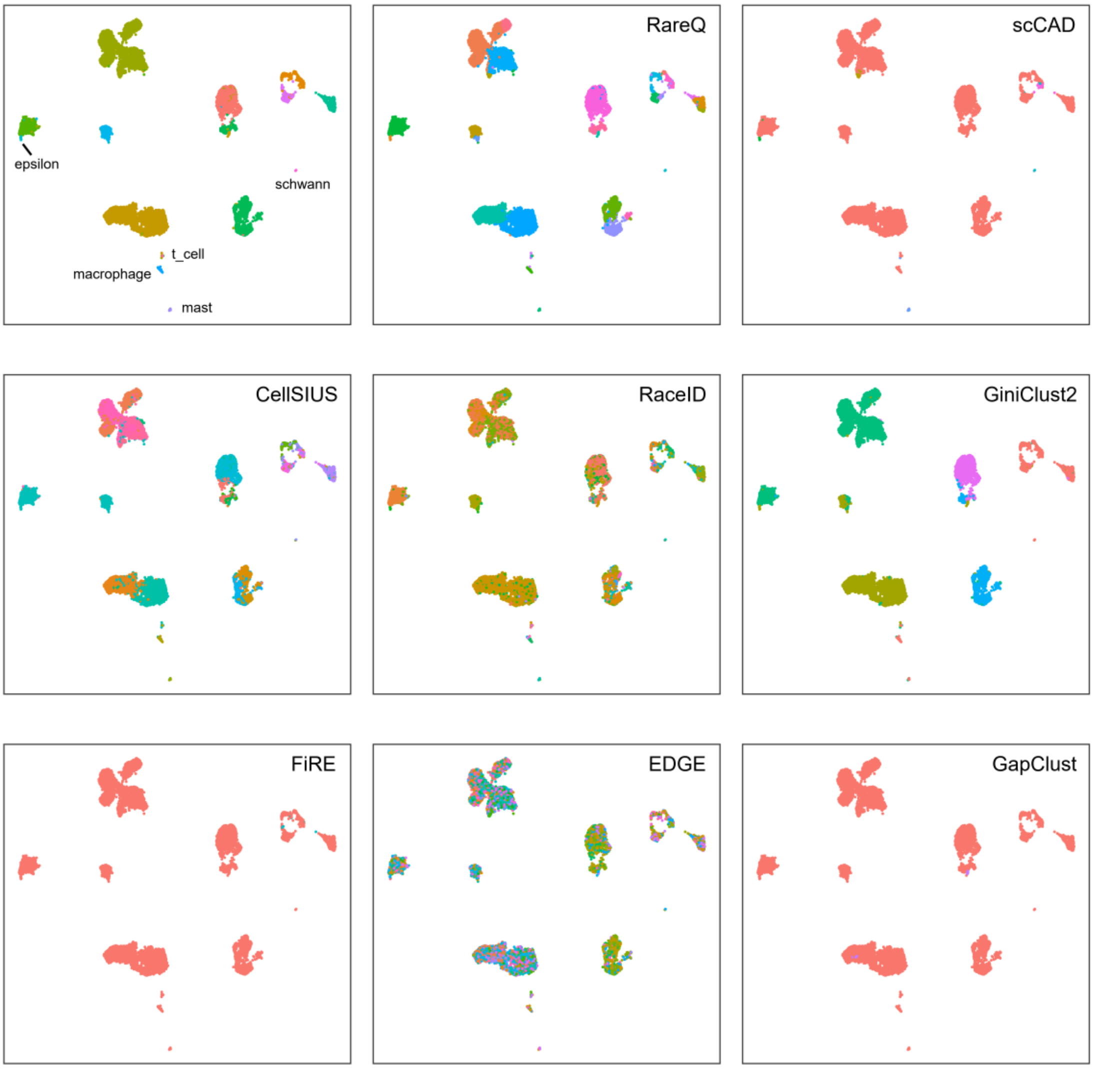

Supplementary Fig. 17 The UMAP 2D embedding of the cells in the Pancreas dataset with rare cell types (< 1% population) highlighted. Rare cell clusters predicted by the specified methods are labeled with different colors. Source data are provided as a Source Data file.

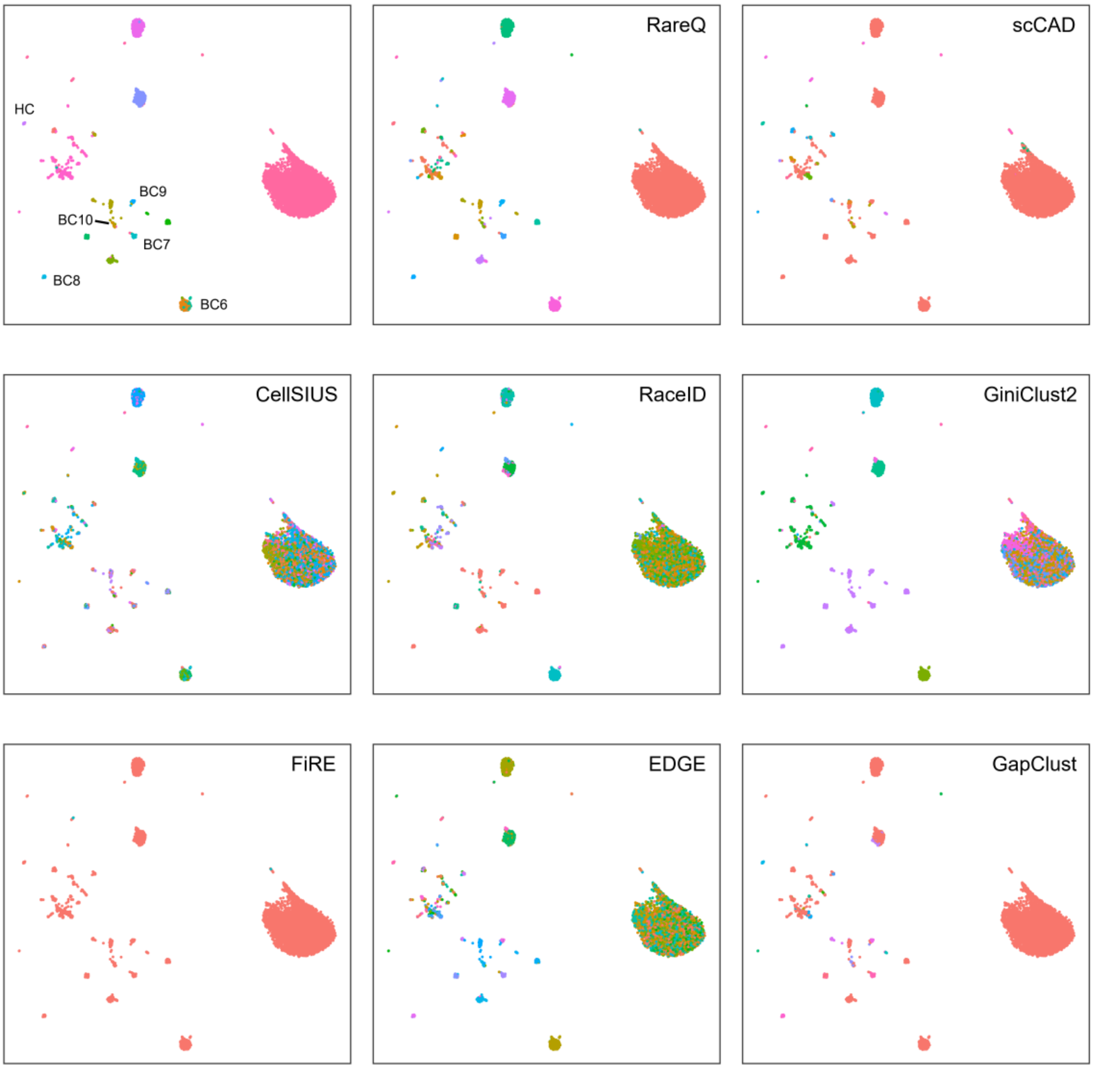

Supplementary Fig. 18 The UMAP 2D embedding of the cells in the Retina dataset with rare cell types (< 1% population) highlighted. Rare cell clusters predicted by the specified methods are labeled with different colors. Source data are provided as a Source Data file.

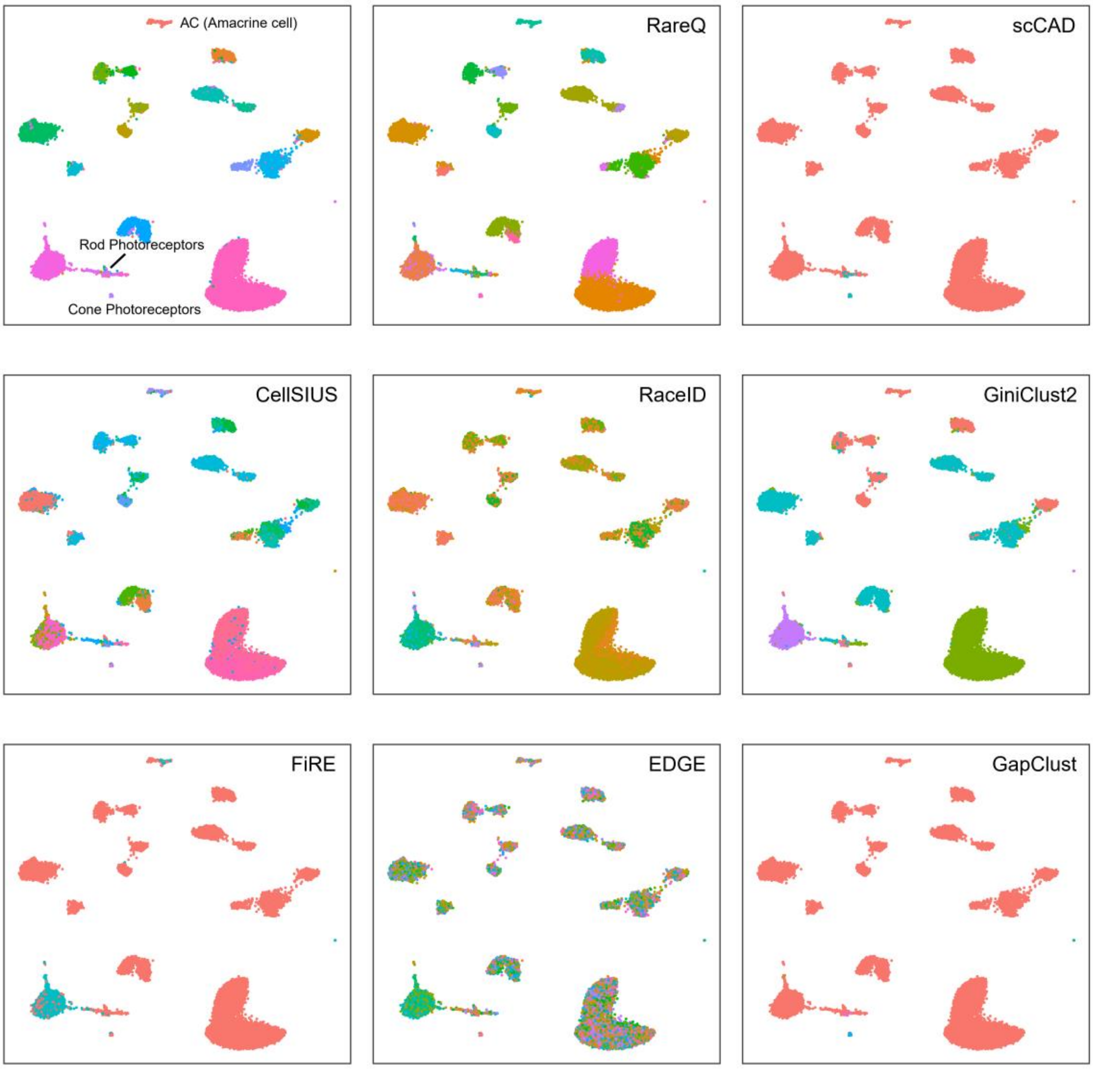

Supplementary Fig. 19 The UMAP 2D embedding of the cells in the Shekhar dataset with rare cell types (< 1% population) highlighted. Rare cell clusters predicted by the specified methods are labeled with different colors. Source data are provided as a Source Data file.

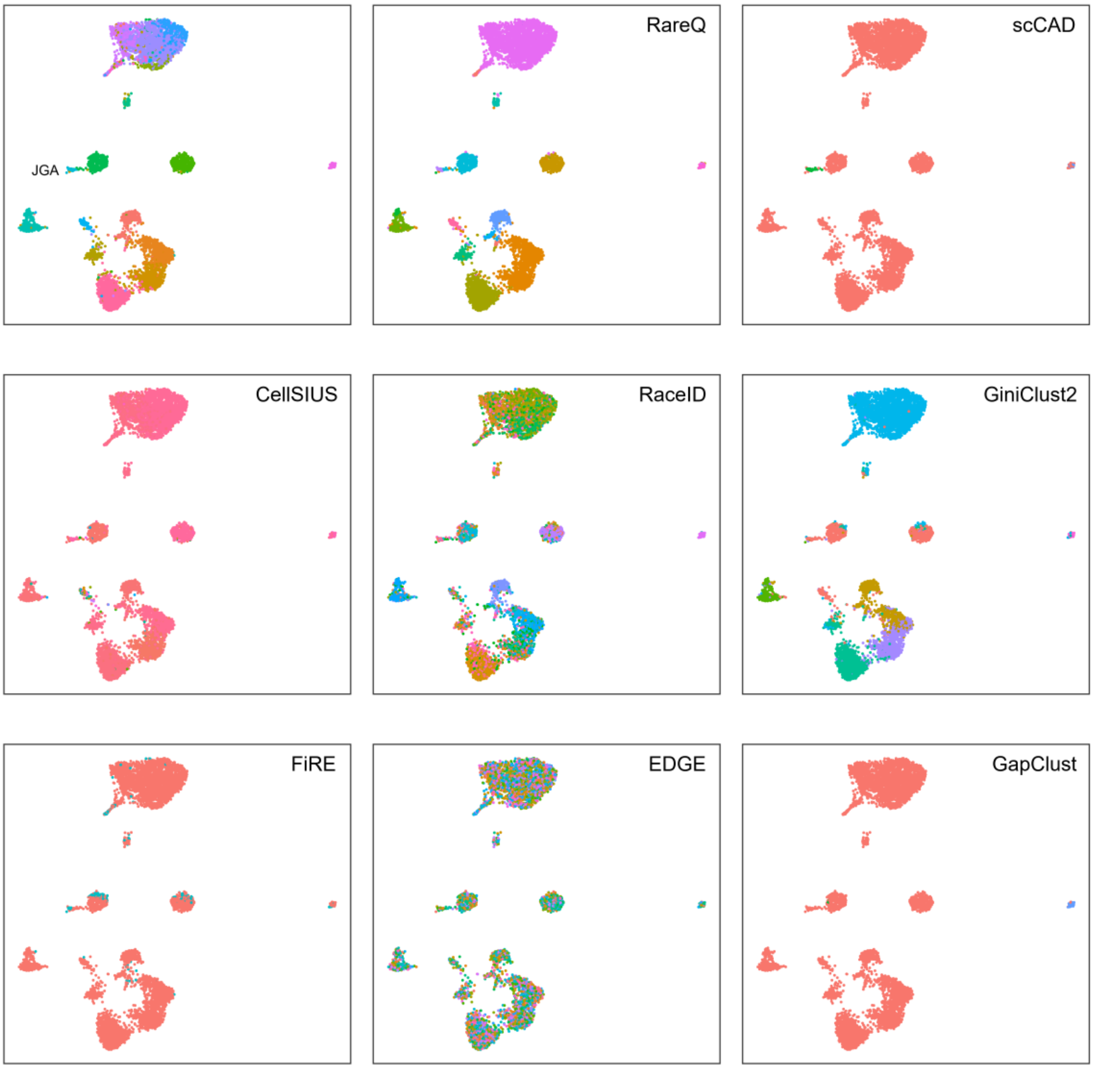

Supplementary Fig. 20 The UMAP 2D embedding of the cells in the UUOkidney dataset with rare cell types (< 1% population) highlighted. Rare cell clusters predicted by the specified methods are labeled with different colors. Source data are provided as a Source Data file.

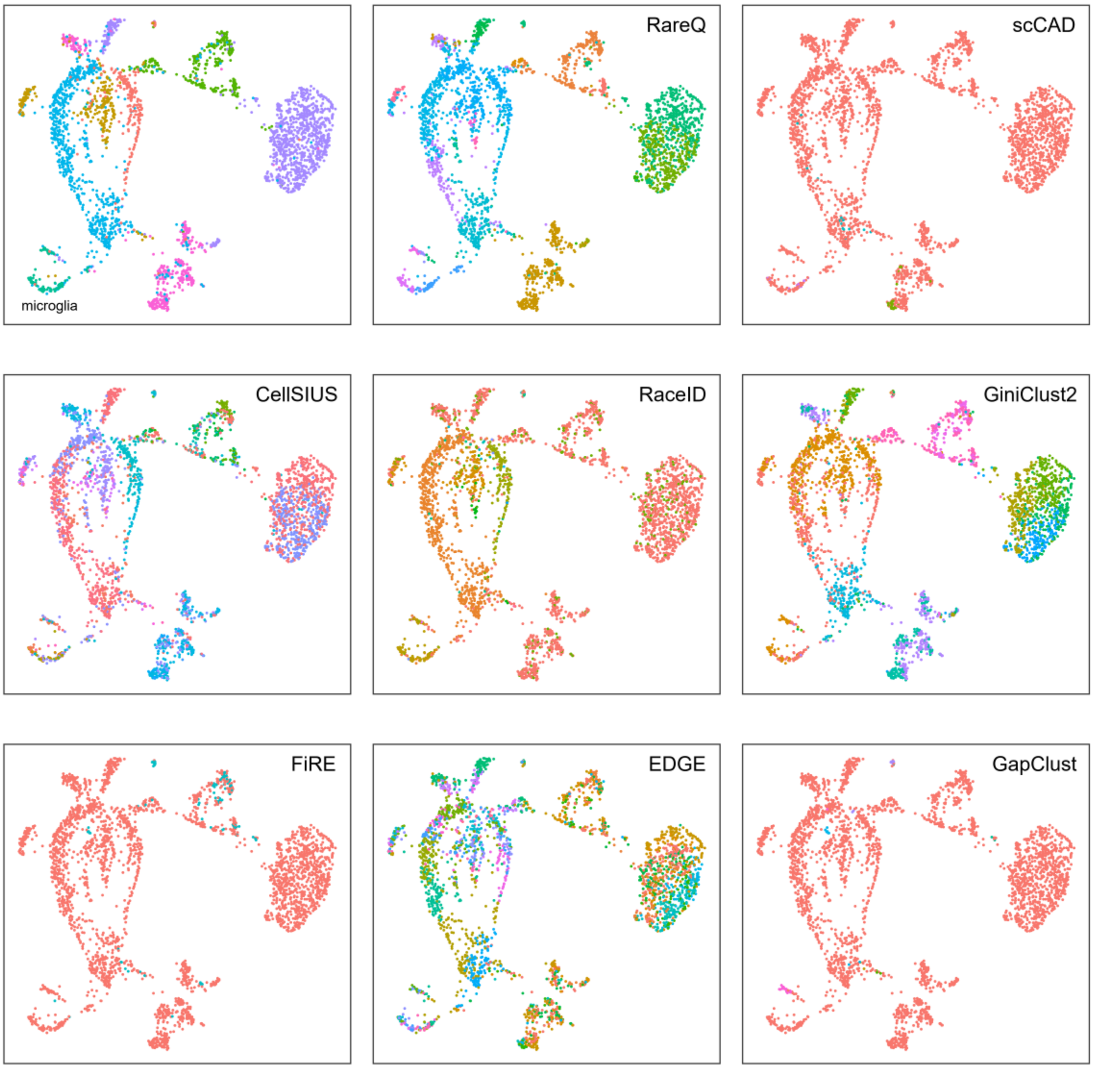

Supplementary Fig. 21 The UMAP 2D embedding of the cells in the Zelsel dataset with rare cell types (< 1% population) highlighted. Rare cell clusters predicted by the specified methods are labeled with different colors. Source data are provided as a Source Data file.

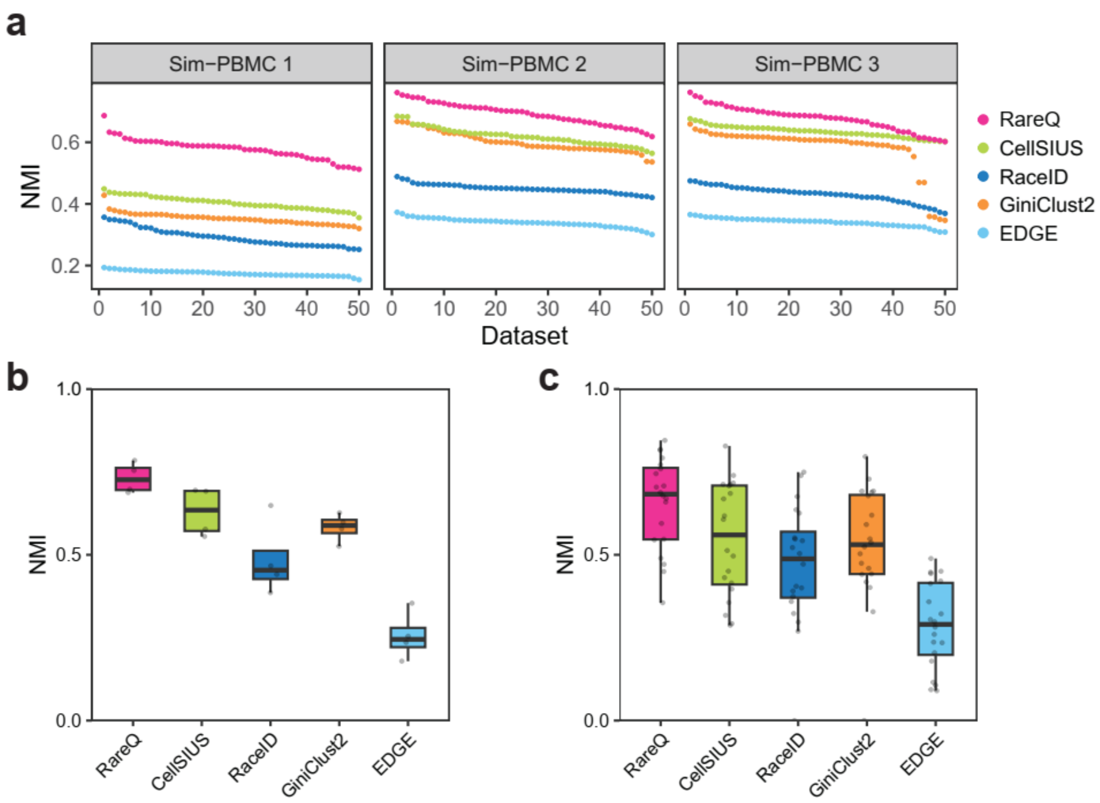

Supplementary Fig. 22 Benchmarking RareQ against existing methods on global clustering performance via NMI metrics in (**a**) 150 PBMC simulated scRNA-seq datasets, (**b**) PBMC-bench-1, 2, 3 and 4 scRNA-seq datasets and (**c**) 20 real scRNA-seq datasets from diverse systems. Source data are provided as a Source Data file.

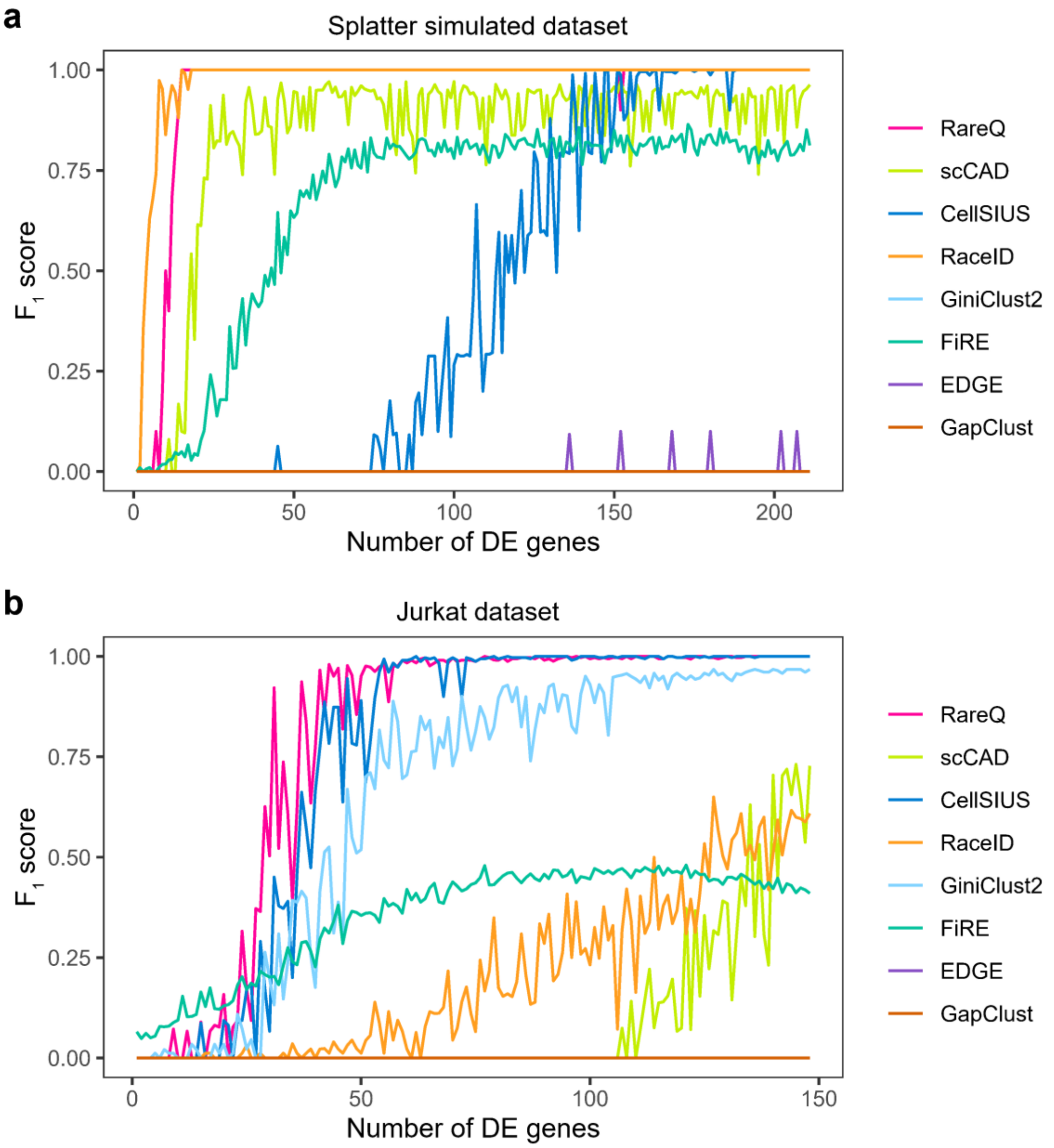

Supplementary Fig. 23 Comparison of the average F_1 score of different methods across iterations with different numbers of differentially expressed genes (DEGs) on the (**a**) artificial dataset and (**b**) Jurkat dataset. Source data are provided as a Source Data file.

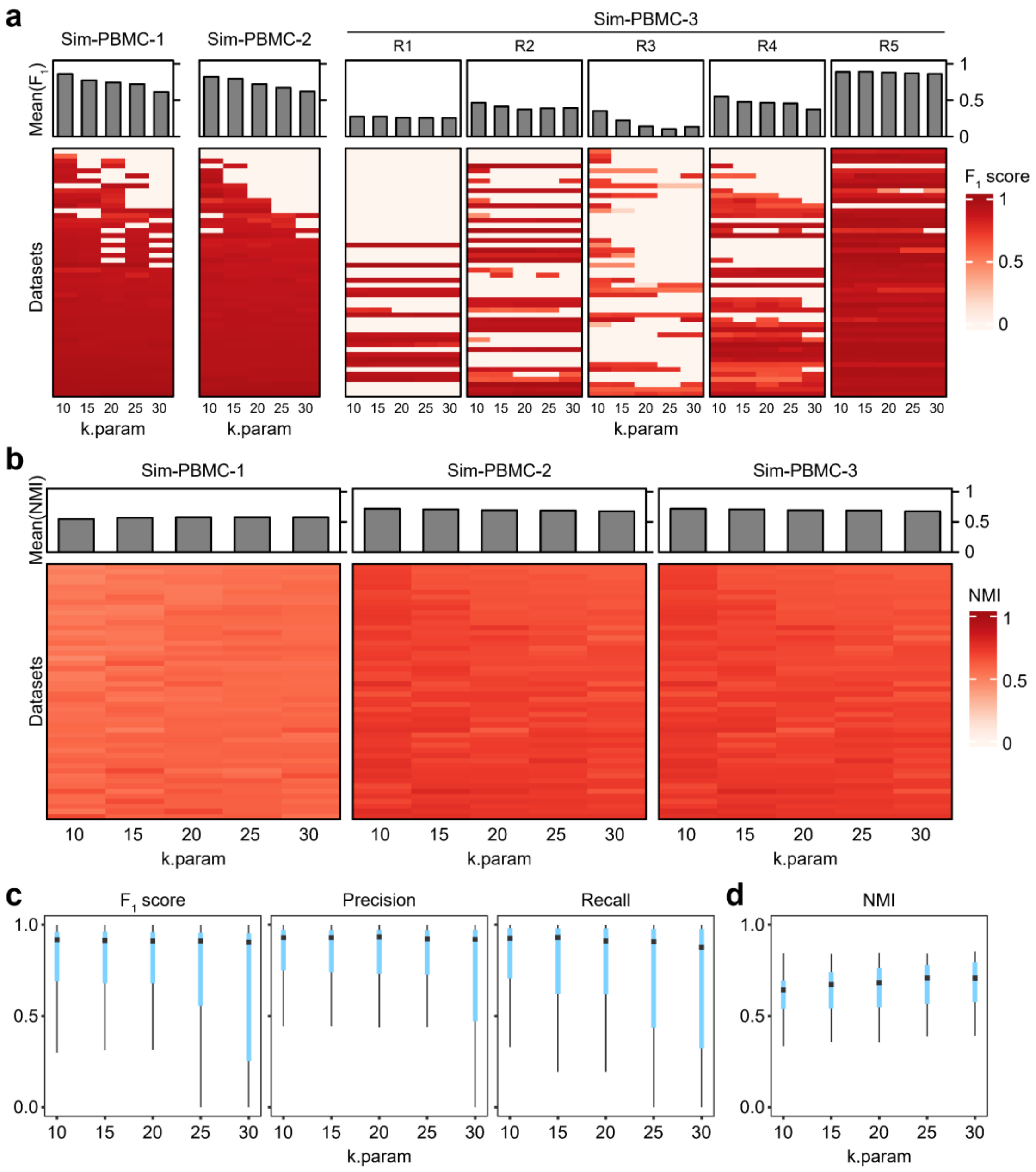

Supplementary Fig. 24 Evaluation of RareQ's sensitivity to the choice of k.param parameter of the preprocessing step from 10 to 30. Heatmaps showing the (**a**) F_1 scores in detecting rare cell types and (**b**) NMI metrics in global clustering of RareQ in PBMC simulated scRNA-seq datasets at varying k.param values. Box plots showing the (**c**) F_1 scores in detecting rare cell types and (**d**) NMI metrics in global clustering of RareQ in 20 real scRNA-seq datasets at varying k.param values. Source data are provided as a Source Data file.

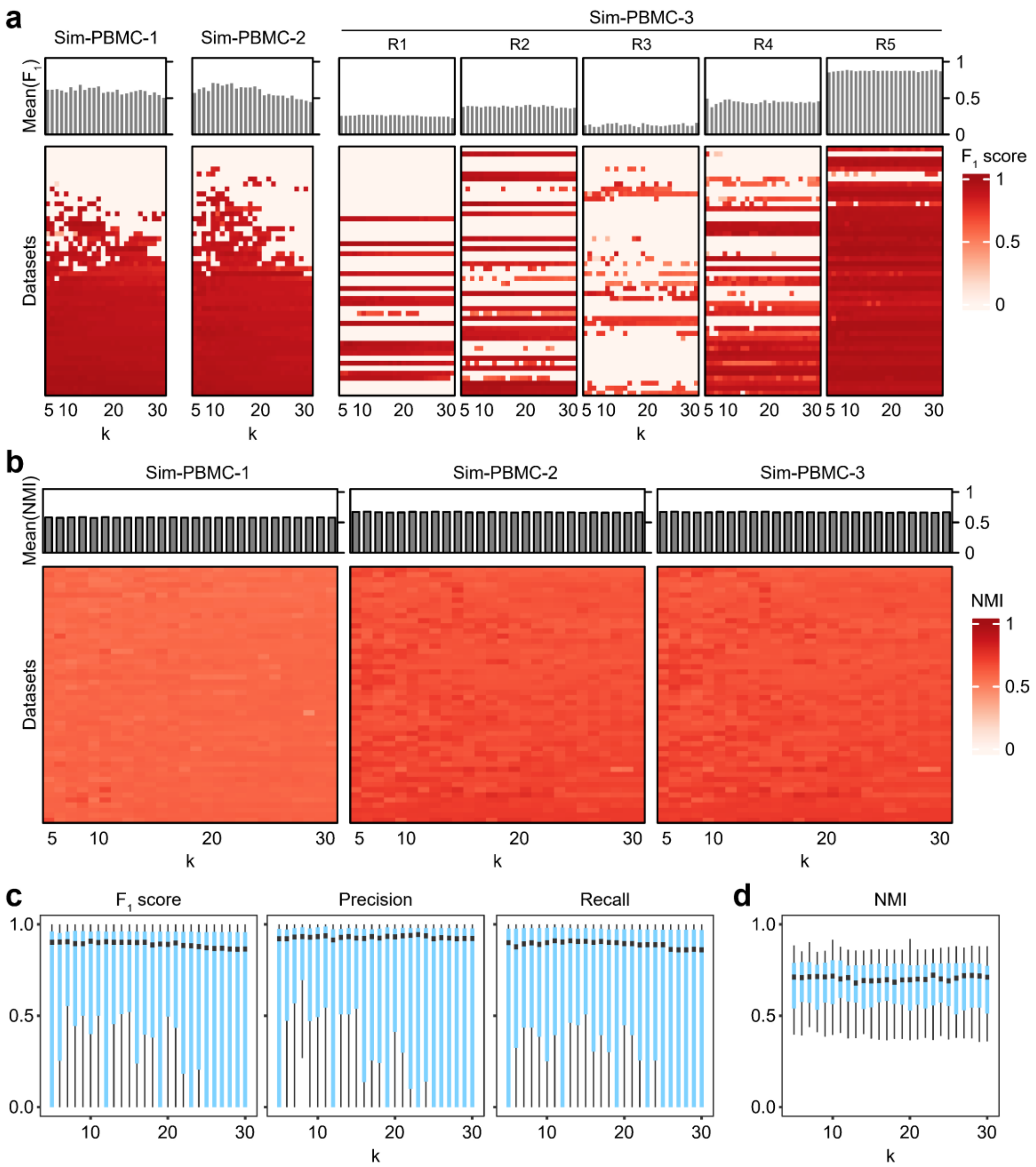

Supplementary Fig. 25 Evaluation of RareQ's sensitivity to the choice of *k* parameter from 5 to 30. Heatmaps showing the (**a**) F_1 scores in detecting rare cell types and (**b**) NMI metrics in global clustering of RareQ in PBMC simulated scRNA-seq datasets at varying *k* values. Box plots showing the (**c**) F_1 scores in detecting rare cell types and (**d**) NMI metrics in global clustering of RareQ in 20 real scRNA-seq datasets at varying *k* values. Source data are provided as a Source Data file.

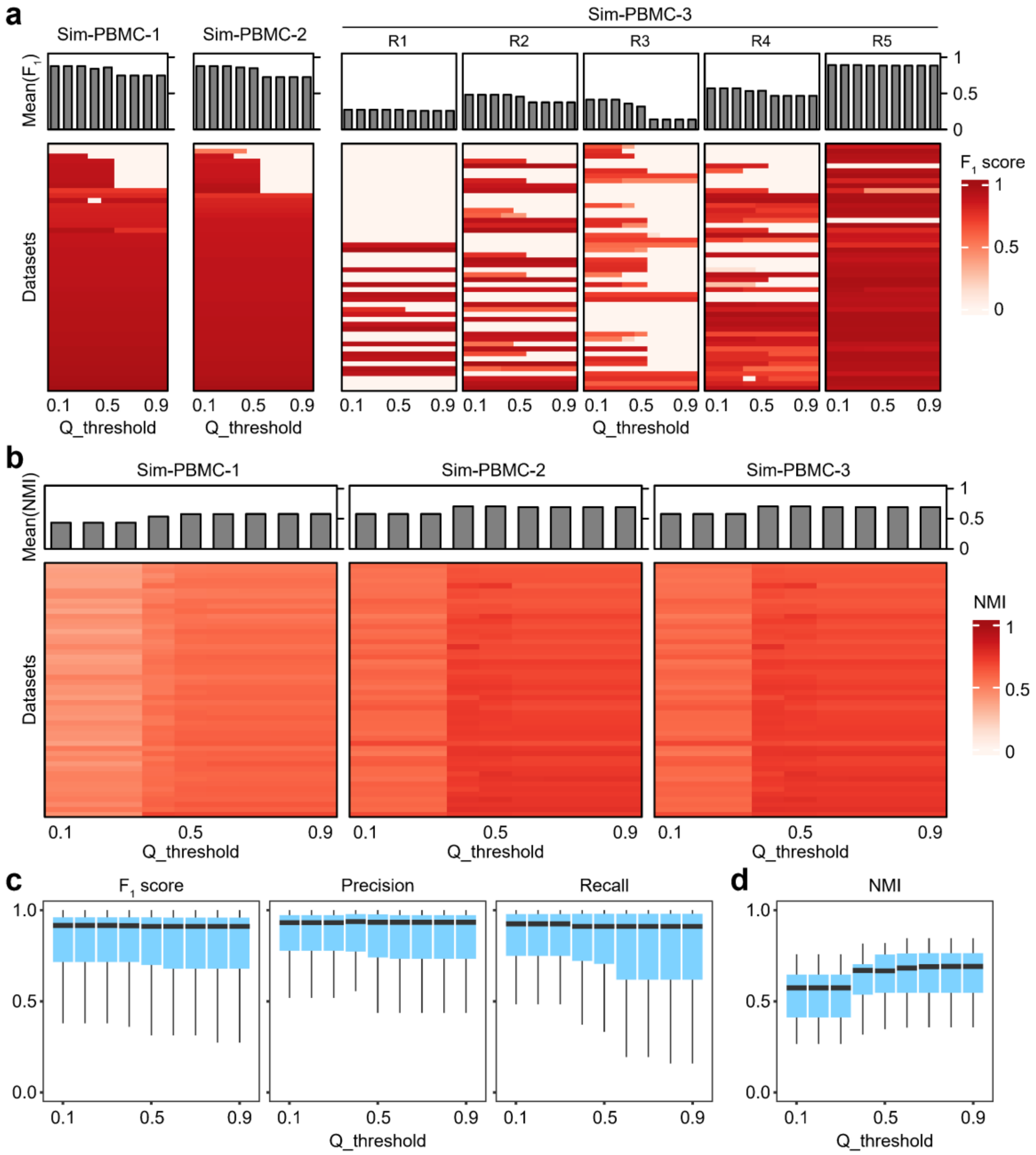

Supplementary Fig. 26 Evaluation of RareQ's sensitivity to the choice of the Q_threshold parameter from 0.1 to 0.9. Heatmaps showing the (**a**) F_1 scores in detecting rare cell types and (**b**) NMI metrics in global clustering of RareQ in PBMC simulated scRNA-seq datasets at varying Q_threshold values. Box plots showing the (**c**) F_1 scores in detecting rare cell types and (**d**) NMI metrics in global clustering of RareQ in 20 real scRNA-seq datasets at varying Q_threshold values. Source data are provided as a Source Data file.

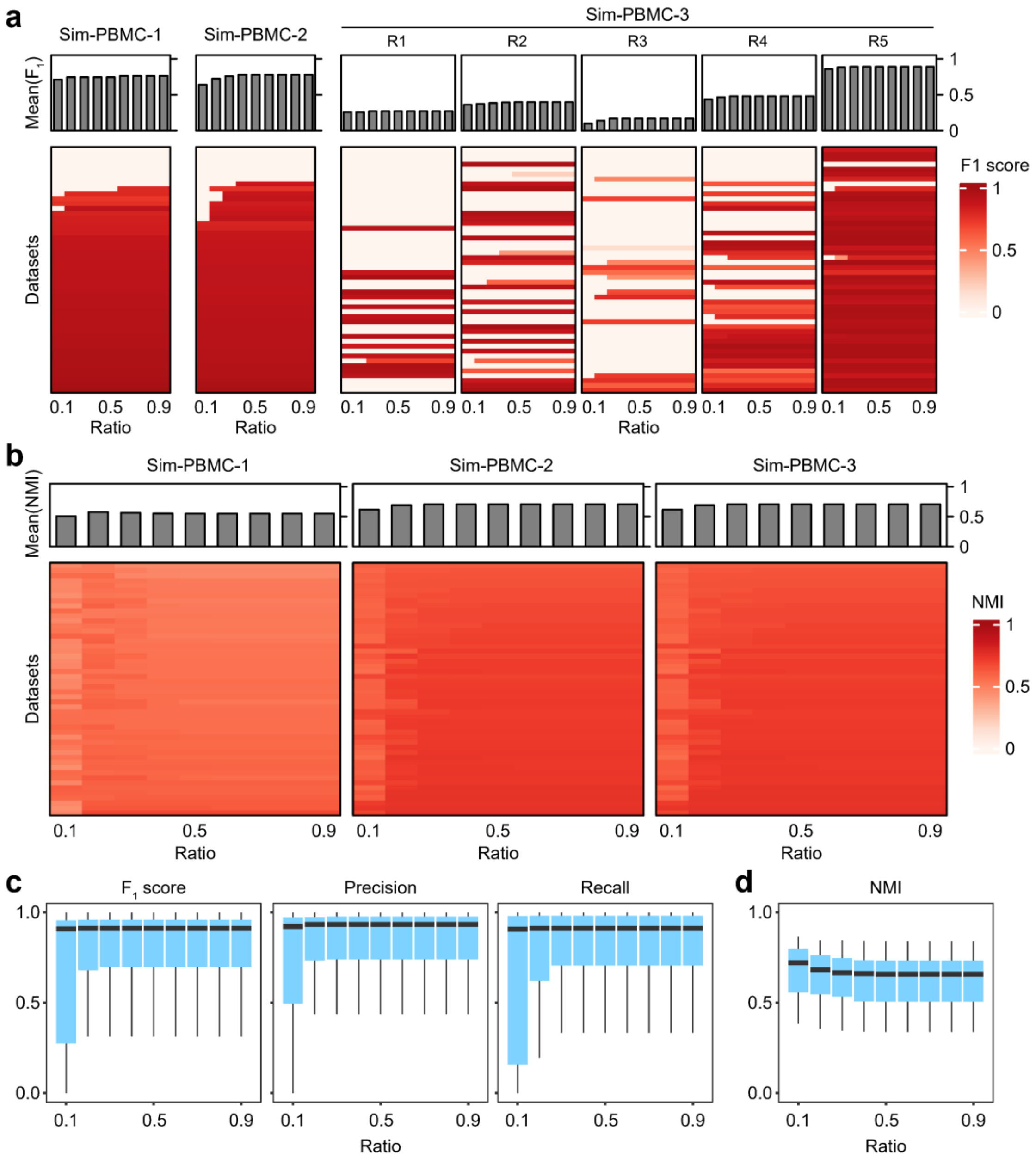

Supplementary Fig. 27 Evaluation of RareQ's sensitivity to the choice of ratio parameter from 0.1 to 0.9. Heatmaps showing the (**a**) F_1 scores in detecting rare cell types and (**b**) NMI metrics in global clustering of RareQ in PBMC simulated scRNA-seq datasets at varying ratio values. Box plots showing the (**c**) F_1 scores in detecting rare cell types and (**d**) NMI metrics in global clustering of RareQ in 20 real scRNA-seq datasets at varying ratio values. Source data are provided as a Source Data file.

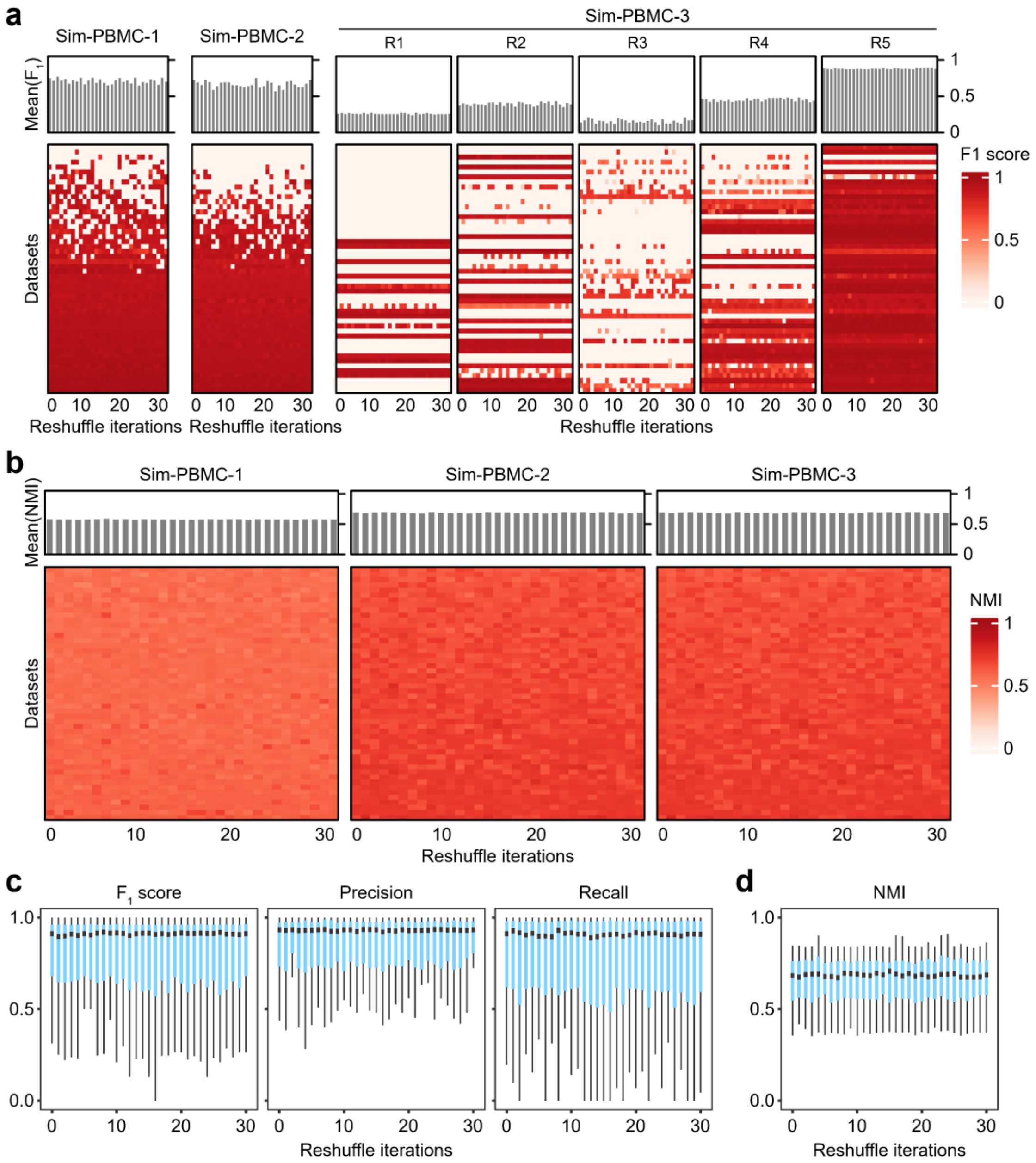

Supplementary Fig. 28 Evaluation of RareQ's sensitivity to the reshuffling of cell indices 30 times. Heatmaps showing the (**a**) F_1 scores in detecting rare cell types and (**b**) NMI metrics in global clustering of RareQ in PBMC simulated scRNA-seq datasets in different reshuffle iterations. Box plots showing the (**c**) F_1 scores in detecting rare cell types and (**d**) NMI metrics in global clustering of RareQ in 20 real scRNA-seq datasets in different reshuffle iterations. Source data are provided as a Source Data file.

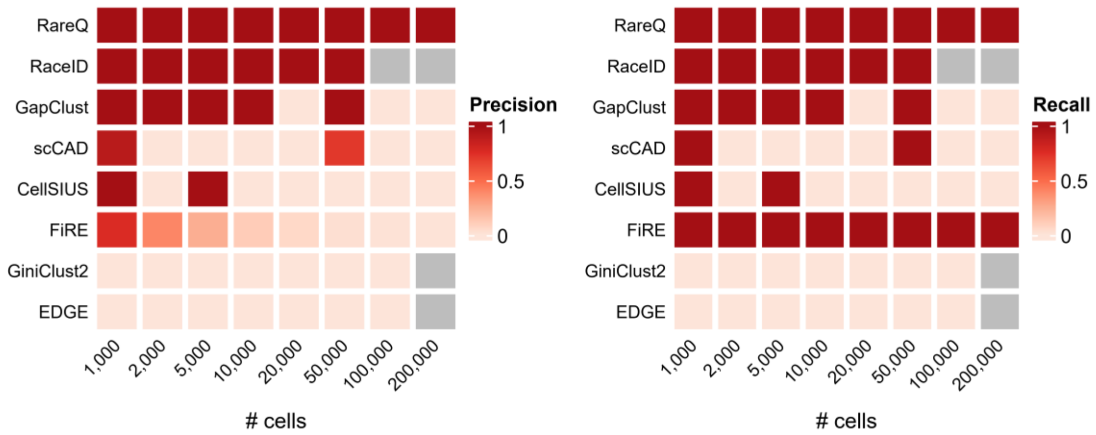

Supplementary Fig. 29 Benchmarking RareQ against existing methods in identifying the rare cell type with only 10 cells on the eight simulation scRNA-seq datasets in terms of Precision (left) and Recall (right). Source data are provided as a Source Data file.

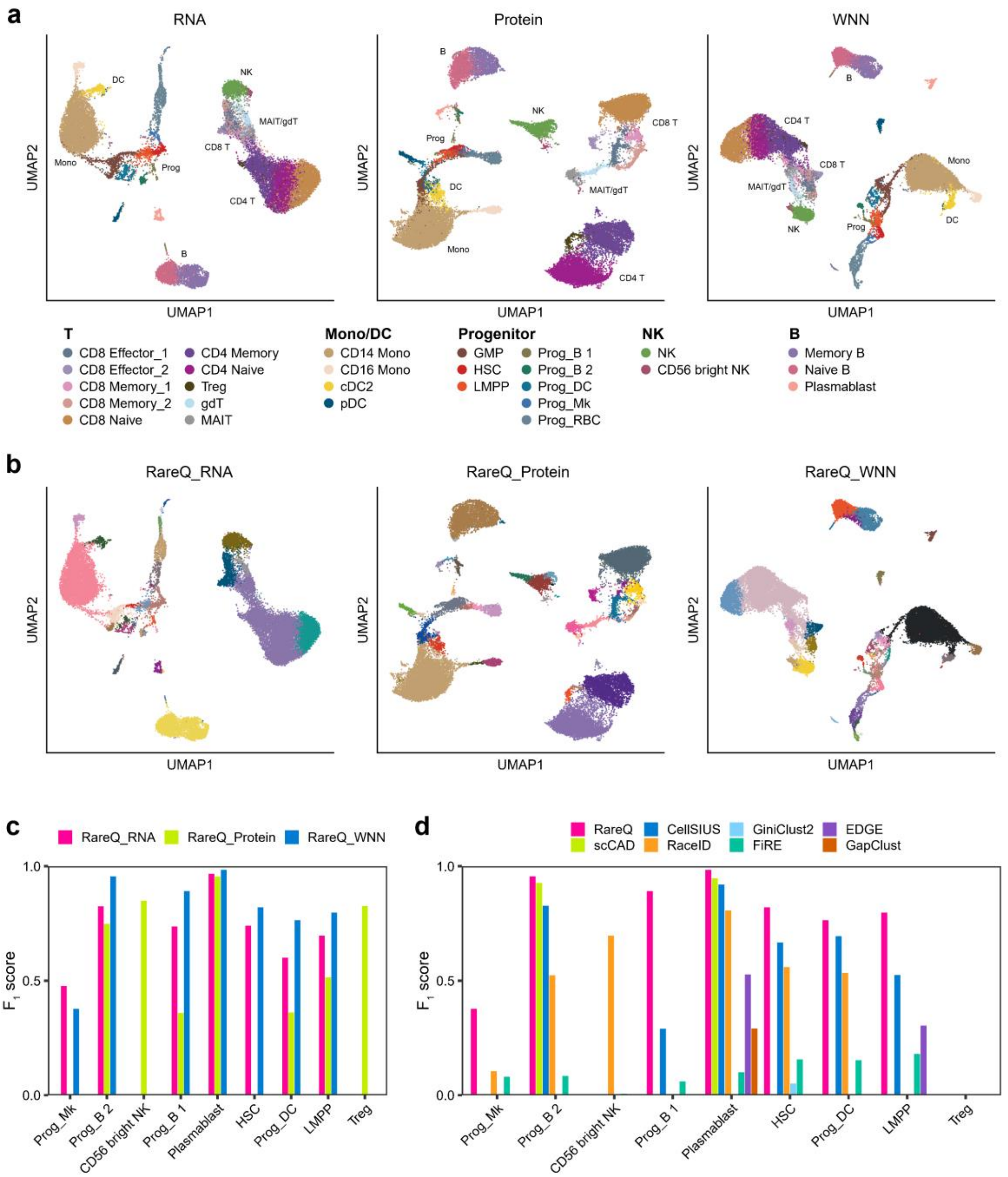

Supplementary Fig. 30 Benchmarking RareQ on WNN-integrated CITE-seq data. **a**, UMAP visualizations of the CITE-seq dataset of human bone marrow mononuclear cells using RNA, protein, or WNN-integration analysis. Cells are colored by annotations. **b**, UMAPs visualizing the predictions of RareQ using RNA, protein and WNN-integrated data. Cells are colored by predicted clusters. **c**, Comparative results of RareQ in rare cell (<1% population) detection when using RNA, protein and WNN-integrated data via F_1 scores, respectively. **d**, Comparative results of RareQ against existing methods in rare cell (<1% population) detection when using WNN-integrated data or concatenated counts via F_1 scores. Source data are provided as a Source Data file.

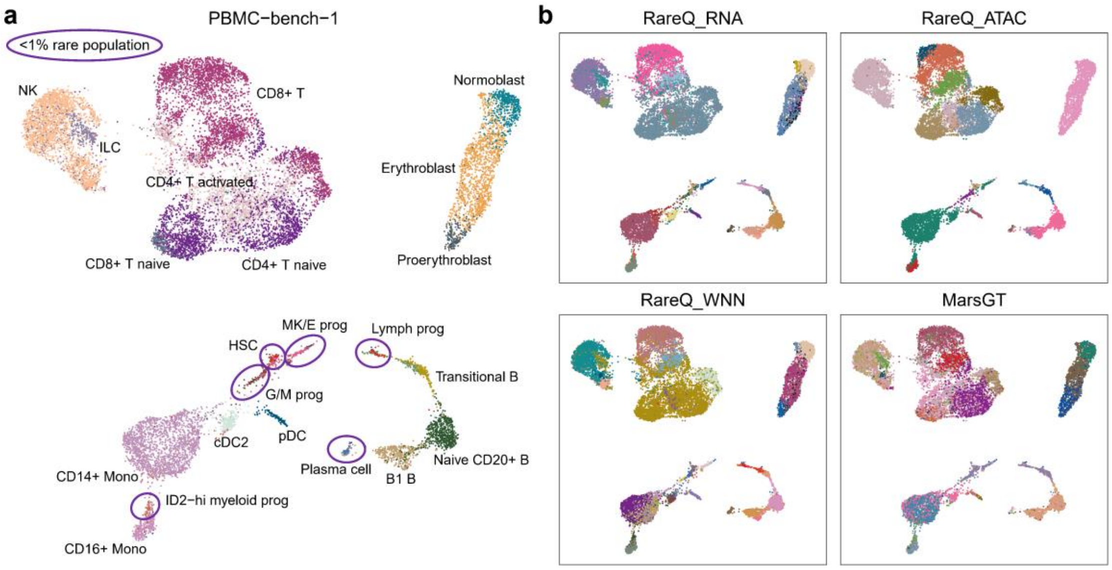

Supplementary Fig. 31 UMAPs on ATAC visualizing (**a**) rare cell types (< 1% total cells, highlighted in purple circles) and (**b**) predicted cell clusters by specified tools in the PBMC-bench-1 dataset. Source data are provided as a Source Data file.

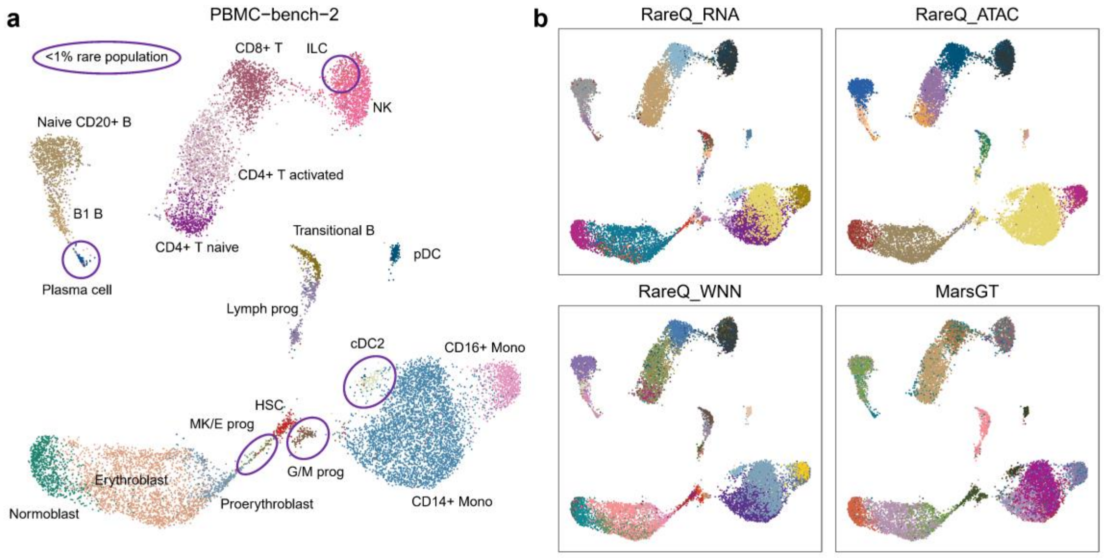

Supplementary Fig. 32 UMAPs on ATAC visualizing (**a**) rare cell types (< 1% total cells, highlighted in purple circles) and (**b**) predicted cell clusters by specified tools in the PBMC-bench-2 dataset. Source data are provided as a Source Data file.

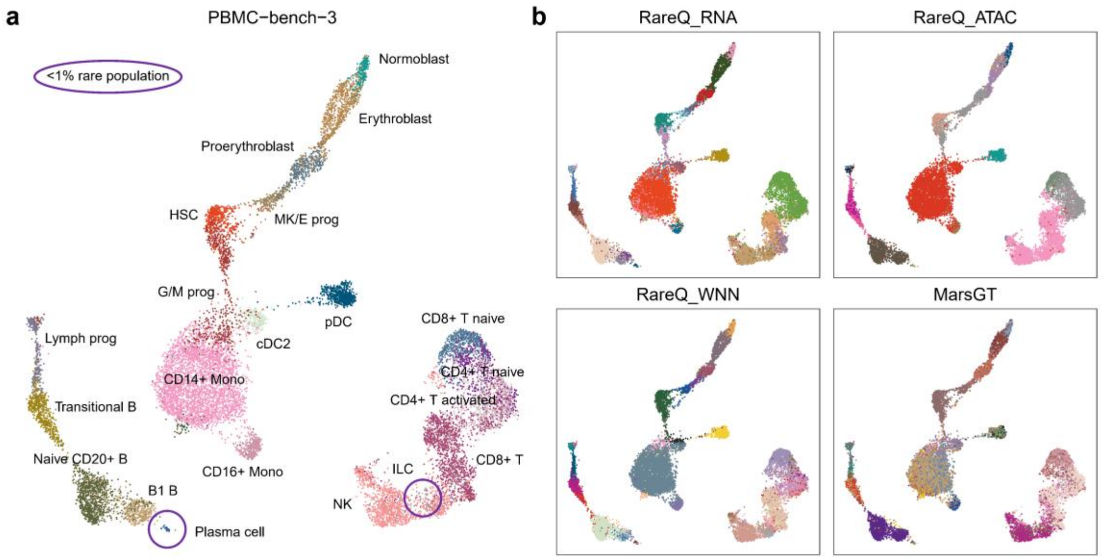

Supplementary Fig. 33 UMAPs on ATAC visualizing (**a**) rare cell types (< 1% total cells, highlighted in purple circles) and (**b**) predicted cell clusters by specified tools in the PBMC-bench-3 dataset. Source data are provided as a Source Data file.

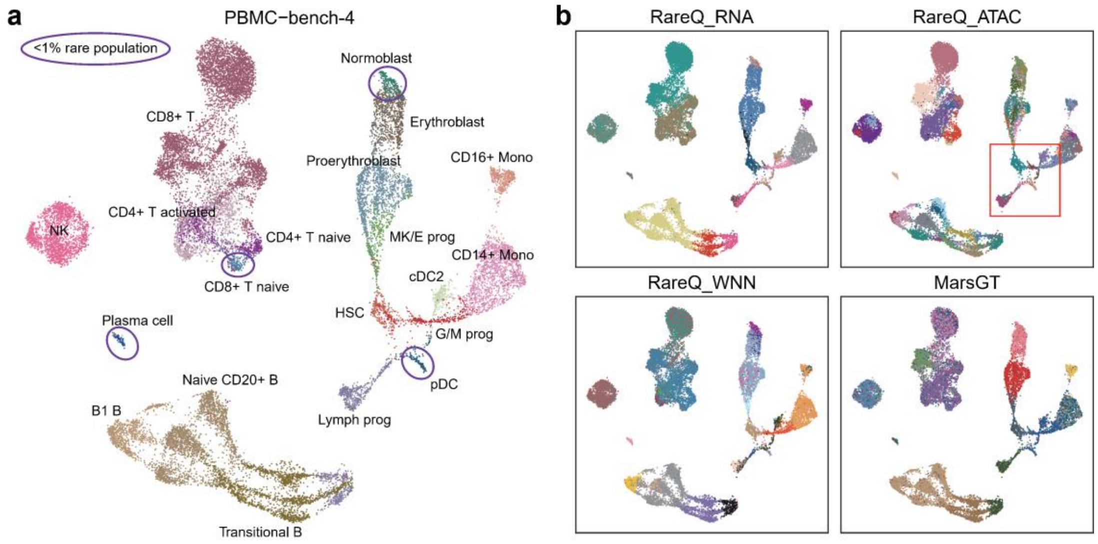

Supplementary Fig. 34 UMAPs on ATAC visualizing (**a**) rare cell types (< 1% total cells, highlighted in purple circles) and (**b**) predicted cell clusters by specified tools in the PBMC-bench-4 dataset. Source data are provided as a Source Data file.

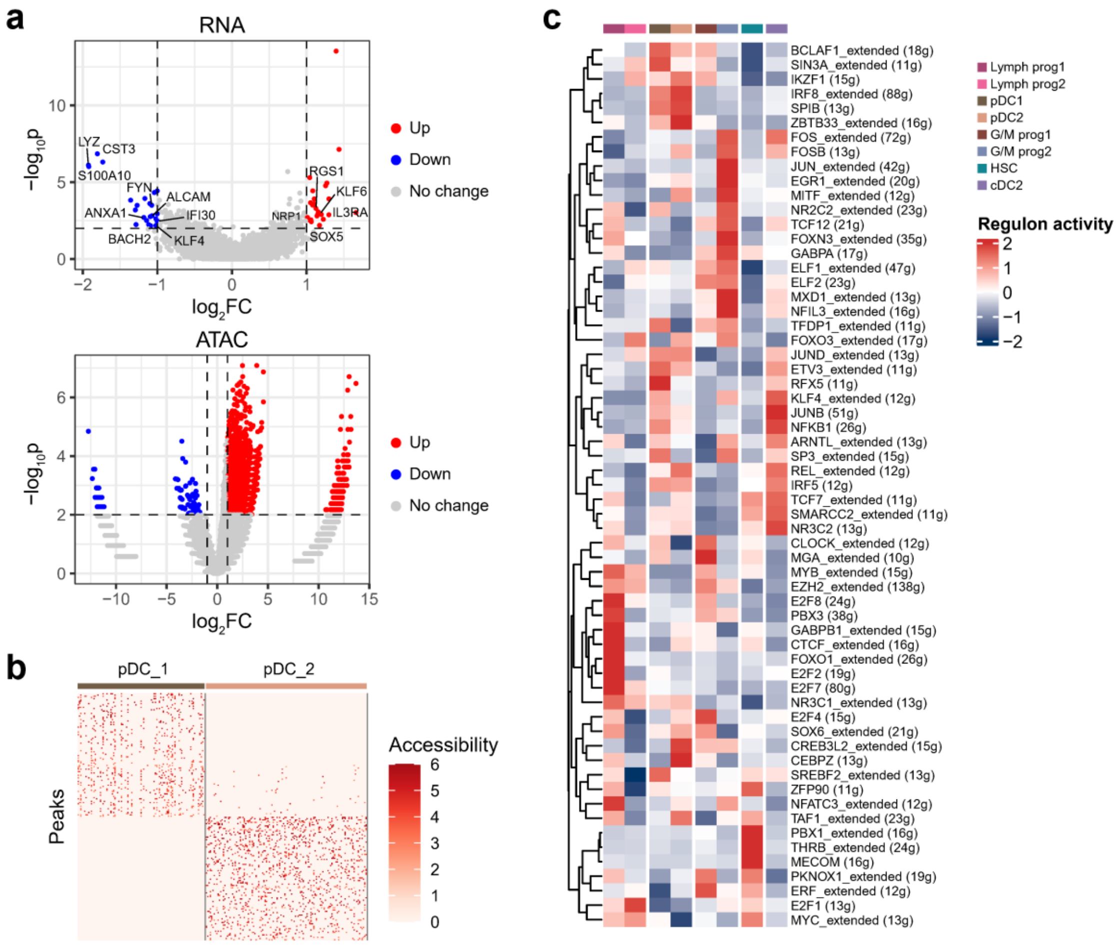

Supplementary Fig. 35 Molecular comparison of progenitor cell types in the PBMC-bench-4 dataset. **a**, Volcano plots of differentially expressed genes (RNA) and peaks (ATAC) between pDC_2 and pDC_1 subtype. **b**, Heatmap showing the top 100 differential peaks between pDC_2 and pDC_1. **c**, Heatmap visualizing the regulon activities of progenitor cell types. Source data are provided as a Source Data file.

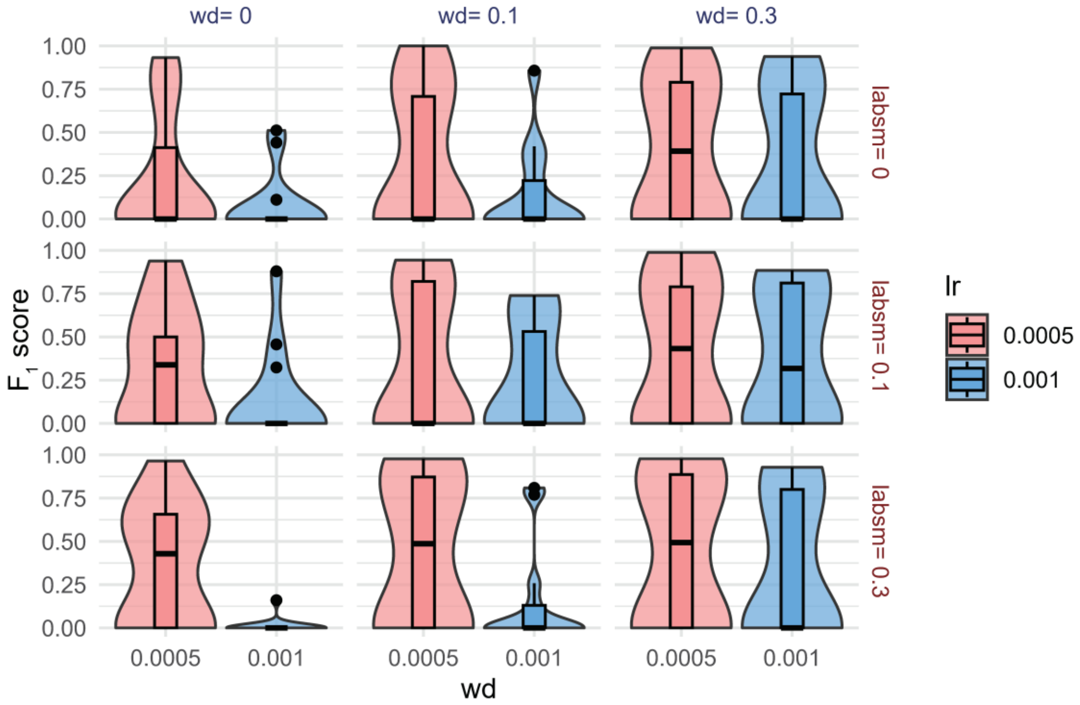

Supplementary Fig. 36 Hyperparameter tuning of MarsGT via F_1 score across different combinations of weight decay (wd), learning rate (lr), and label-smoothing (labsm) parameters in four multiome datasets. Source data are provided as a Source Data file.

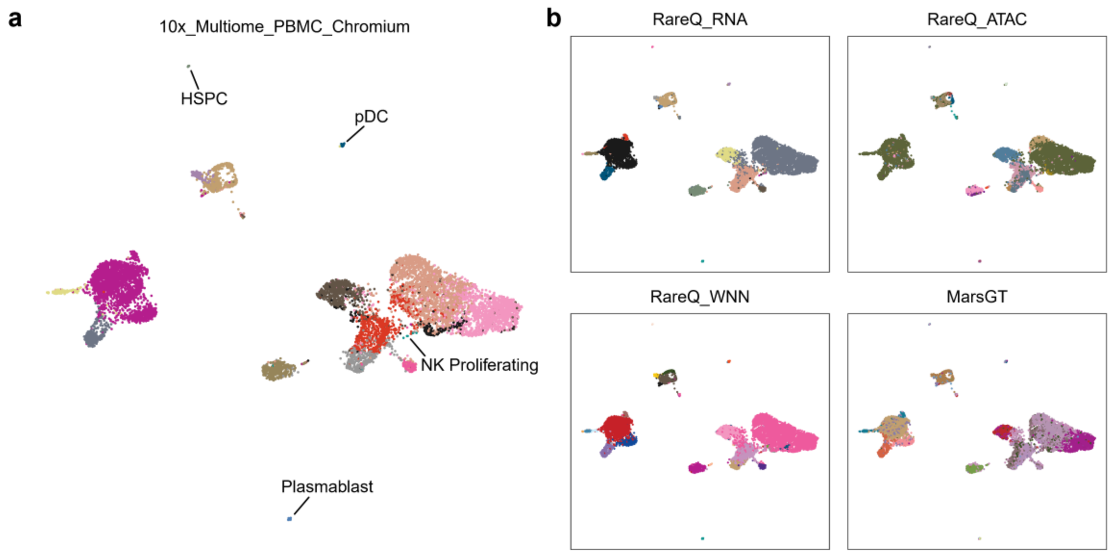

Supplementary Fig. 37 UMAPs on WNN integration visualizing (**a**) rare cell types (< 1% total cells, highlighted by labels) and (**b**) predicted cell clusters by specified tools in the 10x_Multiome_PBMC_Chromium dataset. Source data are provided as a Source Data file.

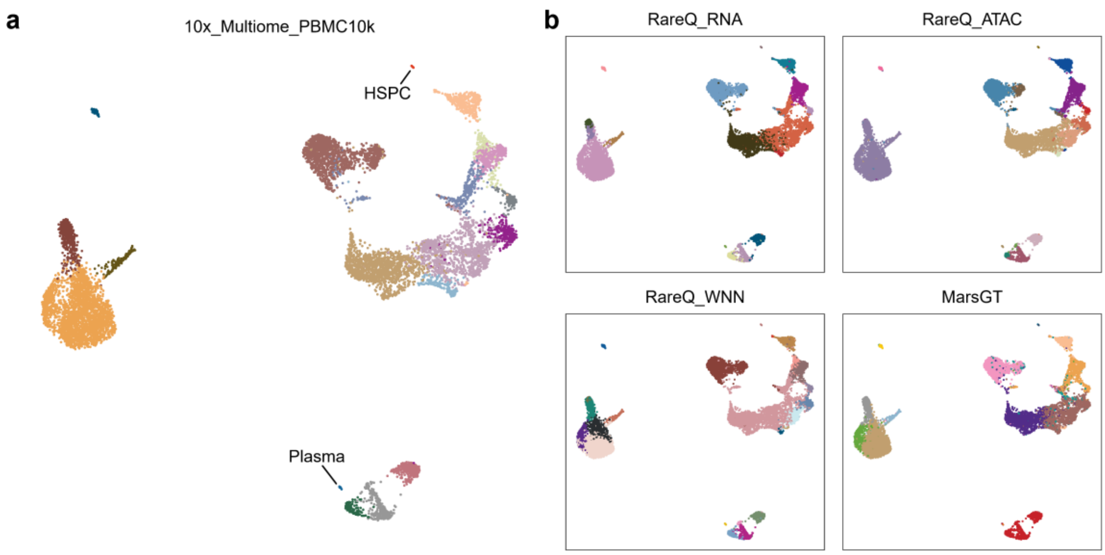

Supplementary Fig. 38 UMAPs on WNN integration visualizing (**a**) rare cell types (< 1% total cells, highlighted by labels) and (**b**) predicted cell clusters by specified tools in the 10x_Multiome_PBMC10k dataset. Source data are provided as a Source Data file.

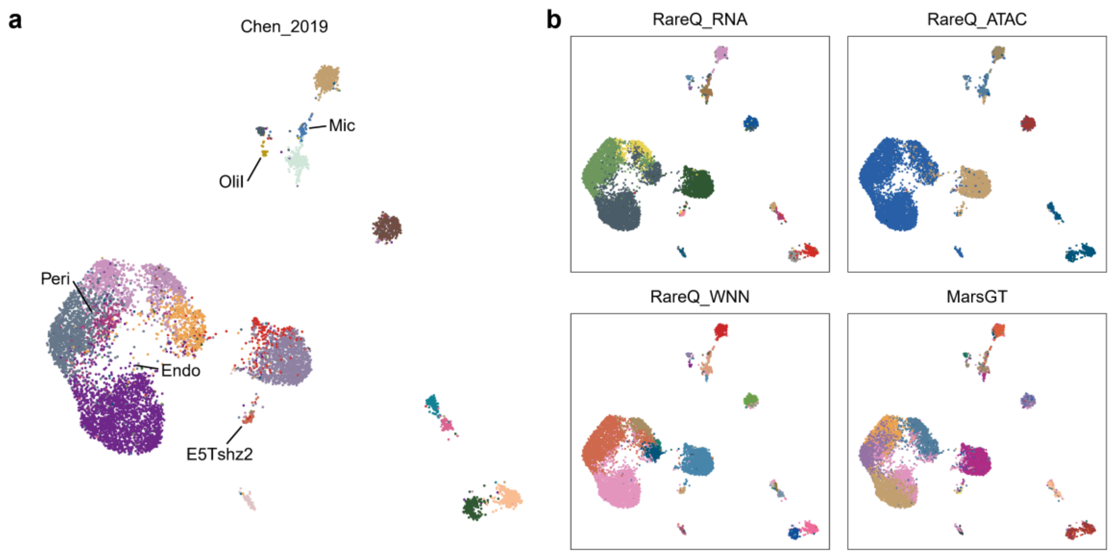

Supplementary Fig. 39 UMAPs on WNN integration visualizing (**a**) rare cell types (< 1% total cells, highlighted by labels) and (**b**) predicted cell clusters by specified tools in the Chen_2019 dataset. Source data are provided as a Source Data file.

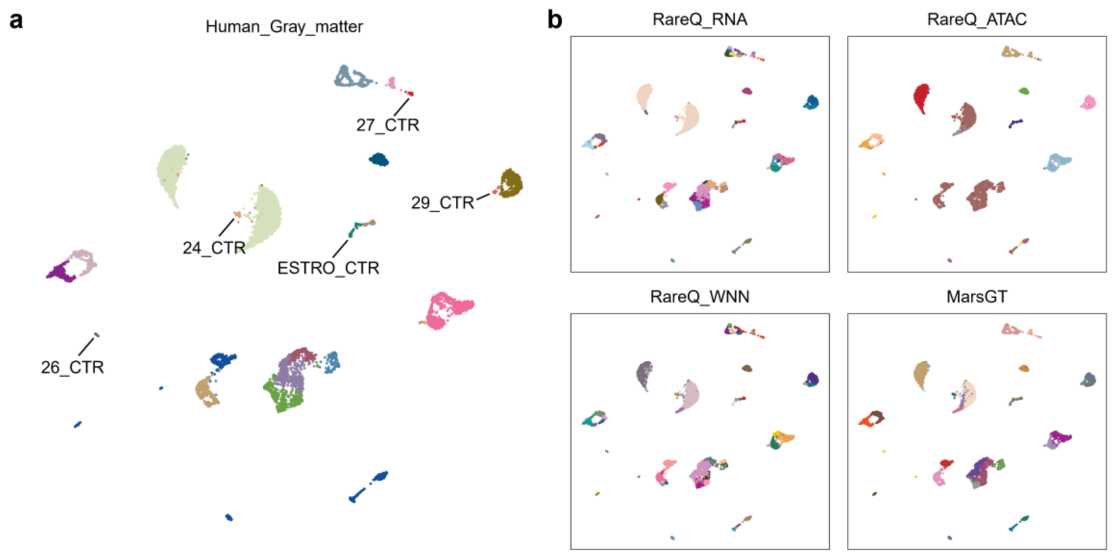

Supplementary Fig. 40 UMAPs on WNN integration visualizing (**a**) rare cell types (< 1% total cells, highlighted by labels) and (**b**) predicted cell clusters by specified tools in the Human_Gray_matter dataset. Source data are provided as a Source Data file.

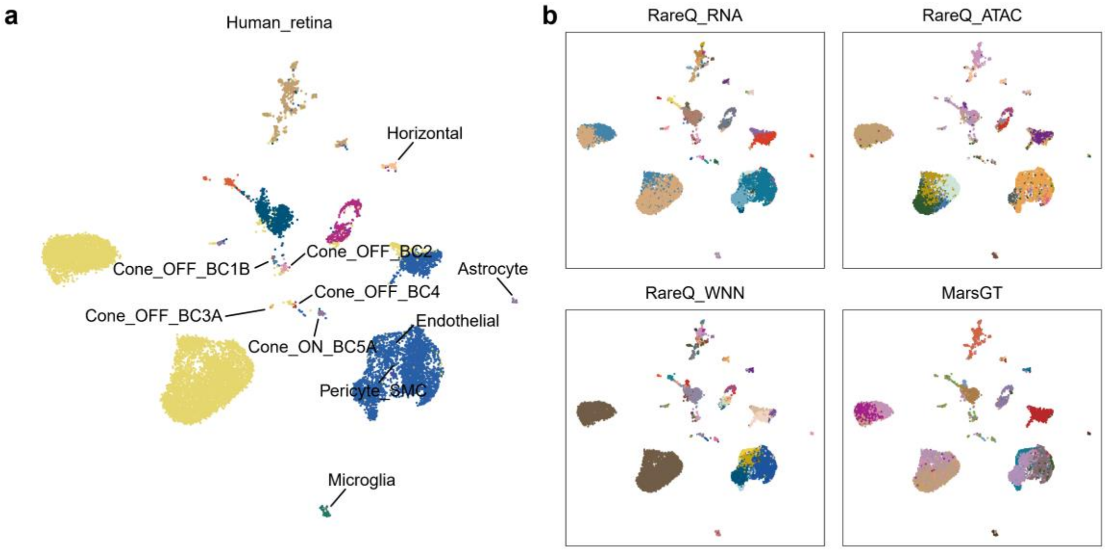

Supplementary Fig. 41 UMAPs on WNN integration visualizing (**a**) rare cell types (< 1% total cells, highlighted by labels) and (**b**) predicted cell clusters by specified tools in the Human_retina dataset. Source data are provided as a Source Data file.

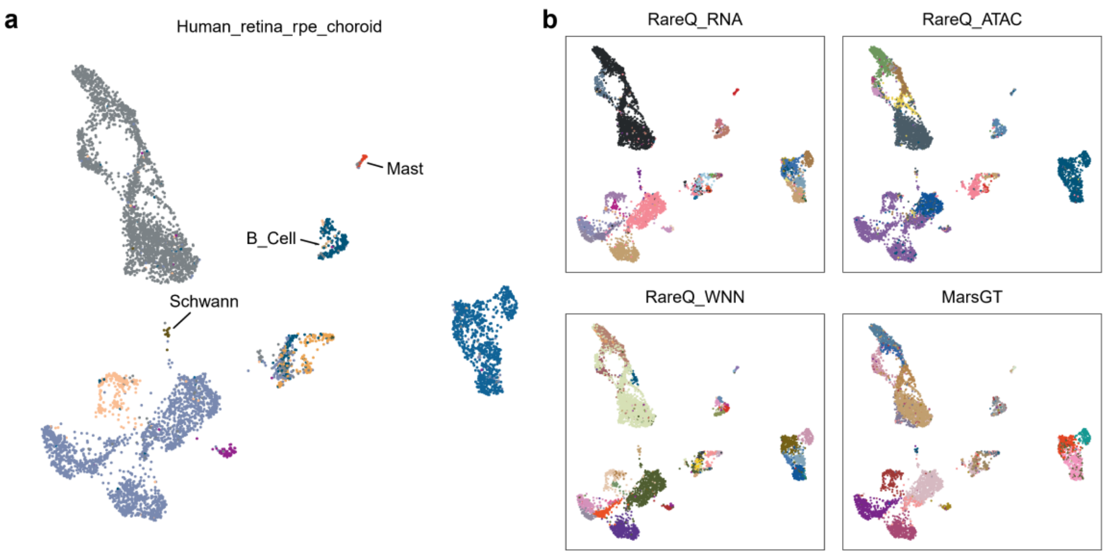

Supplementary Fig. 42 UMAPs on WNN integration visualizing (**a**) rare cell types (< 1% total cells, highlighted by labels) and (**b**) predicted cell clusters by specified tools in the Human_retina_rpe_choroid dataset. Source data are provided as a Source Data file.

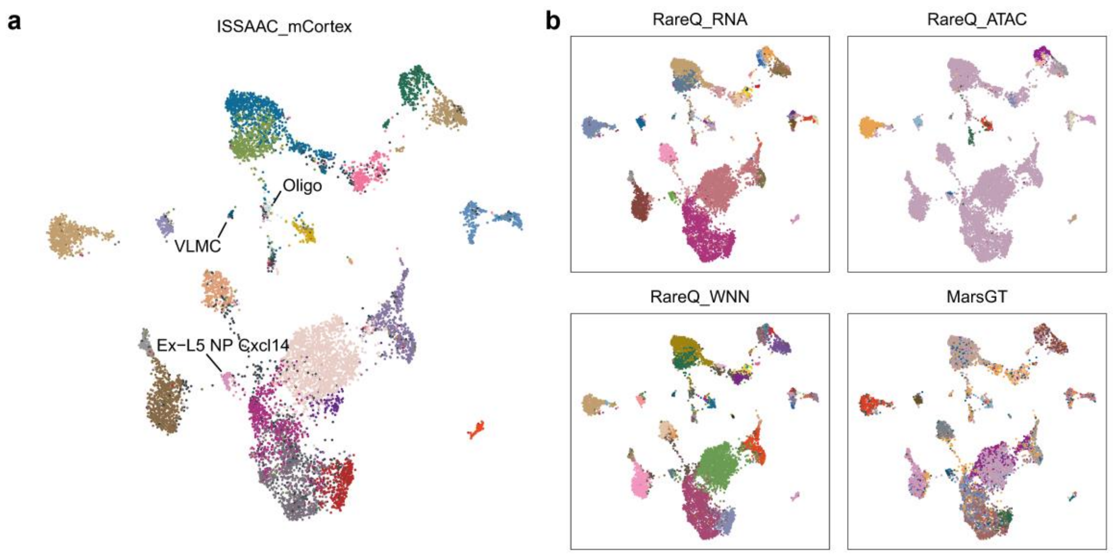

Supplementary Fig. 43 UMAPs on WNN integration visualizing (**a**) rare cell types (< 1% total cells, highlighted by labels) and (**b**) predicted cell clusters by specified tools in the ISSAAC_mCortex dataset. Source data are provided as a Source Data file.

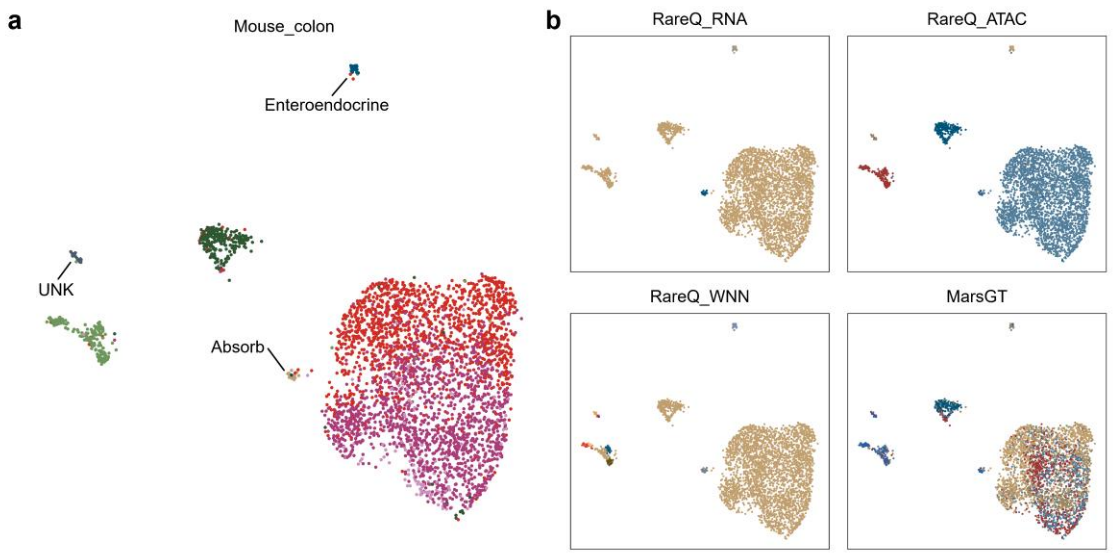

Supplementary Fig. 44 UMAPs on WNN integration visualizing (**a**) rare cell types (< 1% total cells, highlighted by labels) and (**b**) predicted cell clusters by specified tools in the Mouse_colon dataset. Source data are provided as a Source Data file.

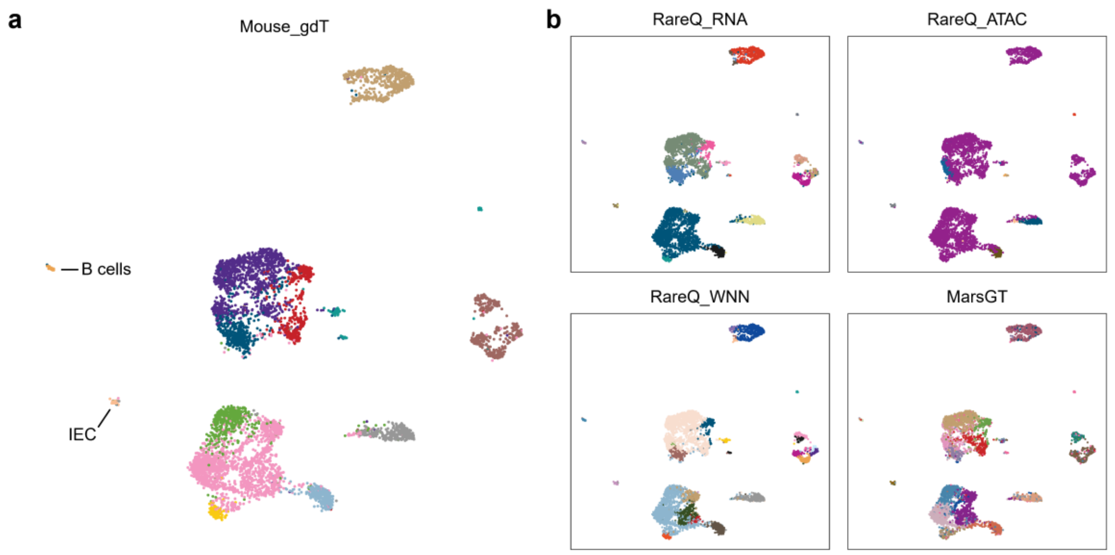

Supplementary Fig. 45 UMAPs on WNN integration visualizing (**a**) rare cell types (< 1% total cells, highlighted by labels) and (**b**) predicted cell clusters by specified tools in the Mouse_gdT dataset. Source data are provided as a Source Data file.

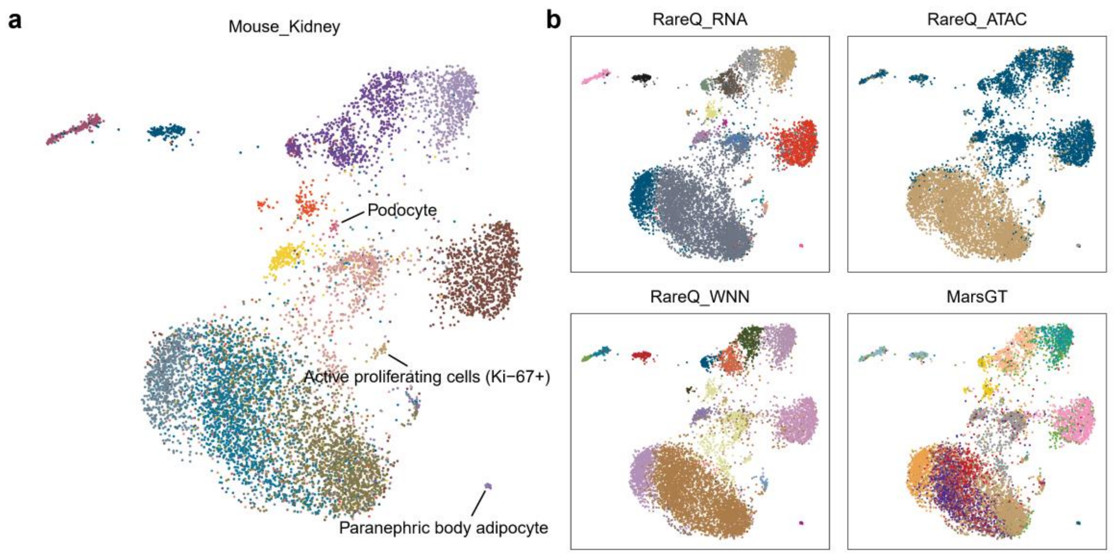

Supplementary Fig. 46 UMAPs on WNN integration visualizing (**a**) rare cell types (< 1% total cells, highlighted by labels) and (**b**) predicted cell clusters by specified tools in the Mouse_Kidney dataset. Source data are provided as a Source Data file.

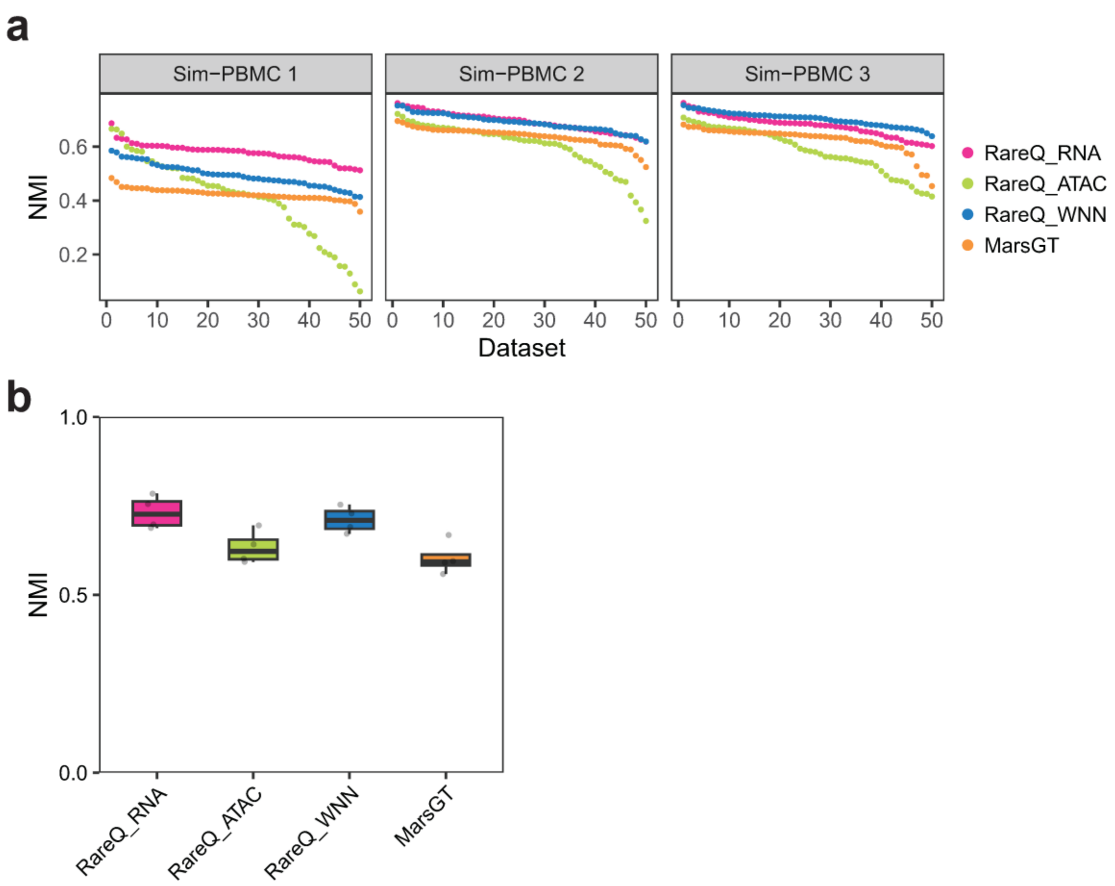

Supplementary Fig. 47 Benchmarking RareQ against MarsGT on global clustering performance via NMI metrics in both (**a**) paired PBMC simulated scRNA-seq and scATAC-seq datasets, (**b**) paired PBMC-bench-1, 2, 3 and 4 scRNA-seq and scATAC-seq datasets. Source data are provided as a Source Data file.

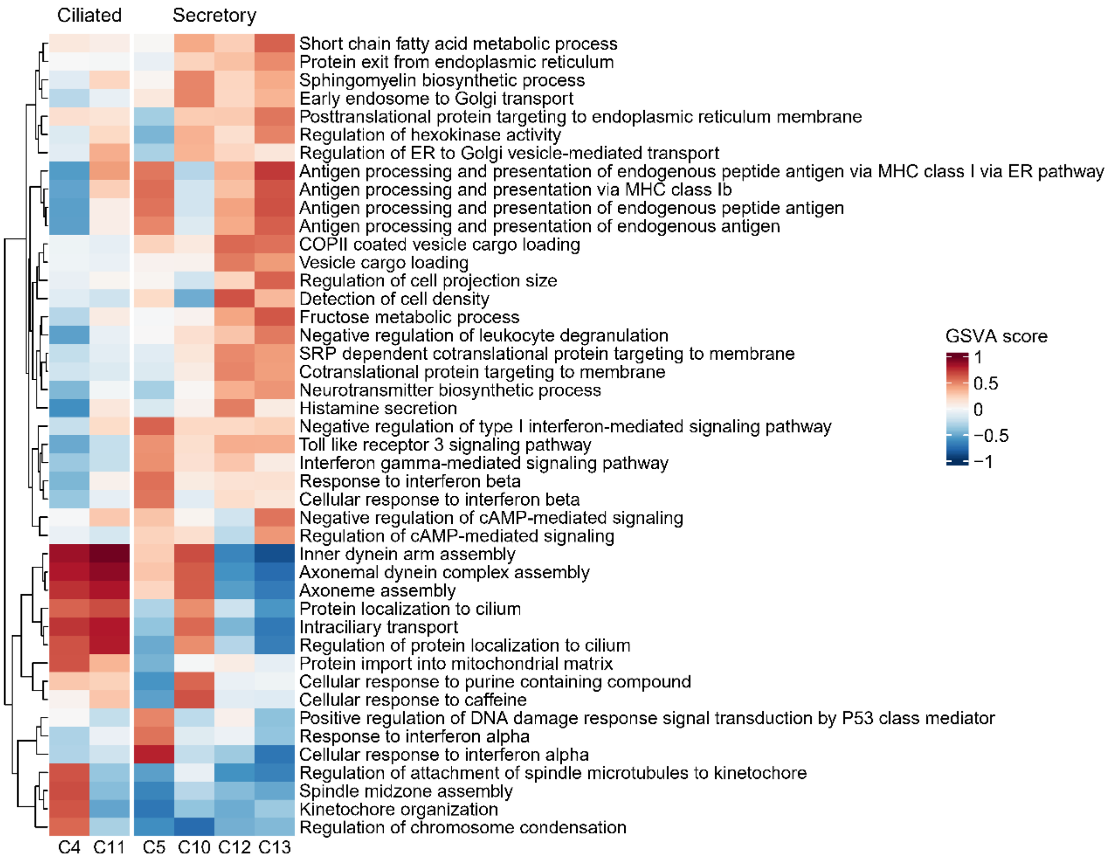

Supplementary Fig. 48 Heatmaps displaying the top-enriched pathways of clusters within ciliated and secretory compartments, respectively. Source data are provided as a Source Data file.

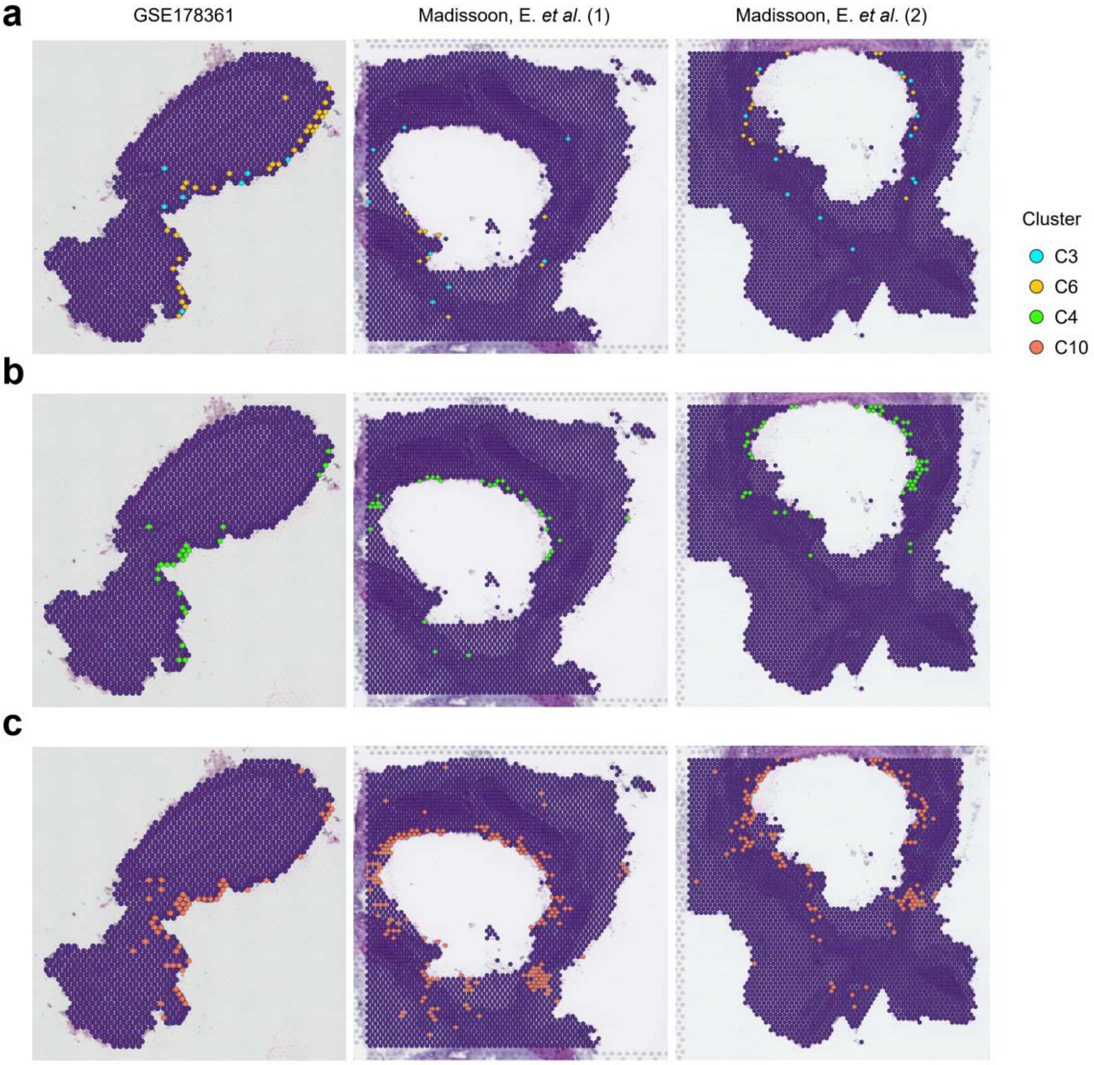

Supplementary Fig. 49. Validation of RareQ's unique rare airway epithelial populations using independent spatial transcriptomics. Spatial distribution of spots highly expressing marker genes of C3/C6 (**a**), C4 (**b**), and C10 (**c**). Markers of C3: TOP2A– MKI67+ H2AFZ+ KRT5+ TP63+ NGFR+; C6: TOP2A+ MKI67+ H2AFZ+ KRT5+ TP63+ NGFR+; C4: CCNO+ FOXJ1+ LRRC23+ MEIG1+ TPPP3+; C10: SCGB1A1+ FOXJ1+ LRRC23+ TUBA1A+ ISG15– IFIT3–. Source data are provided as a Source Data file.

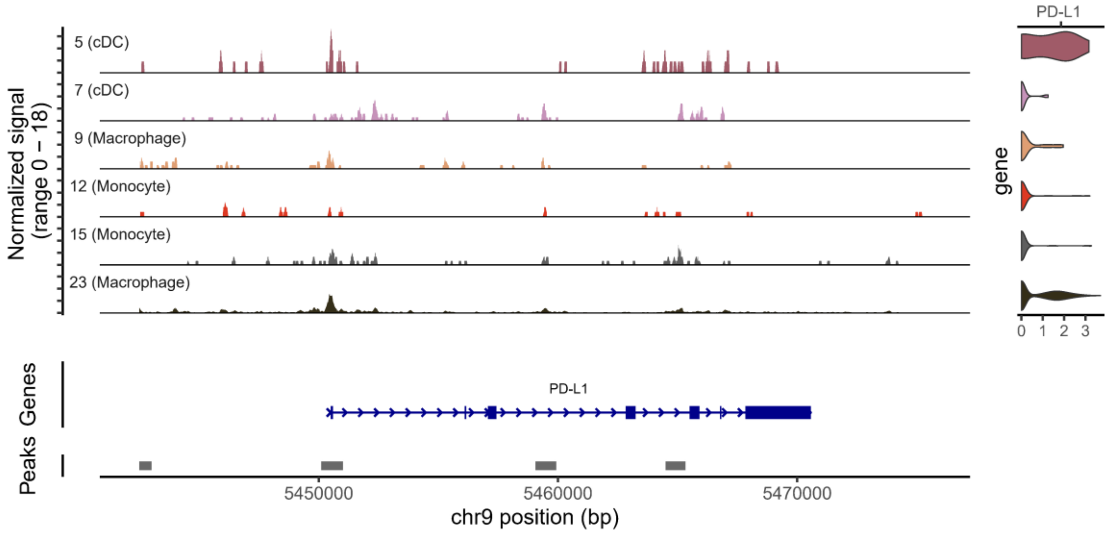

Supplementary Fig. 50 Coverage plots for gene *PD-L1* on B lymphoma data. Source data are provided as a Source Data file.

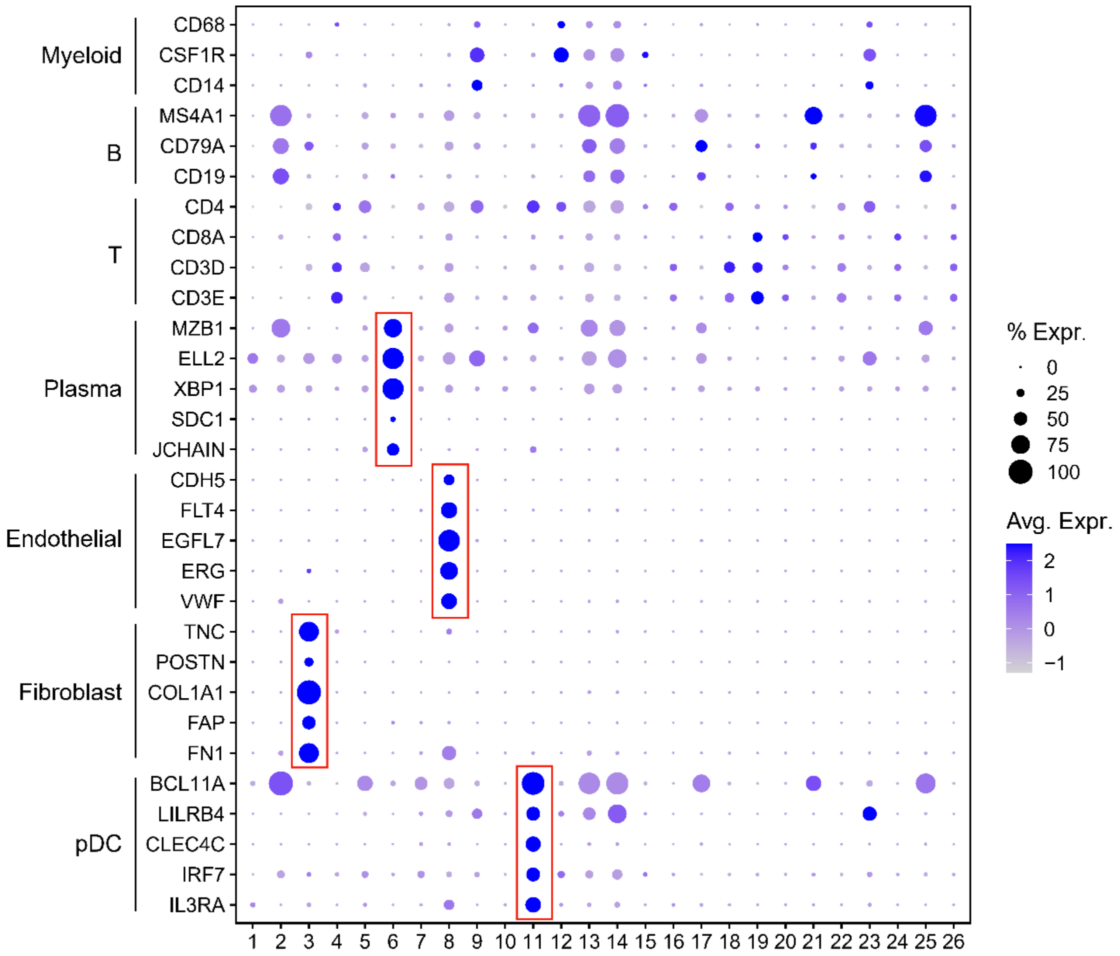

Supplementary Fig. 51 Dot plot illustrates the expression values and proportions of marker genes in clusters predicted by RareQ on B lymphoma data. Source data are provided as a Source Data file.

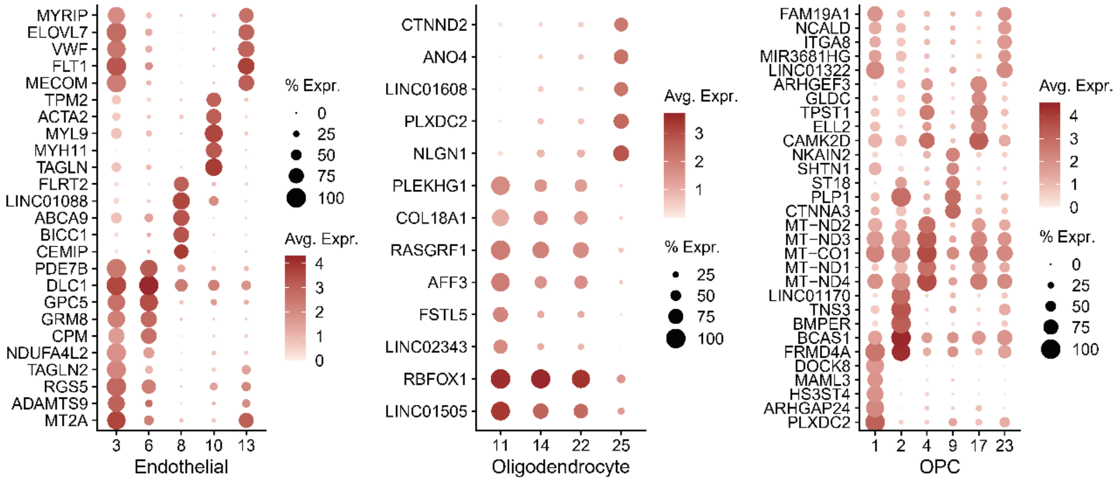

Supplementary Fig. 52 Dot plot illustrates the expression values and proportions of top DEGs within clusters of endothelial, oligodendrocyte and OPC predicted by RareQ in Alzheimer's Disease (AD) data. Source data are provided as a Source Data file.

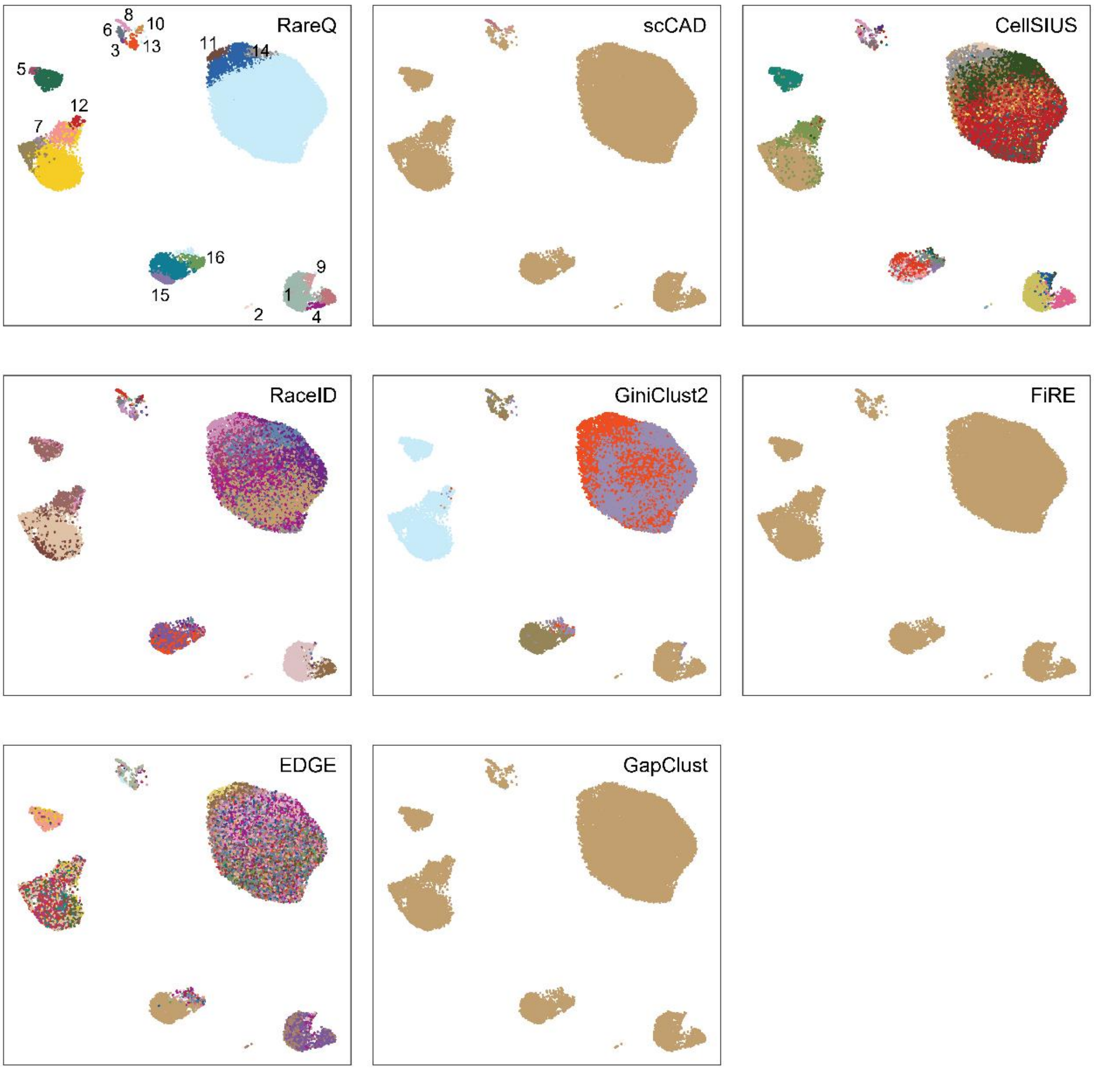

Supplementary Fig. 53 UMAP projections of the AD dataset colored by rare cell clusters predicted by the specified methods. Rare cell clusters (< 1% population) predicted by RareQ are highlighted for comparison. Source data are provided as a Source Data file.

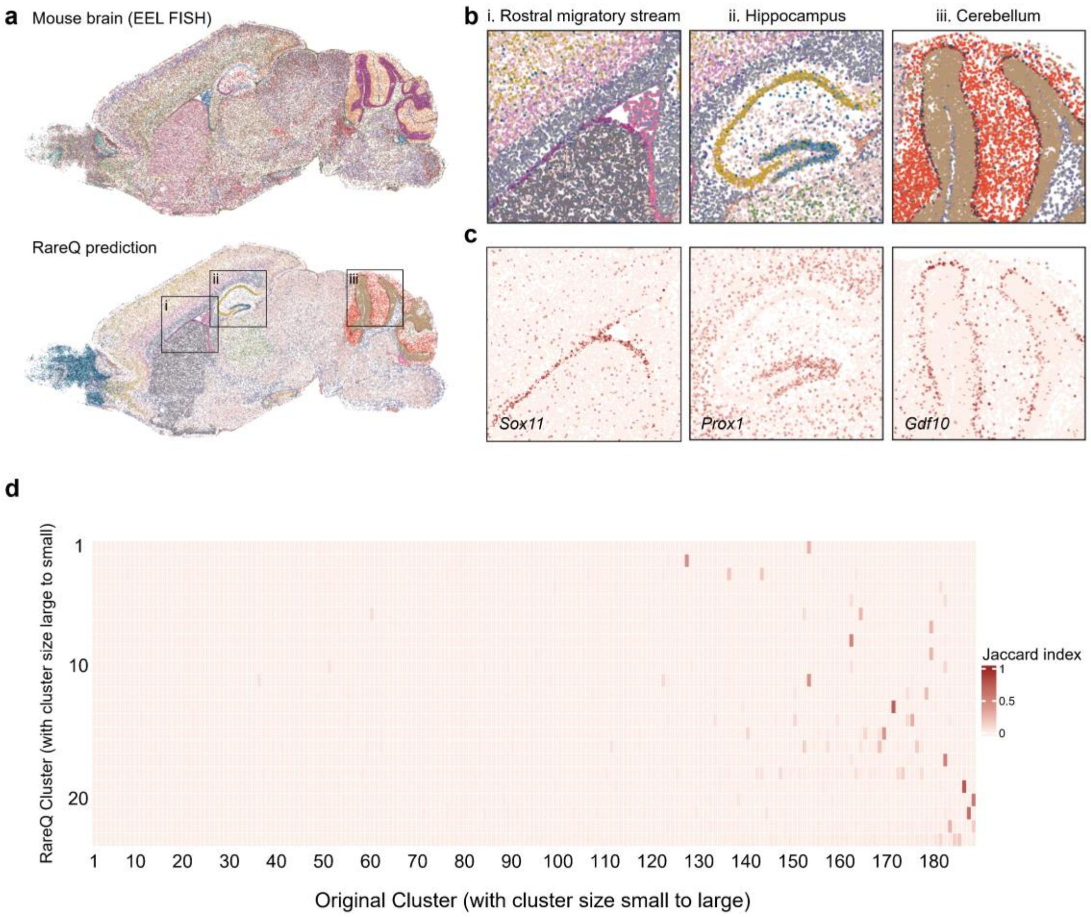

Supplementary Fig. 54 Spatial mapping of rare cells by RareQ in the EEL FISH mouse brain data. **a**, Spatial map of EEL FISH mouse brain data where every dot is a single cell colored by cluster identity reported by Borm et al. (top) and clusters predicted by RareQ (bottom). **b**, Magnified views of **a** showing the ventricle including the rostral migratory stream, the hippocampus and the cerebellum. **c**, Spatial expression of marker genes *Sox11*, *Prox1* and *Gdf10* in **b**. **d**, The side-by-side reconciliation comparison of between RareQ-derived annotations with and the original cluster labels. Source data are provided as a Source Data file.

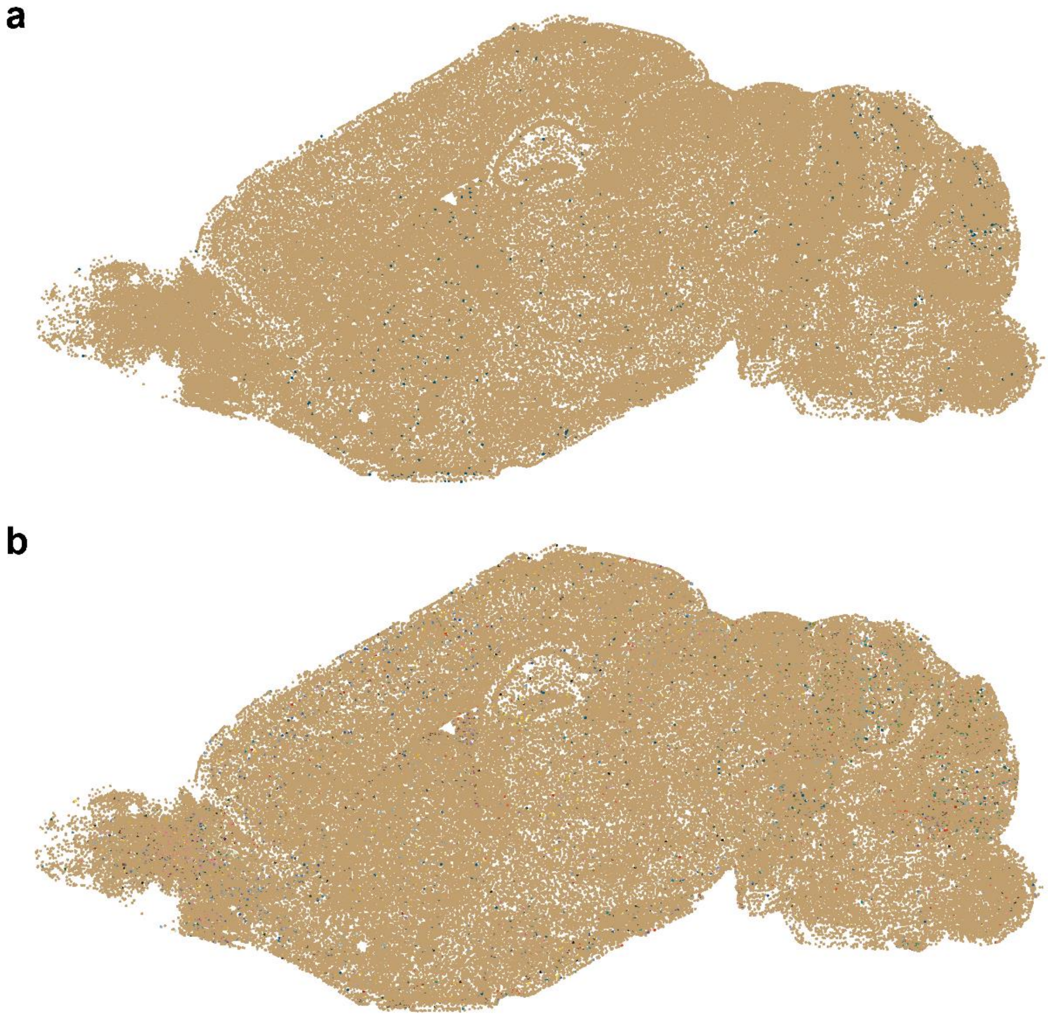

Supplementary Fig. 55 Rare cell types predicted by FiRE (**a**) and GapClust (**b**) in EEL FISH mouse brain data. Source data are provided as a Source Data file.

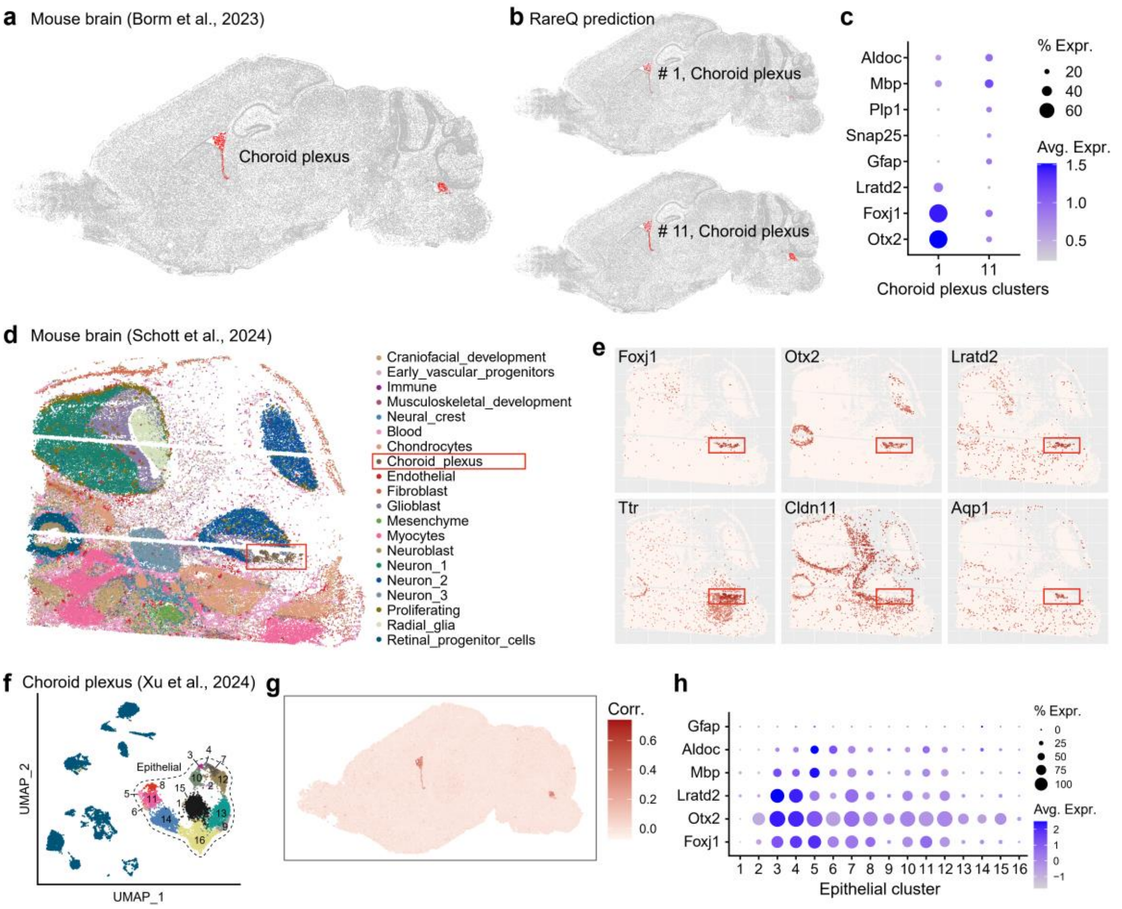

Supplementary Fig. 56. **Identification and characterization of choroid plexus cell subtypes.** **a**. Original annotated spatial map of the choroid plexus population. **b**. Spatial distribution of the C1 and C11 choroid plexus populations identified by RareQ. **c**. Bubble plot showing marker genes of the C1 population. **d**. Spatial distribution of diverse cell types of a mouse head sagittal section. **e**. Expression patterns and spatial localization of the choroid plexus marker genes. **f**. RareQ-identified choroid plexus cell populations in mouse brain snRNA-seq data. **g**. Cross-modal correlation of epithelial cells (snRNA-seq) with all cells in the EEL FISH spatial dataset. **h**. Bubble plot illustrating marker genes for the c1 choroid plexus population within the snRNA-seq cell clusters. Source data are provided as a Source Data file.

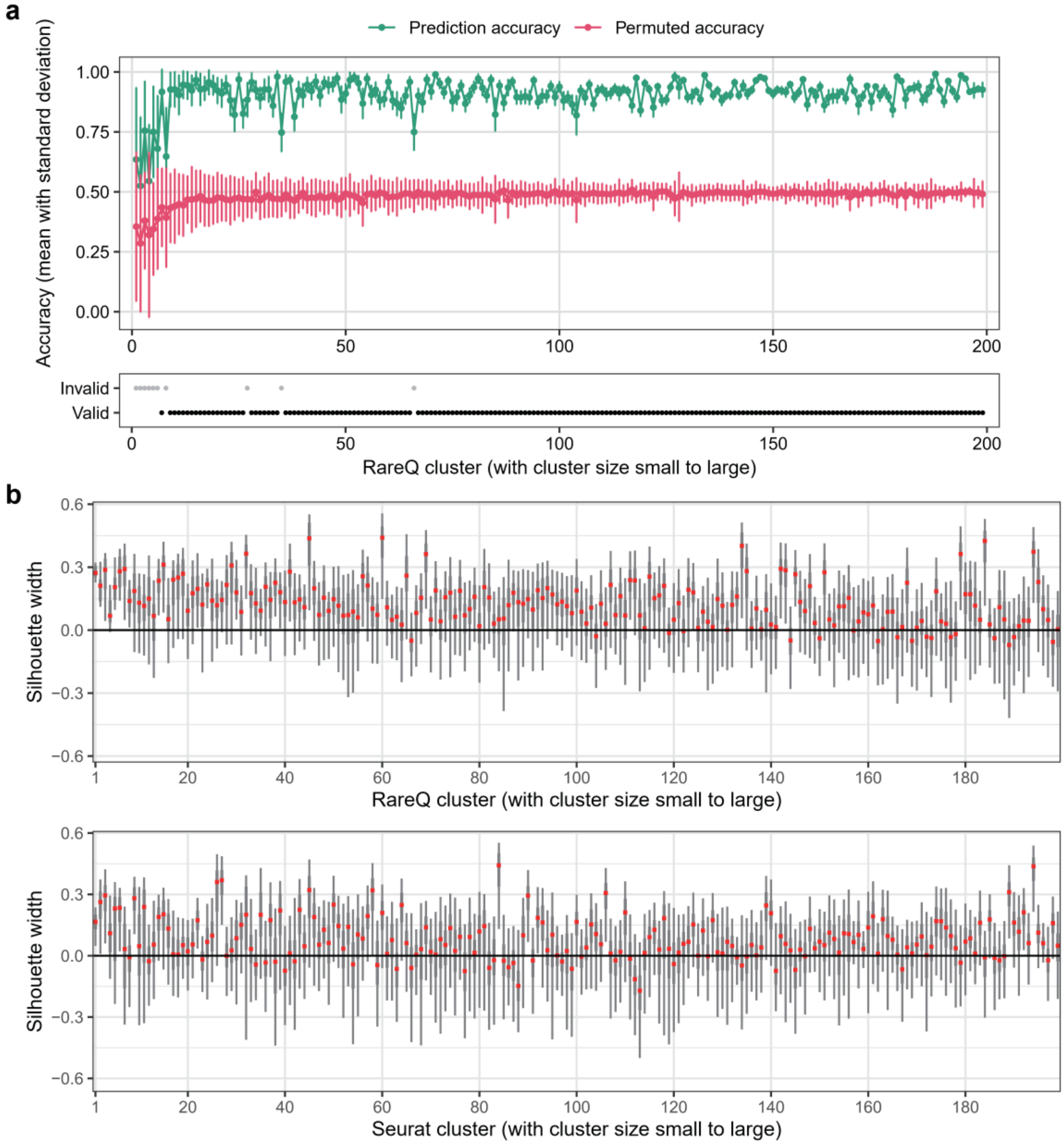

Supplementary Fig. 57 a, CHOIR-based testing between each cluster and its nearest neighbor predicted by RareQ in Xenium mouse brain data. Valid means the cluster is significantly different from its neighboring cluster and should be split from each other, while invalid means two clusters are not significantly different and should be merged. **b**, Assessment of cluster quality using silhouette coefficient for RareQ-derived clusters and Seurat-derived clusters on Xenium spatial transcriptomic data. Source data are provided as a Source Data file.

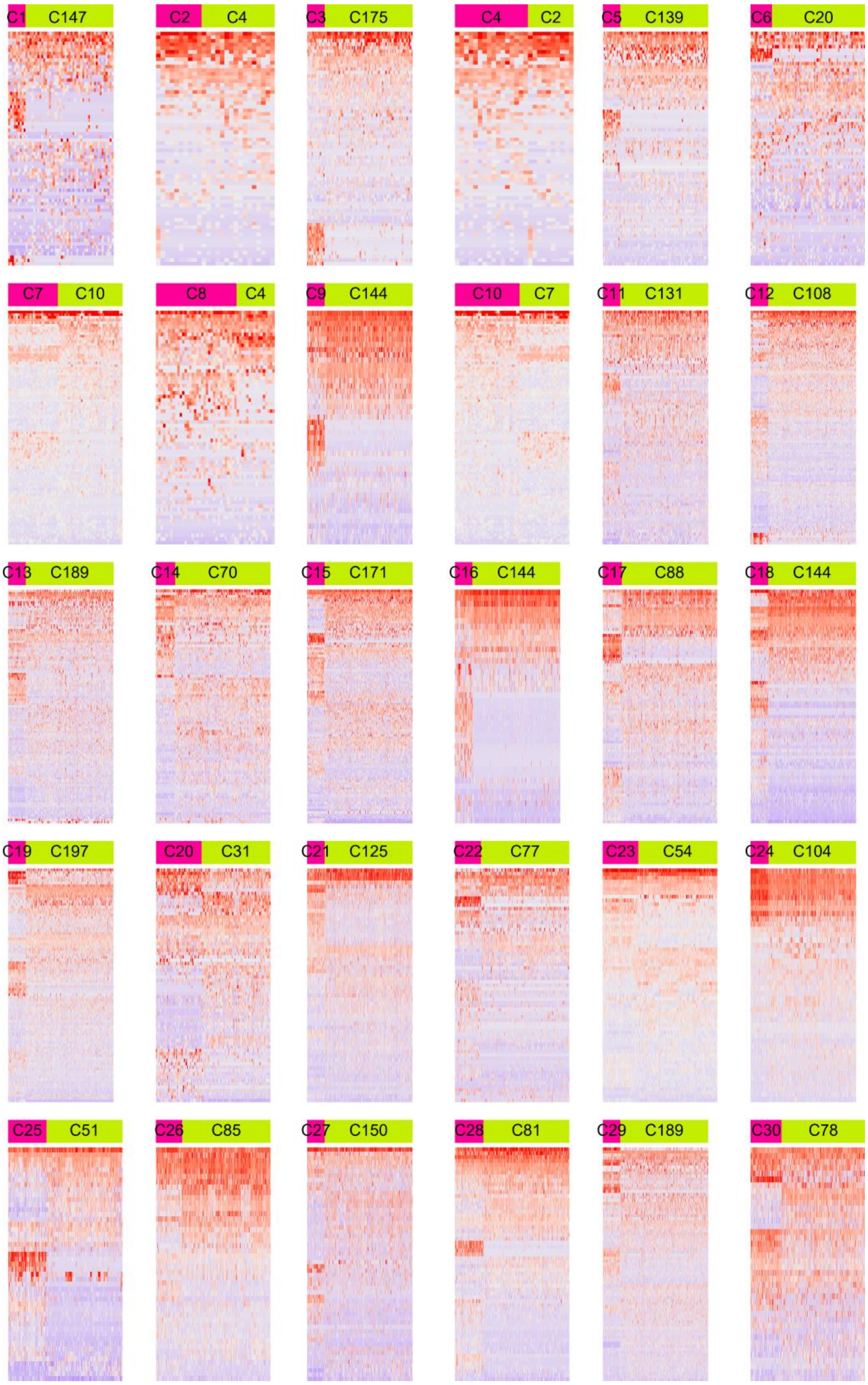

Supplementary Fig. 58 Heatmap illustrating the molecular differences between RareQ-specific rare cell clusters and their similar major cell types on Xenium spatial transcriptomic data. Source data are provided as a Source Data file.

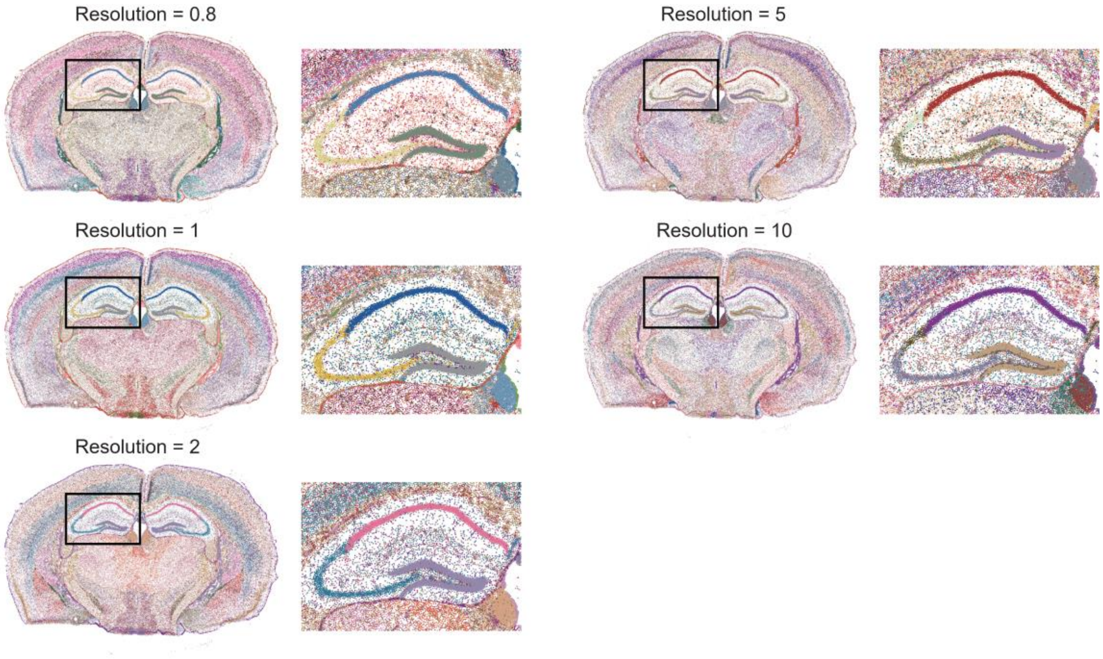

Supplementary Fig. 59 Spatial maps of Xenium mouse brain data, where every dot is a single cell colored by clusters predicted by Seurat at specified resolutions and a magnified view showing the hippocampus labeled by identified clusters. Source data are provided as a Source Data file.

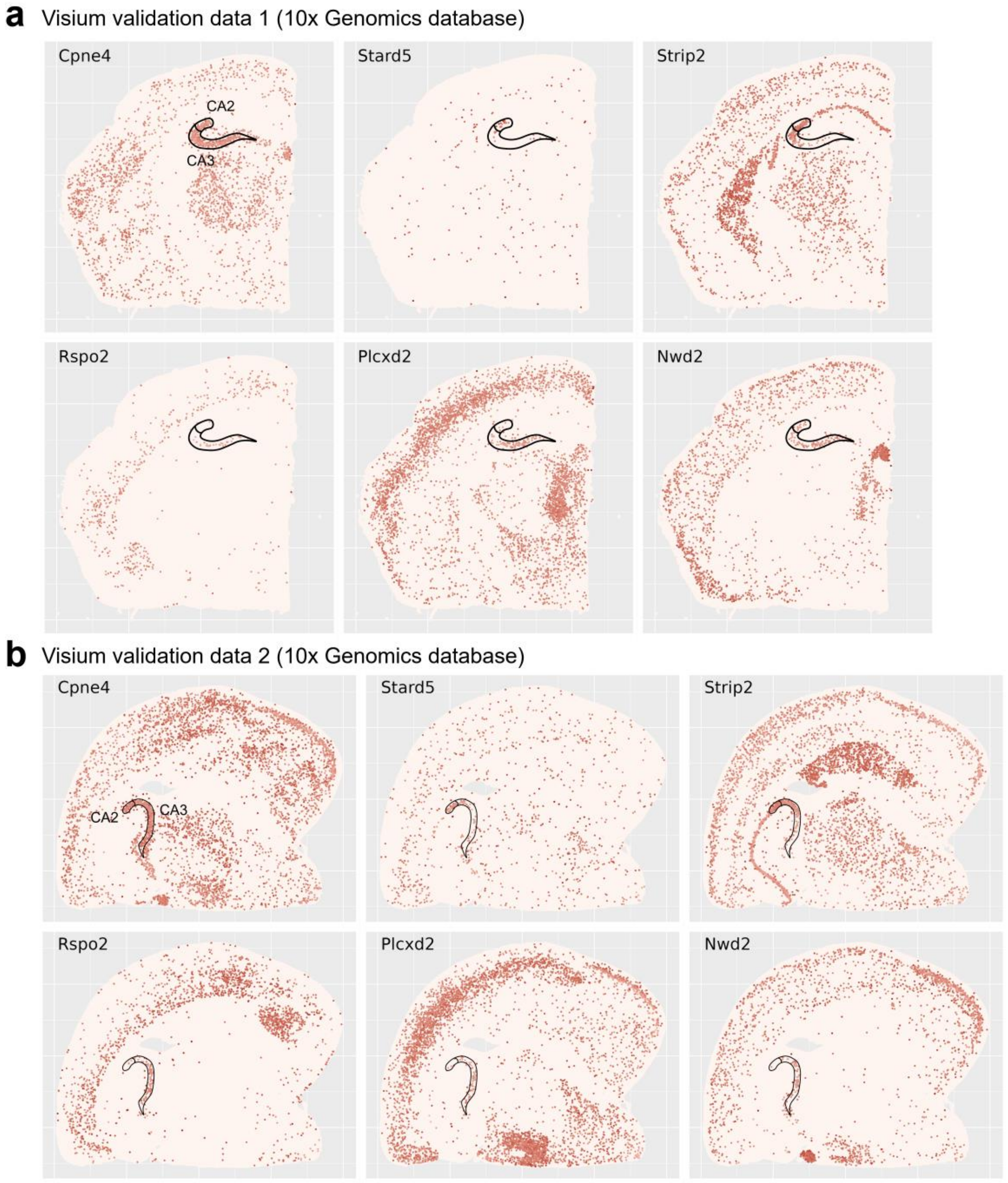

Supplementary Fig. 60 Independent spatial transcriptomics datasets validate the spatial localization of CA3 subpopulations and their marker gene's expression. a. Expression patterns of marker genes for CA2, CA3, and the two CA3 subtypes in the mouse brain spatial transcriptomics dataset. **b**. Same as (**a**), showing the expression patterns in another mouse brain dataset. Markers of CA3_1: Strip2; CA3_2: Rspo2, Plcxd2, Nwd2. Source data are provided as a Source Data file.

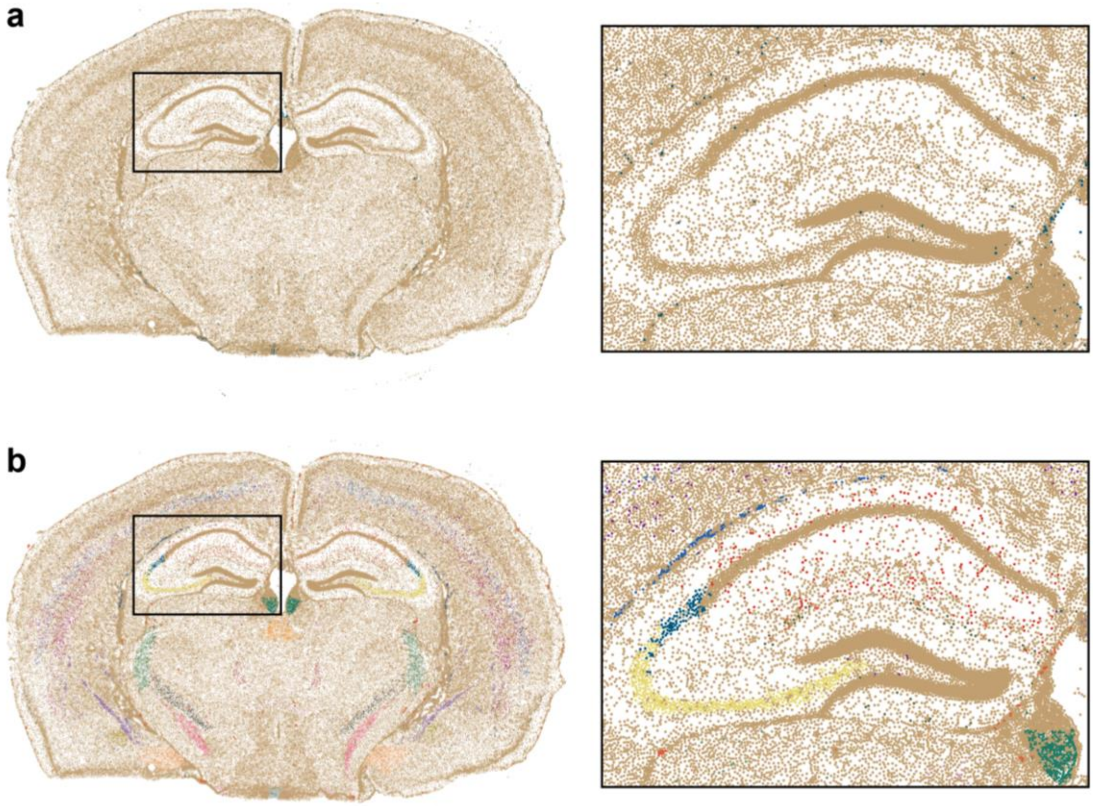

Supplementary Fig. 61 Rare cell types predicted by FiRE (**a**) and scCAD (**b**) in Xenium mouse brain data. Source data are provided as a Source Data file.

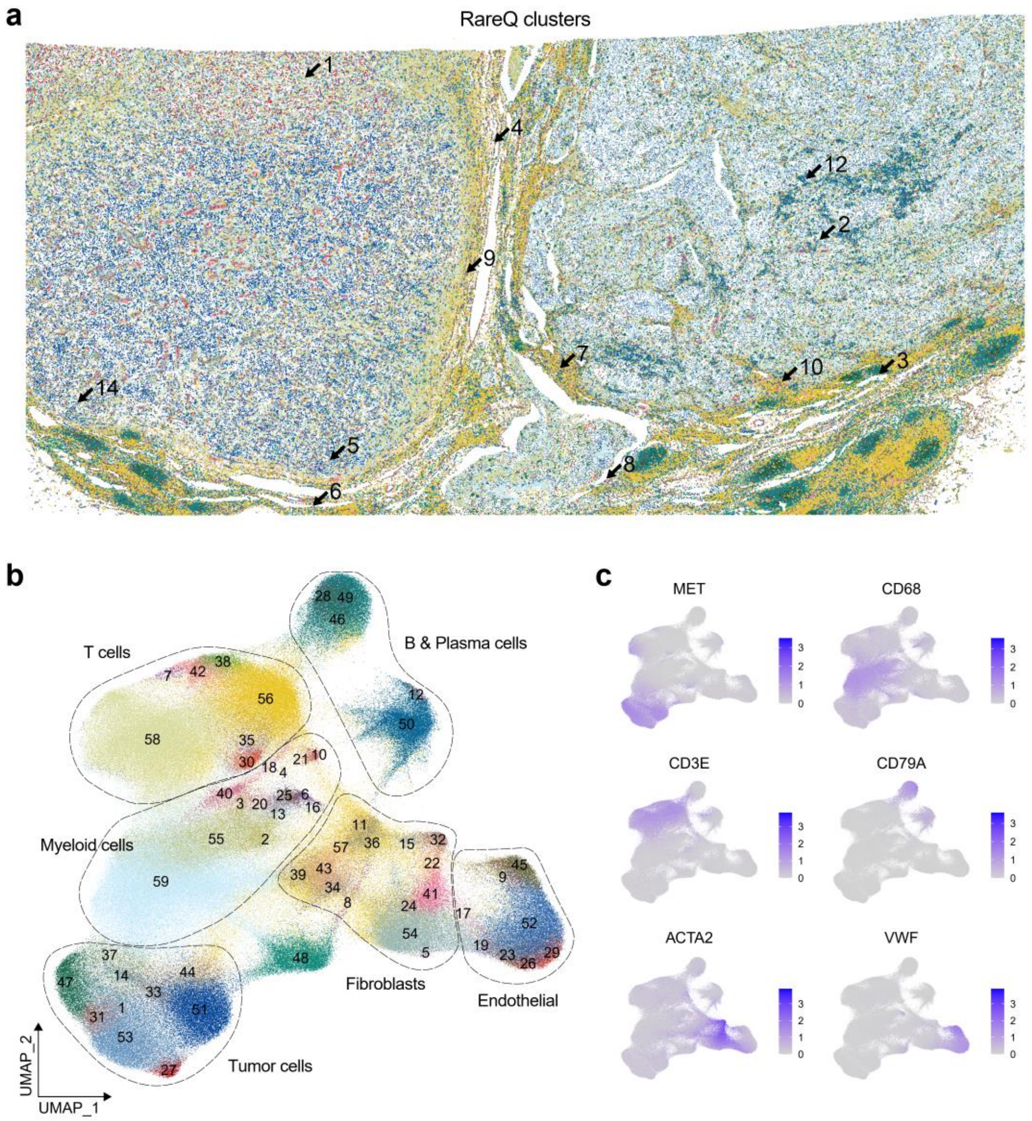

Supplementary Fig. 62 Clusters predicted by RareQ in a multiome (RNA and ADT) spatial data from human renal cell carcinoma (RCC) by the Xenium platform. **a**, Spatial maps of RCC data, where every dot is a single cell colored by clusters predicted by RareQ, with selected rare cell clusters highlighted. **b**, WNN integration-based UMAP plot of RCC data colored by RareQ clusters. **c**, UMAP projects illustrating the expression levels of cell markers for annotation.

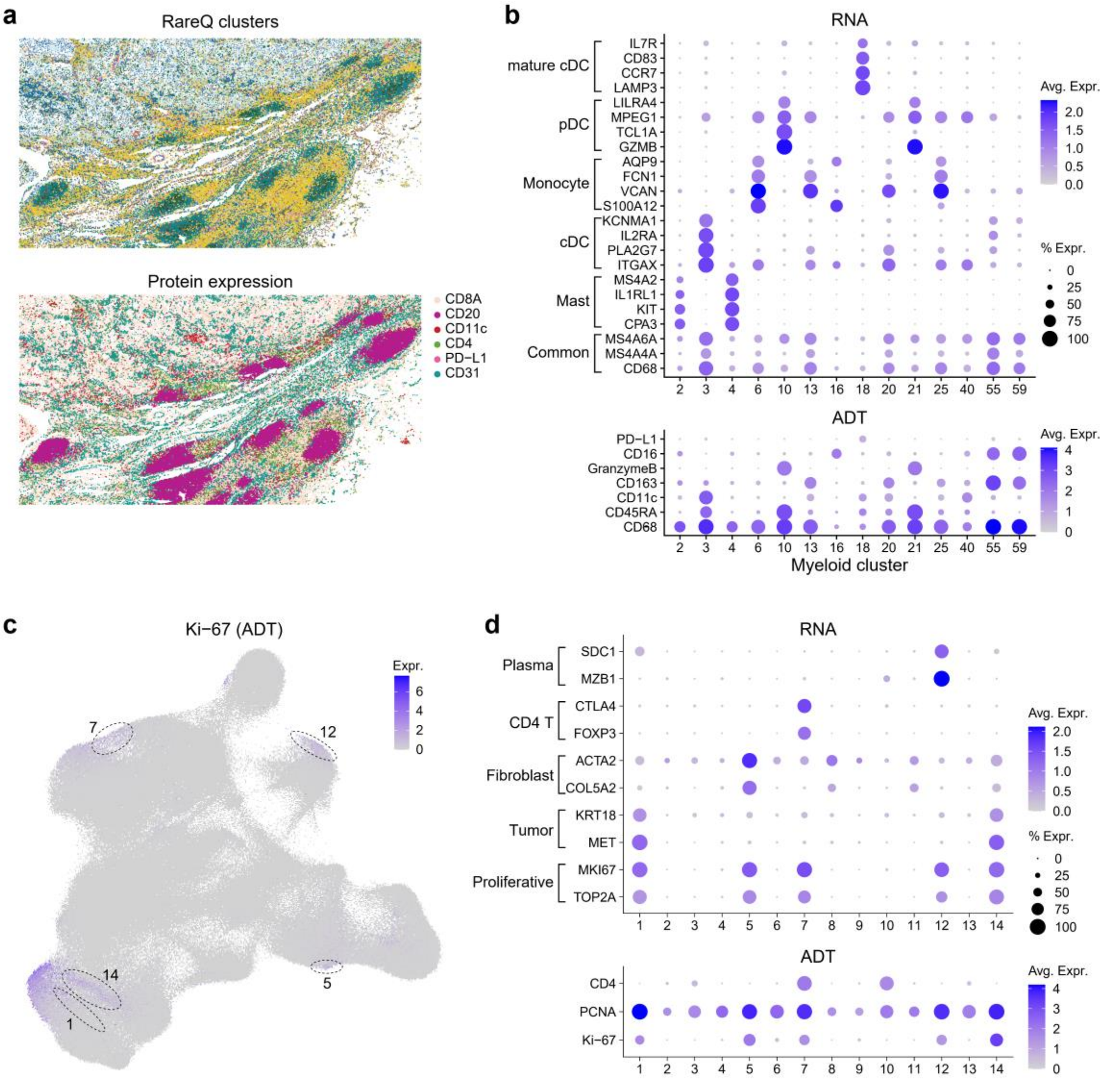

Supplementary Fig. 63 RareQ identifies rare myeloid and proliferative cell types in multiome spatial RCC data. **a**, Zoomed view of spatial maps of RCC data showing tertiary lymphoid structures (TLS) colored by RareQ clusters and surface protein markers. **b**, Dot plot illustrating the expression values and proportions of marker genes and proteins in rare clusters in the myeloid compartment. **c**, UMAP plot of rare cell clusters with high expression of Ki-67 protein highlighted. **d**, Dot plot illustrating the expression values and proportions of marker genes and proteins in highlighted rare clusters in (**c**).

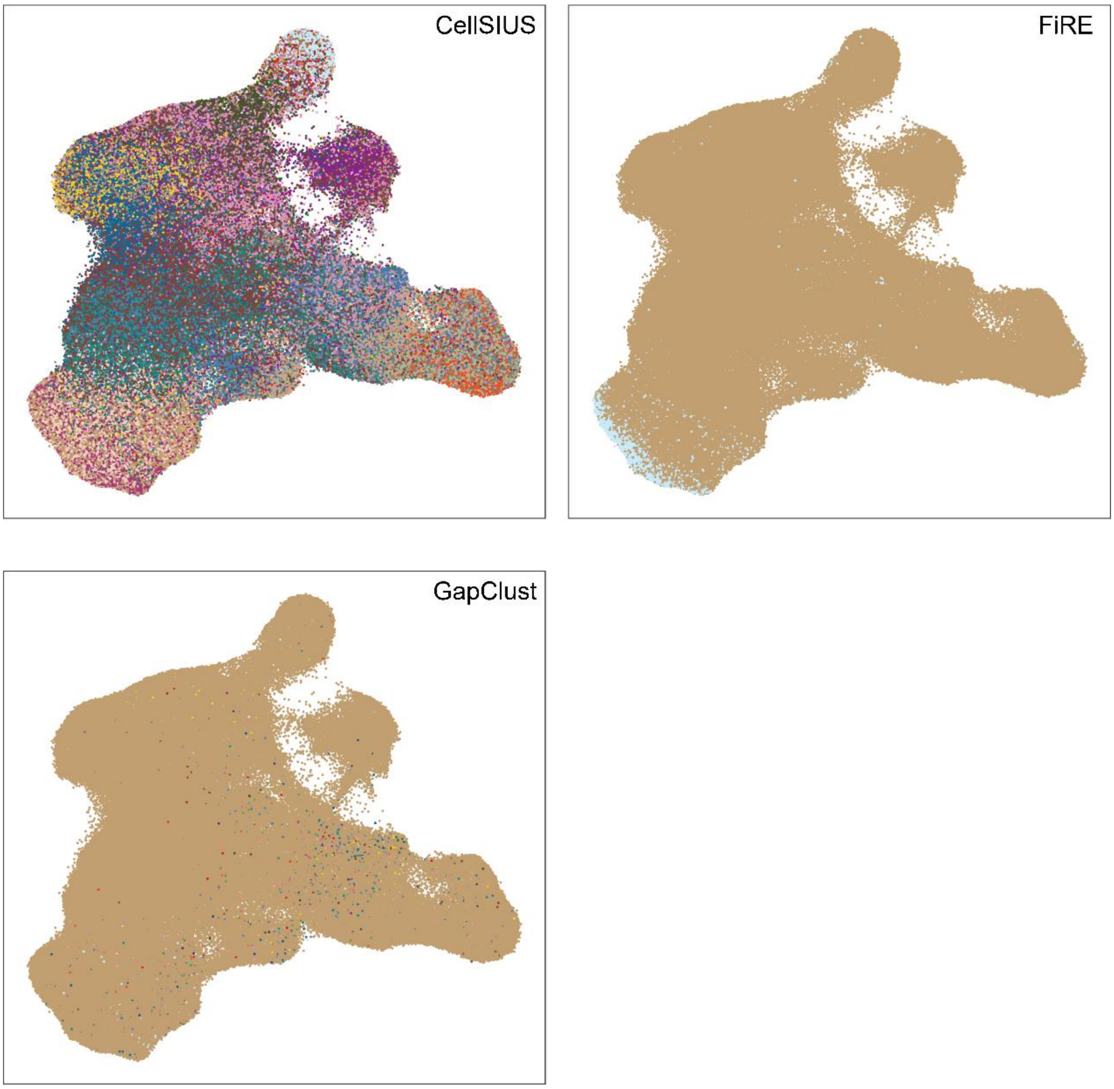

Supplementary Fig. 64 UMAP projects of multiome spatial RCC data colored by rare cell types predicted by CellSIUS, FiRE and GapClust.

Supplementary Tables

Supplementary Table 1 Detailed information on the number of cell types in PBMC simulation datasets.

**Scenarios**	**Number of cells**	**Number of major cell types**	**Number of rare cell types**
Sim-PBMC-1	5,000	4	1
Sim-PBMC-2	5,000	9	1
Sim-PBMC-3	5,000	10	5

Supplementary Table 2 Detailed information on the 20 scRNA-seq datasets used in this study.

Dataset	Cells	Genes	Types	Accession	Description
Airway	7193	18388	7	GSE103354	mouse tracheal epithelium cells
Arc-ME	20921	26774	11	GSE93374	mouse hypothalamic arcuate–median eminence complex
B_lymphoma	14085	19107	14	10X genomics	lymph node tumor
Cao	7603	20271	41	sci-RNA-seq platform	worm neuron cells
Chen	12089	23284	46	GSE87544	mouse hypothalamus
Choroid_plexus	16291	32285	26	GSM7663129	mouse choroid plexus
Cortex	4007	27998	13	GSM3580745	mouse cerebral cortex in hypoxia
Heart	4365	23341	8	https://cblast.gao-lab.org/download	mouse heart
Kidney_ccRCC	20748	33694	14	GSE159115	Human kidney clear cell RCC
Kidney_normal	6146	33694	28	GSE159115	Human kidney benign adjacent
MacParland	8444	20007	20	GSE115469	human liver cells
Macosko	44808	23288	12	GSE63473	mouse retina cells
Mammary	25010	27998	20	GSE106273	mammary epithelial cells
Pancreas	8569	20125	14	GSE84133	human pancreas
Pediatric_gut	22502	33694	41	https://www.gutcellatlas.org/	pediatric terminal ileal
Plasschaert	6977	28205	8	https://cblast.gao-lab.org/download	mouse airway epithelial cells
Retina	9383	6198	16	GSE201402	mouse retina
Shekhar	27499	13166	19	GSE81905	mouse retina cells
UUOkidney	6147	21516	17	GSE119531	mouse kidney from unilateral ureteral obstruction (UUO)
Zelsel	3005	19972	7	GSE60361	mouse cortex and hippocampus

Supplementary Table 3 Detailed information on the running time (hours) of benchmarking tools across the simulation datasets ranging from 1,000 to 200,000 cells. NA means running into a memory error.

	**1,000**	**2,000**	**5,000**	**10,000**	**20,000**	**50,000**	**100,000**	**200,000**
**RareQ**	**0.0011**	**0.0014**	**0.0030**	**0.0074**	**0.0156**	**0.0412**	**0.0932**	**0.2097**
**RaceID**	0.0090	0.0211	0.1378	0.3914	1.5777	24.5976	NA	NA
**GapClust**	0.0024	0.0082	0.0251	0.0502	0.1188	0.2922	0.5902	1.2506
**FiRE**	0.0007	0.0013	0.0021	0.0036	0.0079	0.0211	0.0528	0.1206
**CellSIUS**	0.0057	0.0157	0.0467	0.1318	0.3620	1.7688	5.9248	31.9549
**EDGE**	0.0039	0.0090	0.0356	0.1111	0.3679	3.8688	14.5735	NA
**GiniClust2**	0.0122	0.0321	0.1819	0.5626	2.2150	14.6479	63.5333	NA
**scCAD**	0.0058	0.0411	0.1188	0.2315	0.5214	0.5803	3.3721	9.7836

Supplementary Table 4 Detailed information on the memory usage (GB) of benchmarking tools across the simulation datasets ranging from 1,000 to 200,000 cells. NA means running into a memory error.

	**1,000**	**2,000**	**5,000**	**10,000**	**20,000**	**50,000**	**100,000**	**200,000**
**RareQ**	**0.3140**	**0.6165**	**0.8281**	**1.8436**	**3.4780**	**7.8256**	**17.4786**	**34.5050**
**RaceID**	0.6429	0.8710	2.4775	6.5737	20.9189	139.6542	NA	NA
**GapClust**	0.3753	0.8883	2.1130	2.5261	4.9465	12.0626	24.6929	48.8916
**FiRE**	0.9003	1.7878	4.5008	8.9902	7.9450	17.9975	36.0724	72.0543
**CellSIUS**	0.8591	1.6230	3.9541	7.3926	15.4025	38.1441	74.2886	148.5935
**EDGE**	0.3515	0.7321	1.7298	3.3811	6.7106	16.6038	35.9568	NA
**GiniClust2**	0.8812	0.8530	3.5405	11.9223	43.9620	113.6324	253.8545	NA
**scCAD**	0.2306	0.4597	1.1319	2.2983	4.5857	11.3098	22.9045	45.7989

Supplementary Table 5 Detailed information on the 10 multiome (RNA+ATAC) single-cell datasets used in this study.

Dataset	Cells	Types	Accession	Description
10x_Multiome_PBMC_Chromium	9517	17	https://www.biosino.org/scMMO-atlas/	Human peripheral blood mononuclear cells
10x_Multiome_PBMC10k	9631	19	https://scglue.readthedocs.io/en/latest/data.html	Human peripheral blood mononuclear cells
Chen_2019	9190	22	https://scglue.readthedocs.io/en/latest/data.html	Mouse cerebral cortex
Human_Gray_matter	7864	20	GSE193240	Human brain gray matter
Human_retina	16325	16	https://www.biosino.org/scMMO-atlas/	Human retina
Human_retina_rpe_choroid	6423	10	https://www.biosino.org/scMMO-atlas/	Human retinal pigment epithelium (RPE) and choroid
ISSAAC_mCortex	10361	23	https://www.biosino.org/scMMO-atlas/	Mouse cerebral cortex
Mouse_Kidney	8837	14	GSE117089	Mouse kidney
Mouse_colon	3529	8	https://www.biosino.org/scMMO-atlas/	Mouse colon
Mouse_gdT	5887	13	https://www.biosino.org/scMMO-atlas/	Mouse gamma delta T cells

Supplementary Table 6 Differential abundance of cell subtypes in AD vs. healthy controls

Cluster	*Log2 FC*	*LogCPM*	*F-statistic*	*p value*
1	*-1.5403*	*10.1705*	*1.0636*	*0.304*
2	*3.6042*	*10.0398*	*4.0582*	*0.0457*
3	*0.8819*	*10.6279*	*0.3307*	*0.566*
4	*2.0888*	*11.4919*	*1.1927*	*0.2765*
5	*5.1107*	*11.502*	*4.3424*	*0.0388*
6	*-0.0828*	*11.9391*	*0.0028*	*0.9573*
7	*-2.9078*	*12.1873*	*3.2537*	*0.0732*
8	*-1.5442*	*12.4684*	*1.0134*	*0.3156*
9	*0.0329*	*11.6586*	*0.0003*	*0.9848*
10	*-0.0552*	*12.4467*	*0.0014*	*0.9701*
11	*-3.4253*	*12.5718*	*4.1951*	*0.0422*
12	*1.0213*	*12.0423*	*0.4811*	*0.4889*
13	*-0.2559*	*13.0149*	*0.0393*	*0.8429*
14	*0.778*	*12.6065*	*0.3025*	*0.583*
15	***3.5935***	***13.1931***	***5.4319***	***0.0211***
16	*0.0236*	*13.1318*	*0.0003*	*0.9852*
17	*5.1205*	*13.405*	*8.8075*	*0.0034*
18	*-2.2309*	*14.7938*	*2.1299*	*0.1465*
19	*-4.5116*	*14.8527*	*6.594*	*0.0112*
20	*4.0969*	*14.7445*	*5.3698*	*0.0218*
21	*0.2677*	*15.765*	*0.0533*	*0.8177*
22	*0.7794*	*14.9526*	*0.3262*	*0.5687*
23	*-0.5291*	*16.2058*	*0.2086*	*0.6484*
24	*-0.0865*	*17.4168*	*0.0055*	*0.9413*
25	*0.322*	*19.3085*	*0.3615*	*0.5485*

Supplementary Notes

Supplementary Note 1 Ablation studies to isolate RareQ's core components and evaluate their individual contributions to rare-cell detection and overall clustering accuracy.

To assess the importance of RareQ's design, we performed ablation studies in which the Q-guided propagation was replaced by uniform diffusion (equivalent to assigning a constant $Q = 0$). This substitution resulted in decreased F_1 scores, precision, and recall for several rare clusters (**Supplementary Fig. 65a–e**), underscoring the essential role of Q-guided propagation in accurately identifying rare cell populations. We speculated that this decline specifically affects clusters that have weak separation from dominant populations, as those completely isolated within the kNN graph would be easily detected by conventional methods. To test our hypothesis, we created detection difficulty through two distinct approaches: (1) we subsampled rare cells from major clusters and perturbed the expression of 10–100 genes by three-fold up regulation (**Supplementary Fig. 65f**). (2) we adapted the real data shown in Supplementary Figure 23 and incorporated varying numbers of differentially expressed genes into the real rare clusters (**Supplementary Fig. 65g**). The results showed that RareQ consistently outperformed the ablated version, confirming that Q-guided propagation enhances identification of challenging populations.

Following propagation, RareQ employs a recursive merging strategy driven by the cluster-level Q metric (Q_c). Merges are accepted only if the resulting cluster exhibits a higher Q_c value, reflecting increased internal connectivity. This provides a biologically interpretable, topology-based criterion that prevents over-clustering while preserving stable rare-cell clusters. Ablation analysis omitting this step led to a slightly increased F_1 score but significantly decreased NMI, confirming that ΔQ_c-guided merging significantly improves overall global clustering accuracy, despite potential underclustering in a few clusters (**Supplementary Fig. 66**).

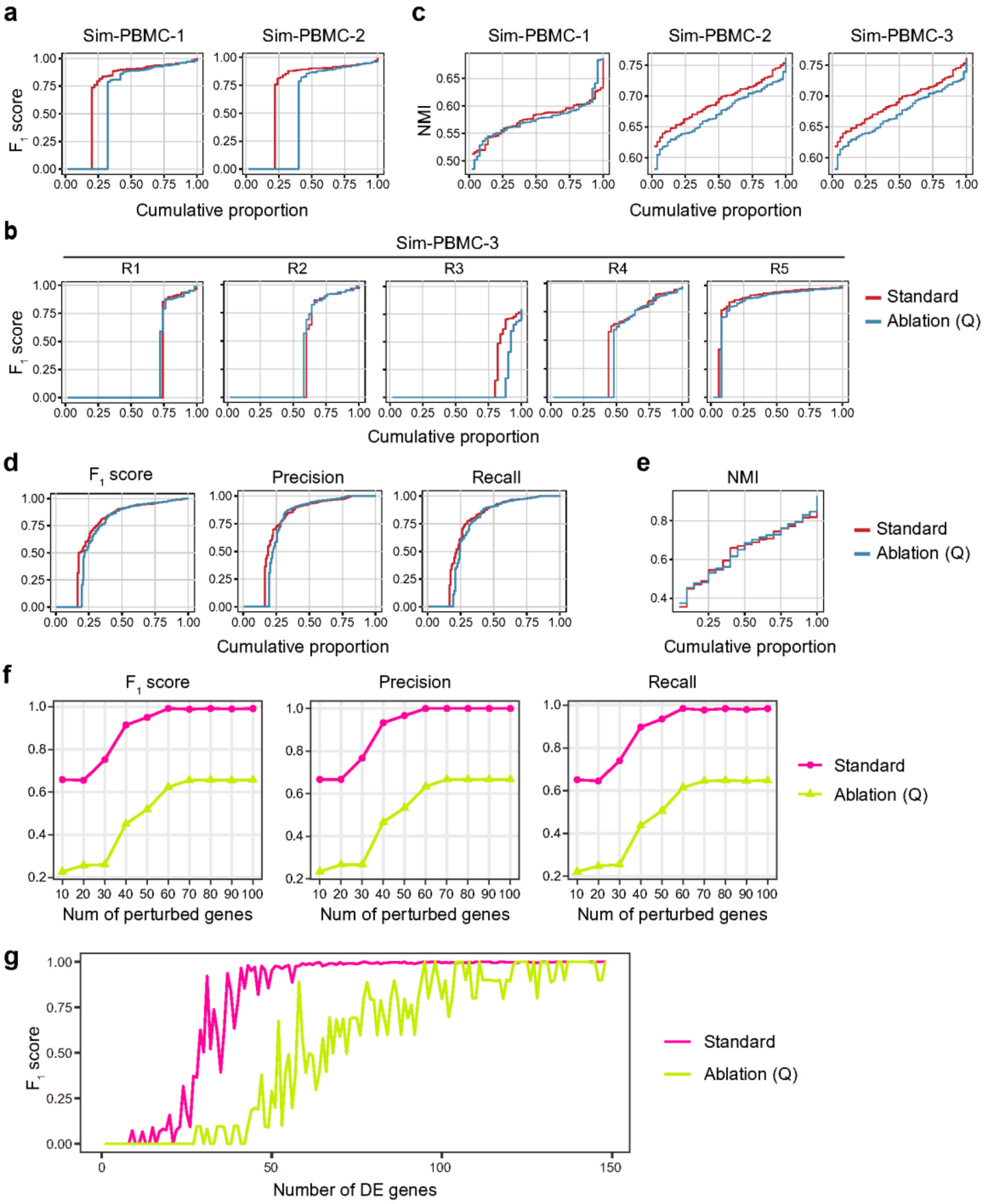

Supplementary Fig. 65 Ablation analyses of *Q*-value demonstrate its contribution to rare-cell identification across simulation and real datasets. a. Cumulative curves comparing F_1 scores before and after *Q*-value ablation in simulated datasets 1 and 2. **b.** Same as (**a**), but based on simulated dataset 3. **c.** Same as (**a**), but for NMI comparison in three simulated datasets. **d.** Comparison of F_1 score, precision, and recall before and after *Q*-value ablation in real datasets. **e.** Same as (**d**), but for NMI comparison in the real datasets. **f.** Ablation analysis of the impact of *Q*-value under simulated conditions with varying degrees of gene-expression perturbations. **g.** Same as (**f**), but examining the impact of varying the number of differentially expressed genes in the simulation.

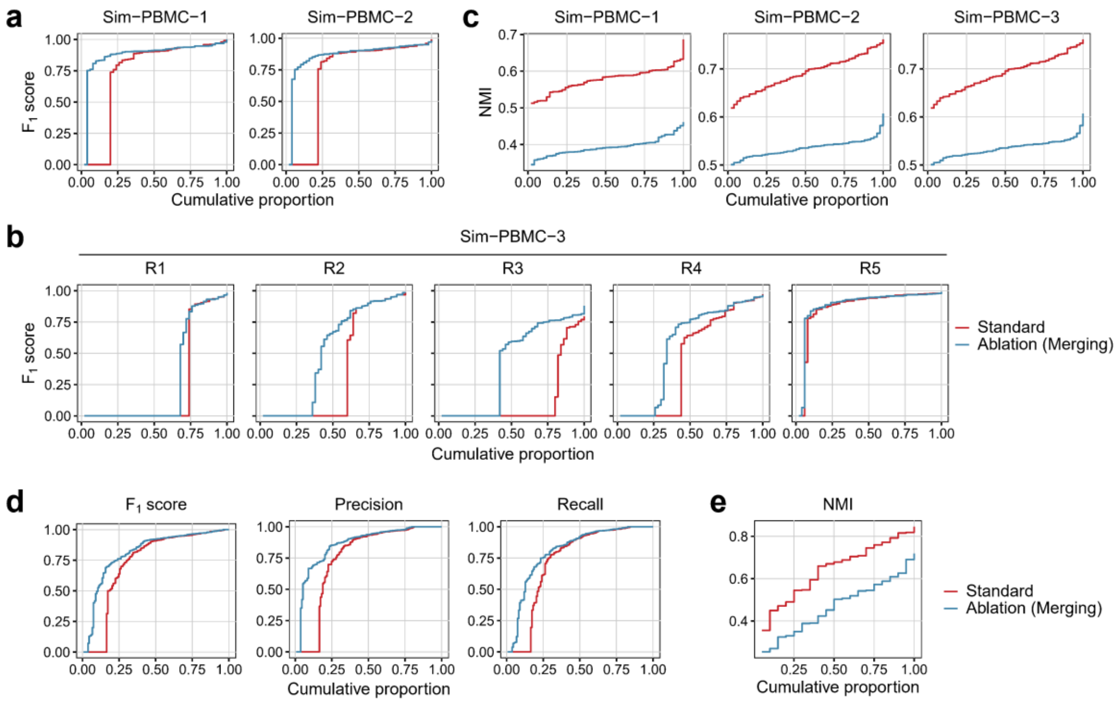

Supplementary Fig. 66 Ablation analysis of the merging component demonstrates its contribution to guarantee the clustering quality. a. Cumulative curves comparing F_1 scores before and after merging step ablation in simulated datasets 1 and 2. **b.** Same as (**a**), but based on simulated dataset 3. **c.** Same as (**a**), but comparing NMI in ablation analysis across three simulated datasets. **d.** Cumulative curves comparing F_1 scores before and after merging ablation in 20 real scRNA-seq datasets. **e.** Same as (**d**), but comparing NMI.

Supplementary Note 2 Benchmarking RareQ against existing rare cell detection methods with different parameter combinations.

To assess whether our conclusions depend on a particular "best-case" configuration, we performed a broad parameter-range sanity check across 20 scRNA-seq datasets. For each method (including RareQ), we enumerated and evaluated multiple parameter combinations drawn from the method's documented/author-recommended ranges (**Supplementary Fig. 67a, b**). Specifically, we evaluated the following parameter grids: RareQ (*k*: 6, 8, 10; Q_threshold: 0.1–0.9; ratio: 0.1–0.9), scCAD (merge_h: 0, 25, 50, 75, 90; overlap_h: 0.5–0.9), CellSIUS (min_n_cells: 5, 10, 15, 20, 25; min_fc: 1, 2, 3), RaceID (clustnr: 20, 22, 24, 26, 28, 30), GiniClust2 (Gini.pvalue_cutoff: 0.001, 0.0001; NumTopGene: 1,000–3,000), FiRE (HVG_num: 1,000, 2,000; *M*: 10, 20, 50, 100, 200), EDGE (n_dm: 5, 10, 20; n_neigs: 10, 15, 20, 25), and GapClust (*k*: 150–250). Across these diverse configurations, RareQ consistently achieves the strongest overall performance.

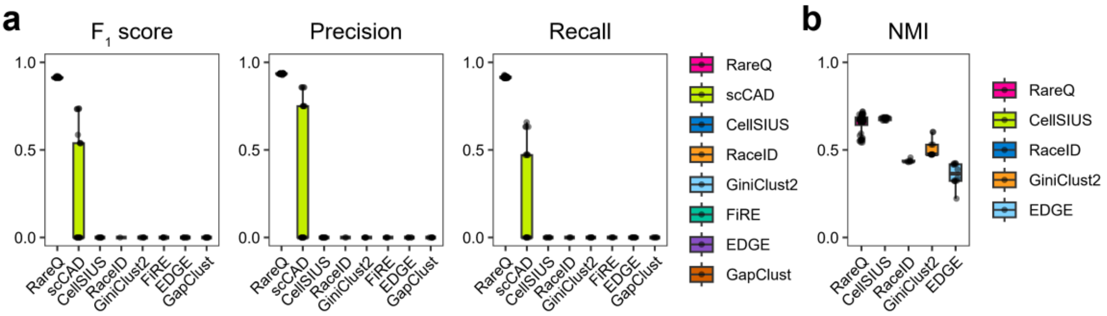

Supplementary Fig. 67 Benchmarking RareQ against existing methods with different parameter combinations in 20 scRNA-seq datasets. a. Boxplots summarizing rare-cell detection performance across multiple sampled parameter combinations for each method. Each dot represents the result for a given method and parameter setting, with metric values aggregated across all rare cell types in the 20 datasets. **b.** Same as (**a**), but evaluating global clustering performance via NMI.

Supplementary Note 3 Benchmarking RareQ against Seurat across resolution settings. To evaluate whether RareQ's performance can be replicated by simply tuning the resolution parameter of a widely used modularity-based clustering pipeline (Seurat's Louvain and Leiden clustering), we compared Louvain and Leiden with RareQ across 20 scRNA-seq datasets. For each dataset, both methods were applied to the same kNN graph to ensure a fair and controlled comparison. RareQ was run with default parameters, whereas Louvain and Leiden clustering were evaluated over a broad range of resolution values (0.5, 0.8, 1, 2, 3, 4, 5, 6, 7, 8, 9, 10, 15, 20), with the highest settings included as a stress test beyond typical Seurat usage.

Across datasets, increasing the resolution improved Louvain's rare-population detection, and at very high resolutions (15–20) its rare-cell detection performance approached that of RareQ (F_1 score) (**Supplementary Fig. 68a**). However, this gain came at a substantial cost: global clustering consistency deteriorated markedly, as reflected by substantially reduced NMI values. Consistently, the number of inferred clusters increased sharply at high resolutions, indicating pronounced over-clustering and excessive fragmentation of major populations (**Supplementary Fig. 68b**). Similar results were observed when comparing RareQ against Leiden algorithm (**Supplementary Fig. 69a, b**). Together, these results show that while aggressive resolution tuning may partially recover rare populations, it does so by over-partitioning the global clustering structure.

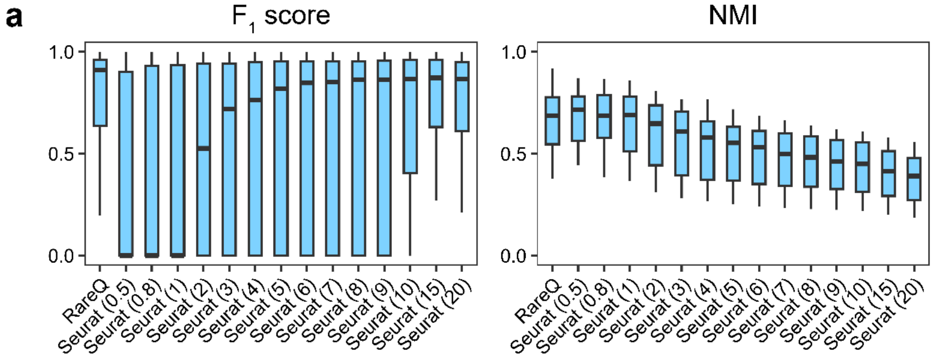

b

	Data	Orig.	RareQ	Seurat resolutions													
				0.5	0.8	1	2	3	4	5	6	7	8	9	10	15	20
1	Airway	7	20	10	13	14	19	24	31	36	43	49	57	67	72	137	232
2	Arc-ME	11	64	22	25	27	35	38	45	52	55	61	67	74	78	104	139
3	B_lymphoma	14	24	12	15	18	24	30	37	43	47	56	67	74	86	154	242
4	Cao	41	46	29	35	37	43	49	56	65	73	85	96	119	137	195	231
5	Chen	46	57	19	21	24	30	35	40	45	55	60	69	78	91	169	292
6	Choroid_plexus	26	61	17	18	18	27	35	41	47	55	63	69	78	85	145	231
7	Cortex	13	32	14	16	18	23	26	31	43	58	83	122	160	201	336	325
8	Heart	8	26	10	15	15	23	26	33	38	48	50	55	62	75	109	164
9	Kidney_ccRCC	14	65	23	27	29	35	43	48	55	61	68	70	75	78	104	133
10	Kidney_normal	28	51	17	20	22	32	39	42	50	54	59	65	71	73	100	141
11	MacParland	20	24	14	17	19	23	26	27	33	41	48	57	68	81	177	281
12	Macosko	12	81	16	23	25	42	61	78	98	115	139	156	182	206	348	570
13	Mammary	20	51	16	20	23	28	35	42	47	52	58	67	76	79	126	178
14	Pancreas	14	29	14	15	18	23	28	30	35	39	46	53	63	68	94	138
15	Pediatric_gut	41	96	26	32	35	44	50	60	66	73	81	88	94	104	123	152
16	Plasschaert	8	15	10	11	13	19	28	34	43	52	66	86	106	133	303	485
17	Retina	16	42	16	18	23	32	54	91	145	234	327	421	492	572	805	892
18	Shekhar	19	28	16	17	20	21	25	32	44	53	64	77	91	108	236	454
19	UUOkidney	17	13	10	11	12	16	21	29	38	53	69	89	121	159	367	580
20	Zelsel	7	30	10	11	13	19	28	33	35	39	42	48	50	56	86	111

Supplementary Fig. 68 Benchmarking RareQ against Seurat (Louvain algorithm) with different resolutions in 20 scRNA-seq datasets. a. Boxplots comparing RareQ's F_1 score and NMI with Seurat (Louvain algorithm) with different resolutions. **b.** Number of original cell types and clusters inferred by RareQ and Seurat (Louvain algorithm) in (**a**).

a

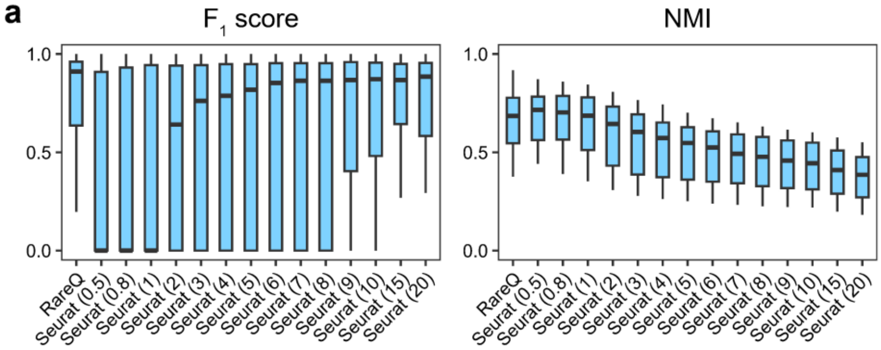

b

	Data	Orig.	RareQ	Seurat resolutions													
				0.5	0.8	1	2	3	4	5	6	7	8	9	10	15	20
1	Airway	7	20	11	13	15	20	26	32	39	46	53	63	73	78	146	250
2	Arc-ME	11	64	22	26	26	35	42	46	53	56	62	70	76	80	116	145
3	B_lymphoma	14	24	12	15	19	24	31	38	46	52	59	70	82	88	161	257
4	Cao	41	46	30	36	36	44	50	59	64	76	86	100	123	141	204	231
5	Chen	46	57	19	22	27	32	37	41	49	57	64	72	82	94	178	304
6	Choroid_plexus	26	61	18	18	19	27	36	42	50	59	68	73	80	93	149	241
7	Cortex	13	32	14	18	20	23	28	34	44	62	87	123	163	205	337	343
8	Heart	8	26	10	15	18	23	29	35	42	49	52	63	70	78	114	176
9	Kidney_ccRCC	14	65	23	28	28	35	45	50	60	64	69	72	77	81	110	144
10	Kidney_normal	28	51	18	21	22	32	40	44	51	58	63	67	71	79	106	143
11	MacParland	20	24	15	17	19	24	26	31	37	44	52	60	72	83	179	294
12	Macosko	12	81	19	25	29	48	67	83	100	120	138	160	191	213	373	593
13	Mammary	20	51	17	21	23	27	36	44	49	58	64	70	78	84	135	194
14	Pancreas	14	29	14	15	19	24	30	30	36	42	53	59	66	73	100	147
15	Pediatric_gut	41	96	28	33	36	45	52	63	69	80	86	94	99	106	129	163
16	Plasschaert	8	15	10	12	13	19	29	37	46	56	74	91	110	136	311	516
17	Retina	16	42	16	18	24	33	57	97	152	234	324	421	510	585	837	897
18	Shekhar	19	28	16	18	20	21	26	33	45	54	68	82	96	115	239	482
19	UUOkidney	17	13	10	12	12	18	22	31	39	53	75	95	130	165	379	601
20	Zelsel	7	30	10	12	13	20	29	33	37	39	46	50	52	60	89	120

Supplementary Fig. 69 Benchmarking RareQ against Seurat (Leiden algorithm) with different resolutions in 20 scRNA-seq datasets. a. Boxplots comparing RareQ's F_1 score and NMI with Seurat (Leiden algorithm) with different resolutions. **b.** Number of original cell types and clusters inferred by RareQ and Seurat (Leiden algorithm) in (**a**).

Supplementary Note 4 Sensitivity analysis of RareQ to key parameters.

We performed comprehensive sensitivity and robustness analyses to evaluate RareQ's dependence on both core algorithmic hyperparameters and upstream preprocessing choices.

Algorithmic parameters: We systematically varied k.param (10–30), *k* (5–30), Q_threshold (0.1–0.9), and the ratio parameter (0.1–0.9) across 150 simulated and 20 real scRNA-seq datasets. Across these broad ranges, RareQ consistently exhibited stable rare-cell detection and global clustering performance, indicating strong robustness to parameter choices (**Supplementary Fig. 24–27**). While performance was generally stable, we observed slightly improved accuracy within the following ranges: k.param = 10–20, *k* < 20, Q_threshold = 0.4–0.6, and ratio > 0.1.

Upstream preprocessing choices: To assess sensitivity to the feature space used to construct the kNN graph, we further evaluated RareQ on 20 real scRNA-seq datasets while varying the number of principal components (PCs) and the number of highly variable genes (HVGs) used for preprocessing. RareQ remained stable and achieved consistently strong performance when using sufficiently informative feature spaces, particularly when PCs ≥ 40 and HVGs ≥ 1,500 (**Supplementary Fig. 70**). Across these settings, RareQ's rare-population detection and global clustering performance showed minimal variation, indicating robustness under commonly used preprocessing regimes.

Together, these analyses demonstrate that RareQ is robust to both downstream hyperparameter choices and upstream preprocessing decisions, and maintains strong tolerance to technical noise across diverse simulated and real datasets.

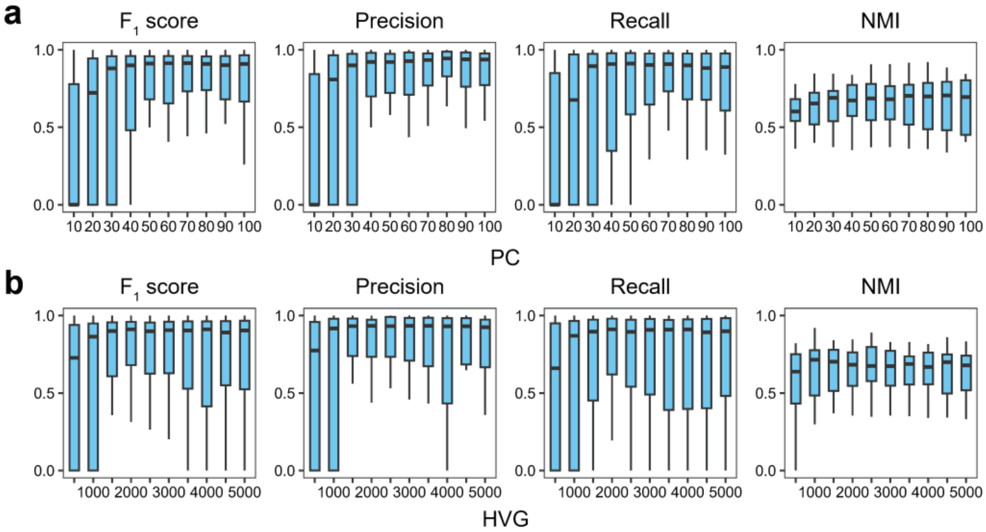

Supplementary Fig. 70 Robustness of RareQ to principle components (PCs) (a) and highly variable genes (HVGs) choices across real scRNA-seq datasets (b).

Supplementary Note 5 Robustness of RareQ to cell-index shuffling (tie-breaking analysis). When a cell has multiple neighbors with the same highest Q value, it selects the neighbor with the smallest cell index. To test whether this rule introduces sensitivity to the arbitrary ordering of cells, we performed a robustness analysis by systematically reshuffling cell indices across 150 simulated and 20 real scRNA-seq datasets. RareQ's rare-cell detection and global clustering performance remained highly consistent across reshuffled replicates, indicating strong robustness to cell-index permutation (**Supplementary Fig. 28**).

To further enhance flexibility, we have implemented an optional non-deterministic tie-break mode, enabling users to perform multiple random runs and derive consensus clusters, which can yield more refined and higher-precision cluster assignments when desired.

Supplementary Note 6 Evaluating the impact of batch effects and batch correction on RareQ's performance.

To assess how batch effects can influence RareQ's rare-population calls, we designed a controlled batch-only rarity stress test using simulated scRNA-seq datasets with graded batch strengths. Specifically, we generated datasets containing two biological cell types and two batches (~1,500 cells total). We constructed an adversarial scenario in which one batch within cell type 1 accounts for only 1% of all cells, whereas the remaining batch of the same cell type and both batches of cell type 2 each comprise ~33% of cells. This setup mimics a realistic failure mode where a rare batch subset (rather than a true biological rare type) may be spuriously detected as a "rare population" if batch effects are not corrected (**Supplementary Fig. 71a**). Batch-effect strength was systematically increased by varying Splatter's batch.facLoc parameter from 0.1 to 1.0.

We applied RareQ to these datasets under (i) no batch correction and (ii) several commonly used batch-correction strategies, including CCA, RPCA, SCT integration, and Harmony. Without batch correction, RareQ consistently identified the rare batch subset as a rare population in this batch-only rarity setting, yielding false positive rate (FPR) = 1. In contrast, after batch correction, RareQ largely avoided this failure mode when batch effects were mild to moderate, resulting in low FPR across the tested integration strategies (**Supplementary Fig. 71b, c**).

Notably, under extremely strong batch effects, batch correction can over-collapse biological distinctions and lead to batch-confounded clusters, reflecting confounding between batches and cell types. Among the tested approaches, CCA and SCT integration showed the most favorable behavior in mitigating batch-driven artifacts while better preserving biological structure. Overall, these results support a clear practical recommendation: when batch effects are present, applying batch correction is critical to prevent spurious "rare populations" driven primarily by batch composition rather than biology.

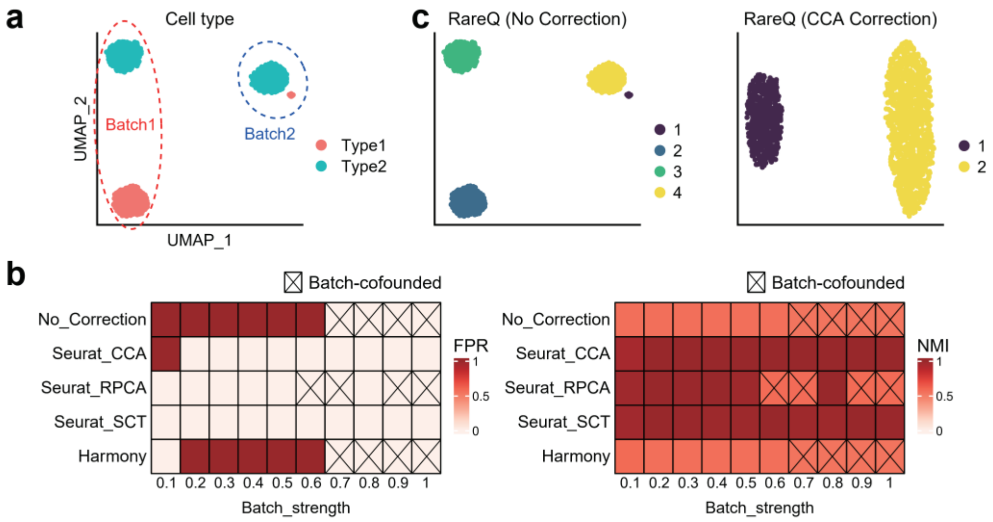

Supplementary Fig. 71 Evaluating the effect of batch effect correction on rare cell detection by RareQ. a. UMAP of simulated data with two cell types from two batches by

Splatter. **b.** False positive rate (FPR) and clustering accuracy (NMI) of RareQ on ten simulated datasets with varying batch strength using different batch effect correction strategies. **c**, UMAPs displaying the clustering of RareQ with and without batch effect correction.

Supplementary Note 7 Evaluating RareQ's performance on negative control dataset.

To further quantify the FPR under a "no true rare types" scenario, we constructed a negative-control dataset by extracting low-heterogeneity major populations from the UUOkidney dataset. This control dataset is designed to lack genuine rare populations. Across a range of PCs and HVG settings, RareQ produced near-zero FPR, indicating that RareQ does not tend to invent rare clusters in relatively homogeneous data when true rare populations are absent (**Supplementary Fig. 72a**). Additionally, we provide practical diagnostics that users can apply when ground-truth labels are unavailable. In the UUOkidney–derived negative-control dataset, *Q*-value distributions are similar across clusters, consistent with the absence of a topologically distinct rare clique (**Supplementary Fig. 72b**). In contrast, when we spiked in a known rare population (e.g., JGA cells), the rare population exhibits substantially elevated *Q* values compared to other clusters, consistent with the intended topological signature that RareQ leverages (**Supplementary Fig. 72c**).

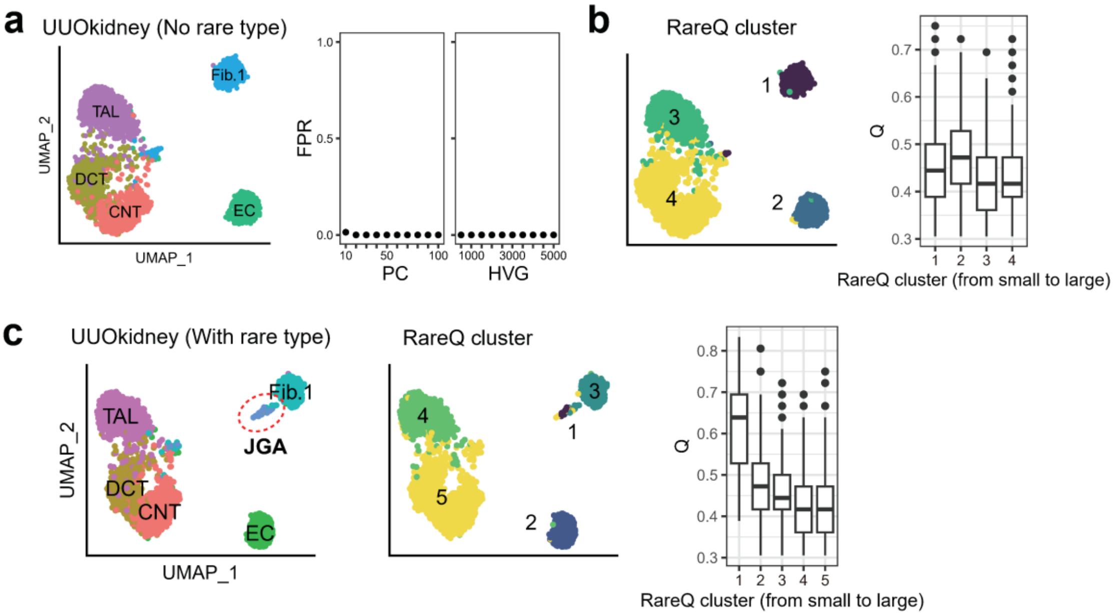

Supplementary Fig. 72 Evaluating RareQ's performance in negative control data. **a**, Negative-control dataset constructed from low-heterogeneity major cell populations extracted from the UUO kidney dataset; across a range of PCs and HVG settings, RareQ maintains near-zero FPR. **b**, In the UUO-kidney–derived negative-control dataset, *Q*-value distributions are comparable across clusters. **c**, *Q*-value–based diagnostic after spiking in a true rare population (JGA cells).
